# Mechanistic Insight Into Cadmium- and Zinc-Induced Inactivation of the *Candida albicans* Pif1 Helicase

**DOI:** 10.3389/fmolb.2021.778647

**Published:** 2022-01-21

**Authors:** Bo Zhang, Qintao Zhang, Xinting Zhu, Dayu Li, Xiaolei Duan, Jiao Jin, Kejia Wang, Yan Xie, Yang Liu

**Affiliations:** ^1^ College of Basic Medicine, Zunyi Medical University, Zunyi, China; ^2^ Medical Laboratory, Affiliated Hospital of Zunyi Medical University, Zunyi, China; ^3^ College of Life Sciences, Guizhou University, Guiyang, China; ^4^ School of Public Health, Zunyi Medical University, Zunyi, China

**Keywords:** zinc, cadmium, CaPif1, ATPase, DTT, helicase, heavy metal, toxicity

## Abstract

Zinc and cadmium are environmental contaminants that can cause disease by affecting the activity of DNA-repair proteins. In this study, we investigated the effect of Zn^2+^ and Cd^2+^ on the *Candida albicans* Pif1, a DNA-repair helicase that plays a critical role in ensuring genomic stability. We show that Zn2+ and Cd2+ strongly inhibit both the ATPase and the unwinding activities of CaPif1, but have no effect on its DNA binding activity. High concentrations of Cd2+ may bind to the cysteine residues of CaPif1, and its inhibition appears to be difficult to be restored by ethylene diamine tetraacetic acid, while inhibition due to Zn^2+^ can. When the two ions are at low concentrations, increasing the concentration of ATP in the reaction can appropriately weaken the inhibitory effect of Zn^2+^, while cysteine can reduce the inhibitory effect of Cd^2+^. In addition, we found that for both Cd^2+^ and Zn^2+^ the inhibition effects were nearly 100 times greater in reduced environments than in non-reducing environments. When heavy metals stimulate the body’s response, the environment of the body becomes less reducing, and thus the tolerance of CaPif1 to heavy metals will be stronger. We propose that CaPif1 may resist the toxicity of heavy metals through this mechanism. Altogether, our results provide new insights into the mechanisms by which heavy metals are toxic to DNA-repair proteins.

## Introduction

With the development of society, an increasing amount of heavy metals are released into the environment through industry and agriculture. Heavy metals tend to bioaccumulate, display biomagnification, and are toxic, which can lead to a variety of human diseases. They damage the kidneys, cause harm to the lungs upon inhalation, and interfere with bone metabolism (Maret and Moulis, 2013). Zinc’s chemical and physical properties are similar to those of cadmium (Brzoska, 2001), but the biological effects of these two heavy metals are quite different. Zinc is required for biological functions (Andreini et al., 2004), as it protects genetic stability and is a key component of proteins functioning in the antioxidant mechanism and DNA repair (Yildiz et al., 2019). In contrast, it has been proven that cadmium causes cancer.

Previous studies have shown that cadmium is a weak denaturing agent. It does not directly cause obvious DNA damage and seems to operate indirectly, but the exact molecular mechanism is unclear (Waisberg et al., 2003). The principal mechanisms of cadmium carcinogenicity are 1) the inhibition of DNA repair (Hengstler et al., 2003); 2) the interference with the antioxidant defense system that stimulates the production of reactive oxygen species (ROS), which directly induce DNA damage through oxidizing nucleoside bases and indirectly affect DNA repair (Jomova and Valko, 2011; Srinivas et al., 2019); 3) the regulation of cellular signal transduction pathways (Waisberg et al., 2003). The first and second mechanisms are associated with DNA-repair proteins. Zinc can reduce cadmium toxicity, and increasing the zinc supply may reduce cadmium absorption and accumulation, thus preventing or reducing the adverse actions of cadmium, whereas zinc deficiency can intensify cadmium accumulation and toxicity (Brzoska MM 2001; Yu et al., 2021). Interestingly, zinc decreases cadmium toxicity is related to DNA repair. Zinc contributes to an efficient DNA repair system, thereby alleviating DNA damage. Furthermore, zinc can reduce oxidative stress in cells (Yildiz et al., 2019). Thus, the role of zinc and cadmium is closely linked to DNA-repair proteins.

Pif1, a DNA-repair protein with a zinc finger structure, has a helicase activity that plays a critical role in ensuring genomic stability by promoting mitochondrial DNA stability (Geronimo et al., 2018), regulating ribosomal DNA replication (Ivessa et al., 2000) and telomerase activity, among other roles (Boule et al., 2005). Pif1 is closely linked to cancer, and it affects the apoptosis of neuroblastoma cells and cervical cancer cells (Chen et al., 2020; Wang et al., 2020). On the whole, it is of great biological significance to study the toxic effects of heavy metals on Pif1.

Pif1 is found in almost all eukaryotes, and the Pif1 family is highly conserved from yeasts to humans (Futami et al., 2007) . In this study, we investigated and characterized the biochemical activity and substrate specificity of *Candida albicans* Pif1 (CaPif1) and the impact of six heavy metal ions, manganese (Mn^2+^), calcium (Ca^2+^), zinc (Zn^2+^), nickel (Ni^2+^), copper (Cu^2+^), and cadmium (Cd^2+^), on its activity. We found that Zn^2+^, Ni^2+^, and Cd^2+^ strongly inhibited the unwinding activity of CaPif1, but had no obvious effect on its DNA binding activity. The inhibitory effects of Zn^2+^ and Cd^2+^ on CaPif1 were different, especially on its helicase functions: 1) it was difficult to restore the damage caused to CaPif1 in high concentrations of Cd^2+^ using ethylene diamine tetraacetic acid (EDTA), but not under Zn^2+^; 2) the inhibitory effect of Cd^2+^ was more serious than that of Zn^2+^; 3) cysteine (Cys) could mitigate the damage caused by Cd^2+^, and ATP could alleviate the inhibition of Zn^2+^ in the low concentration range. Even more surprisingly, CaPif1 could effectively resist the inhibitory effects of these heavy metals in a low reducing environment. These findings provide a new understanding of the toxic molecular mechanisms of metal accumulation in organisms leading to a variety of human diseases.

## Materials and Methods

### Chemical Reagents

CdCl_2_, ZnCl_2_, MgCl_2_, MnSO_4_, CaCl_2_, NiSO_4_, CuSO_4_, EDTA, dithiothreitol (DTT), sodium dodecyl sulfate(SDS), nucleotide triphosphates (NTPs), and deoxynucleotide triphosphates (dNTPs) were purchased from Sangon Biotech (Shanghai) Co., Ltd. Amino acids, ATP Assay Kit was purchased from Beyotime Biotechnology (Shanghai, China).

### Recombinant Proteins

The open reading frame encoding the CaPif1 protein (NCBI number: AOW31431.1) was generated by PCR and cloned into a modified pET-21a vector with an N-terminal SUMO tag to generate His-SUMO-CaPif1. The construct was transformed into *E. coli* C2566H. The cells were grown in LB medium containing 100 μg/ml ampicillin at 37°C to an OD_600_ of 0.7 and induced with 0.3 mM IPTG at 18°C for an additional 16 h. The cells were then harvested by centrifugation, resuspended in a buffer [20 mM Tris-HCl (pH 7.6), 500 mM NaCl], and sonicated 2–3 times to shear their DNA. All purification steps were performed at 4°C. After centrifugation and filtration using a 0.45 μm membrane, the mix was applied to a HisTrap Ni-Sepharose column (GE Healthcare) and eluted in a gradient increased to 200 mM imidazole over 15 column volumes. The His-SUMO tag was removed by SUMO enzyme. The mixture of proteins was dialyzed overnight against 20 mM Tris-HCl (pH 7.6), and 500 mM NaCl, and applied to the second round of Ni-sepharose chromatography. Finally, the purified protein fractions were concentrated to 10 mg/ml. Glycerol was added at a final concentration of 15%, and the proteins were stored at −80°C.

### Oligonucleotides

All DNA substrates used in the experiment were PAGE-purified and purchased from GENEWIZ laboratory (Jiangsu, China). The sequences of unlabeled or fluorescently labeled DNA are shown in Table 1. All of the labeled DNA involved in the experiment was labeled with 3′-fluorescein (3′-FAM). Double-stranded DNA substrates were obtained by mixing equimolar concentrations of complementary single-stranded oligonucleotides in 50 mM Tris-HCl (pH 7.5) and 25 mM NaCl. The mixture was heated to 95°C for 5 min, and annealing was allowed by slow cooling to room temperature. Finally, series of duplex DNA samples were stored at −40°C.

**TABLE 1:** . Structures and sequences of the DNA substrates.

Name	Structure	Sequence (F, fluorescein)	Comment
S43	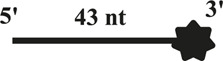	5′-CTGTAGGAATGTGAAATAAAAACGATGTTTTATTTACATTGTA-3′-F	43nt ssDNA
S21	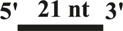	5′–TAC​AAT​GTA​AAT​AAA​ACA​TCG-3′	21nt ssDNA
S40	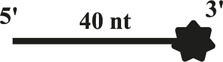	5′-CCATGCAGCTGTCAGTCCATTGTCATGCTAGGCCTACTGC-3′-F	40nt ssDNA
DS32	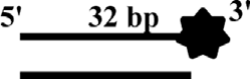	5′-TATCGAAGAATGTTATGTCATTCCGGCAGATG-3′-F	32bp dsDNA
		3′-ATA​GCT​TCT​TAC​AAT​ACA​GTA​AGG​CCG​TCT​AC-5′	
OhS22D21	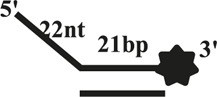	5′-CTGTAGGAATGTGAAATAAAAACGATGTTTTATTTACATTGTA-3′-F	5′-22nt-Overhanged-21bp
		3′-GCT​ACA​AAA​TAA​ATG​TAA​CAT-5′	
BS12	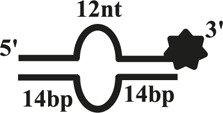	5′-CCATGCAGCTGTCAGTCCATTGTCATGCTAGGCCTACTGC-3′-F	Bubble-12nt
		3′-GGT​ACG​TCG​ACA​GTG​TCC​ATT​GTC​ATC​GAT​CCG​GAT​GAC​G-5′	
YS22	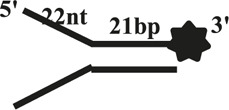	5′-CTGTAGGAATGTGAAATAAAAACGATGTTTTATTTACATTGTA-3′-F	Y-structure-22nt
		3′-AAA​AAA​AAA​AAA​AAA​AAA​AAA​AGC​TAC​AAA​ATA​AAT​GTA​ACA​T-5′	
OhS4D20	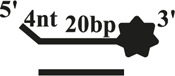	5′-GCC​CTG​GTG​CCG​ACA​ACG​AAG​GTA-3' -F	5′-4nt-Overhanged-20bp
		3′-ACC​ACG​GCT​GTT​GCT​TCC​AT-5′	
OhS12D20	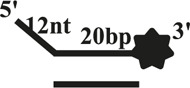	5′–TAT​CGA​AGA​ATG​TTA​TGT​CAT​TCC GGCAGATG-3′-F	5′-12nt-Overhanged-20bp
		3′-AAT​ACA​GTA​AGG​CCG​TCT​AC-5′	
OhS20D20	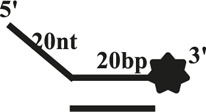	5′-CCATGCAGCTGTCAGTCCATTGTCATGCTAGGCCTACTGC-3′-F	5′-20nt-Overhanged-20bp
		3′-ACA​GTA​CGA​TCC​GGA​TGA​CG-5′	
OhS12D12	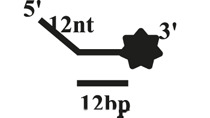	5′-GCCCTGGTGCCGACAACGAAGGTA-3′-F	5′-12nt-Overhanged-12bp
		3′-TGTTGCTTCCAT-5′	
OhS12D28	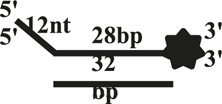	5′-CCATGCAGCTGTCAGTCCATTGTCATGCTAGGCCTACTGC-3′-F	5′-12nt-Overhanged-28bp
		3′–GTC​AGG​TAA​CAG​TAC​GAT​CCG​GAT​GAC​G-5′	

### Annealing Assays

To determine the annealing activity of CaPif1, we used two partially complementary single-stranded DNA (10 nM) samples mixed with different concentrations of CaPif1 in annealing buffer [25 mM Tris-HCl (pH 7.5), 50 mM NaCl, 1.5 mM MgCl_2_, 1 mM DTT]. No ATP was used because DNA annealing is usually ATP-independent, while unwinding depends on ATP hydrolysis. If the solution contains ATP, the double-stranded DNA produced by annealing will be unwound, so that annealing cannot be observed.

After incubation at 30°C for 10 min, adding 5 × stop loading buffer (150 mM EDTA, 2% SDS, 30% glycerol, and 0.1% bromophenol blue) to stop the reaction. The samples were electrophoresed on a 12% native polyacrylamide gel and visualized with ChemiDoc MP (Bio-Rad, CA, United States).

### DNA Binding Assays

Fluorescence polarization assay: The binding of CaPif1 to DNA was monitored by fluorescence polarization assay using the SpectraMax iD3 microplate reader (Molecular Devices, LLC, PA, United States). following previously reported methods (Zhang et al., 2016). Briefly, the fluorescence-labeled DNA substrate (5 nM) was mixed with varying amounts of CaPif1 in binding buffer [25 mM Tris-HCl (pH 7.5), 50 mM NaCl, 1.5 mM MgCl_2_, and 1 mM DTT] at 25°C for 10 min. Then, the fluorescence polarization was measured. The anisotropy value of each test point was averaged by three independent tests.

Electrophoretic mobility shift assay (EMSA): The 3′-fluorescein labeled DNA substrates (10 nM) were pre-incubated with varying amounts of CaPif1 in binding buffer at 25°C for 5 min. Before electrophoresis, 5 × loading buffer (60% glycerol and 0.1% bromophenol blue) was added into the mixture. The electrophoresis conditions were as follows: native PAGE (Acr∶Bis = 39∶1), 6% polyacrylamide, 100 V, 40 min. The results were visualized by a ChemiDoc MP Imaging System (Bio-Rad, CA, United States).

### Helicase Assay

CaPif1 was combined with duplex DNA in unwinding buffer [25 mM Tris HCl (pH 7.5), 50 mM NaCl, 1.5 mM MgCl_2_, 1 mM DTT, 5 mM ATP], and ATP was added to initiate the reaction at 30°C for 10 min. The reactions were quenched with the addition of 5 × stop loading buffer. Products of DNA unwinding reactions were resolved on native PAGE 12% (Acr∶Bis = 39∶1) gels, 100 V, 80 min. DNA in polyacrylamide gels was visualized using a ChemiDoc MP and quantitated using the Image Lab software (Bio-Rad). The percentage of unwound helicase substrate was calculated using the following Eq. 1, where P is the product (ssDNA), and S is the substrate (dsDNA):

% unwinding=100%×P/(S+P). (1)

### ATPase Assay

ATPase activity was detected using a commercial ATPase assay kit according to manufacturer’s instructions (Beyotime Biotechnology, Shanghai, China). The wavelength was 540 nm, and the cumulative detection time was 10 s. The experiment was repeated three times and the percentage of ATP consumption was calculated using the following Eq. 2:

% consumption=(P−S)/P×100%, (2)

where P is the light value produced by the control group (without CaPif1), S is the light value produced by the experimental group (various CaPif1 concentrations), and P-S is the light value reduced by the ATP consumption of the experimental group. Luminance was detected using the SpectraMax iD3 microplate reader (Molecular Devices, LLC, PA, United States). The fitting of the combined characteristic curve was done using the software ORIGIN.

## Results

### Characterization of the Basic Biochemical Activity of CaPif1

**FIGURE 1:**
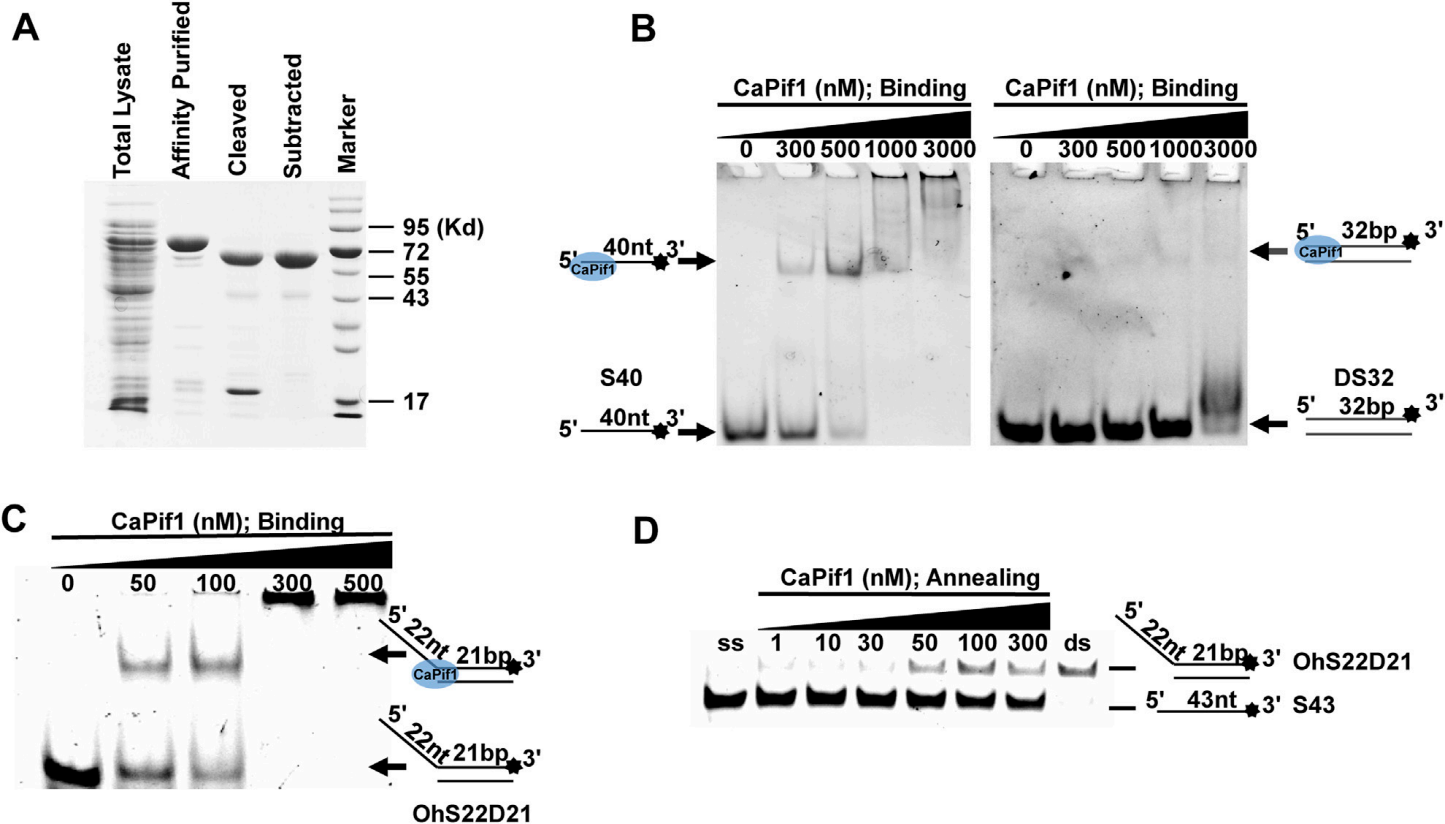
Binding and annealing activity of purification of CaPif1. **(A)** SDS-10% polyacrylamide gel stained with Coomassie brilliant blue dye. Lane marker: protein molecular mass markers (with masses, in kilodaltons, indicated on the right). **(B–C)** CaPif1 binding activity was detected by DNA of three substrates. Top of the arrow: CaPif1-DNA complexes; Bottom of the arrow: free DNA. Native PAGE (Acr: Bis = 39:1) 6%, 100 V, 40 min. **(D)** Annealing activity of CaPif1. Native PAGE (Acr: Bis = 39:1) 12%, 100 V, 80 min. Lane ds: OhS22D21; lane ss: S43 (see Methods for more details). The results of figures **(B–D)** were visualized by a ChemiDoc MP Imaging System (Bio-Rad, California, United States).

We successfully expressed and obtained recombinant CaPif1 through HisTrap affinity purification and cutting and subtracting SUMO tags in order to dissect its physiological functions (Figure 1A). Previous studies have shown that helicase will bind to the nucleic acid substrate in advance before the unwinding process (Witkiewicz-Kucharczyk and Bal, 2006). Therefore, in order to test the binding activity of CaPif1 to the nucleic acid substrate, different structures of DNA were selected: ssDNA (S40), blunt end dsDNA (DS32), and 5′- overhanged DNA (OhS22D21). We fixed three substrate concentrations at 10 nM, and CaPif1 at varying concentrations (0–3,000 nM) in binding buffer (Materials and Methods). As the concentration of CaPif1 increased, free DNA (bottom arrow in Figures 1B,C) gradually decreased; in contrast, the protein-DNA complex (up arrow in Figures 1B,C) gradually increased. Through comparison, it was found that CaPif1 with blunt end dsDNA had very weak binding activity, while the other two substrates containing single-stranded tail structures showed high affinity. When CaPif1 reached a certain high concentration, the protein-DNA complexes were found near the spot hole, indicating that the high concentration of CaPif1 may form dimer or polymer (Yu et al., 2021).

In addition, we tested whether CaPif1 had annealing activity (Figure 1D). Two partially complementary single-stranded oligonucleotides (S43 and S21 in Table 1, where S43 has been labeled with 3′-fluorescein) were incubated with different concentrations of CaPif1 (1–300 nM) in annealing buffer. As the concentration of CaPif1 increased, the annealed double-stranded DNA also increased. These results indicated that CaPif1 was competent for promoting DNA annealing.

### CaPif1 Unwinds More Specific Structured DNA

**FIGURE 2:**
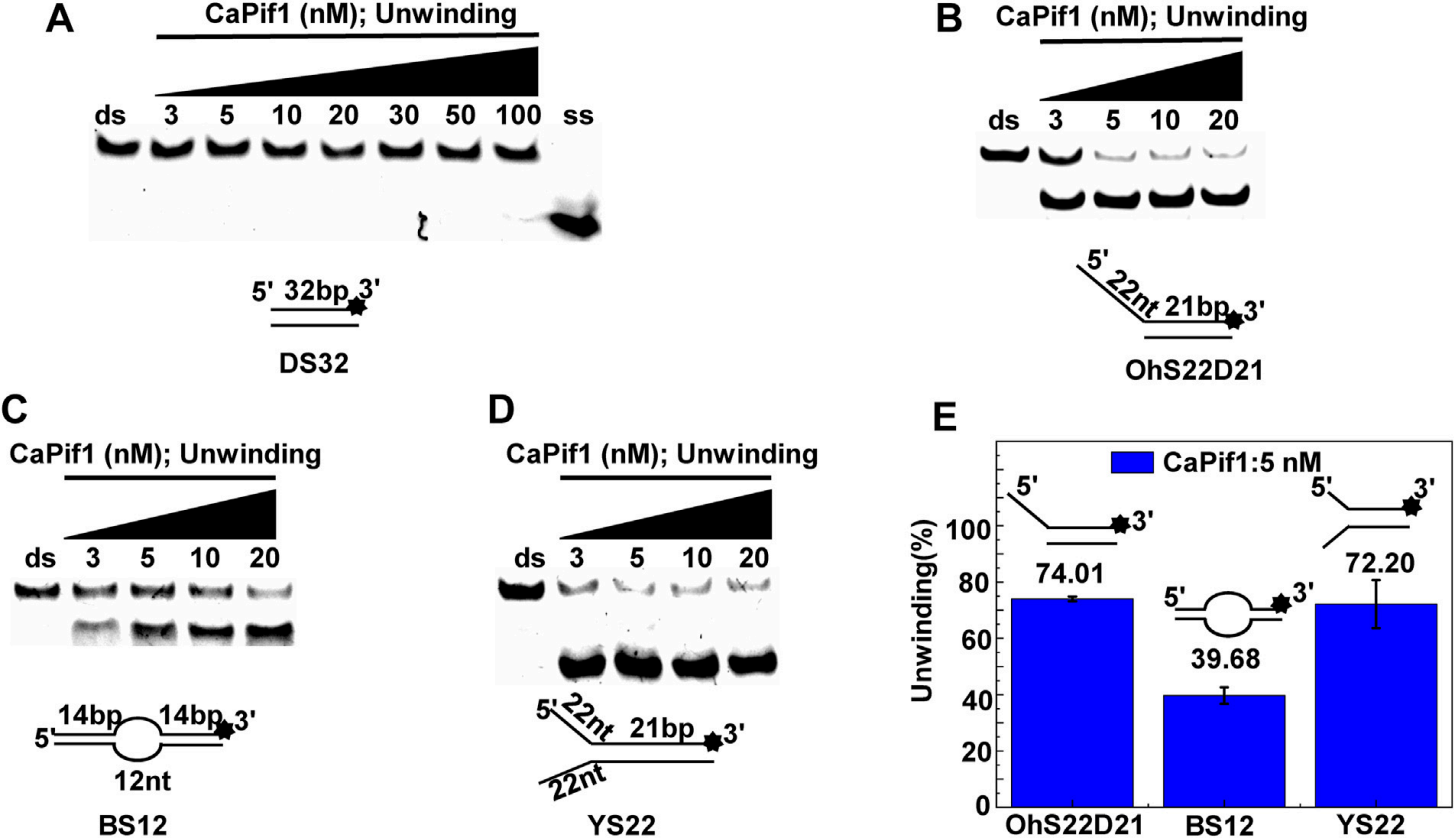
Unwinding preference of CaPif1. Unwinding characteristics of CaPif1 using various DNA substrates: **(A)** blunt end dsDNA, **(B)** 5′-overhanged, **(C)** Bubble, **(D)** Y-structure. **(A–D)** The experiments were performed using a series of double-stranded DNA substrates (shown in Table 1), fixed at 10 nM, and CaPif1 gradually increased. The unwinding reaction was initiated by adding 5 mM ATP at 30°C for 10 min. Native PAGE (Acr: Bis = 39:1) 12%, 100 V, 80 min. The fluorescence signals were visualized by a ChemiDoc MP Imaging System (Bio-Rad, California, United States). We calculated the proportion of unwinding DNA at 5 nM CaPif1 for a clearer analysis by scanning the gray values with Image Lab software **(E)** (Supplementary Table S1). All experiments were performed under standard experimental conditions as described in “Materials and Methods.”

The helicase of the Pif1 family unwinds a broad range of DNA substrates, a common feature for DNA structures is the presence of a 5′-ss tail. To probe the possible physiological functions of CaPif1 in the cell, different structured DNAs were labeled fluorescent groups (3′-FAM) to determine unwinding activity by EMSA, as shown in the figure (Figures 2A–D). Among the various DNA substrates tested, there was a common feature. CaPif1 could effectively unwind the DNA structures containing the 5′-ss tail, such as 5′-overhang (OhS22D21) and forked (YS22). Even at high concentrations (100 nM) of CaPif1, the blunt end dsDNA (DS32) structure still was not unwound. To note, the property that CaPif1 unwinds more efficiently 5′-ss tail DNA than that of Bubble (BS12) structure. The unwinding ratios are compared in (Figure 2E; Supplementary Table S1), and the sequences of these substrates are summarized in Table 1.

**FIGURE 3:**
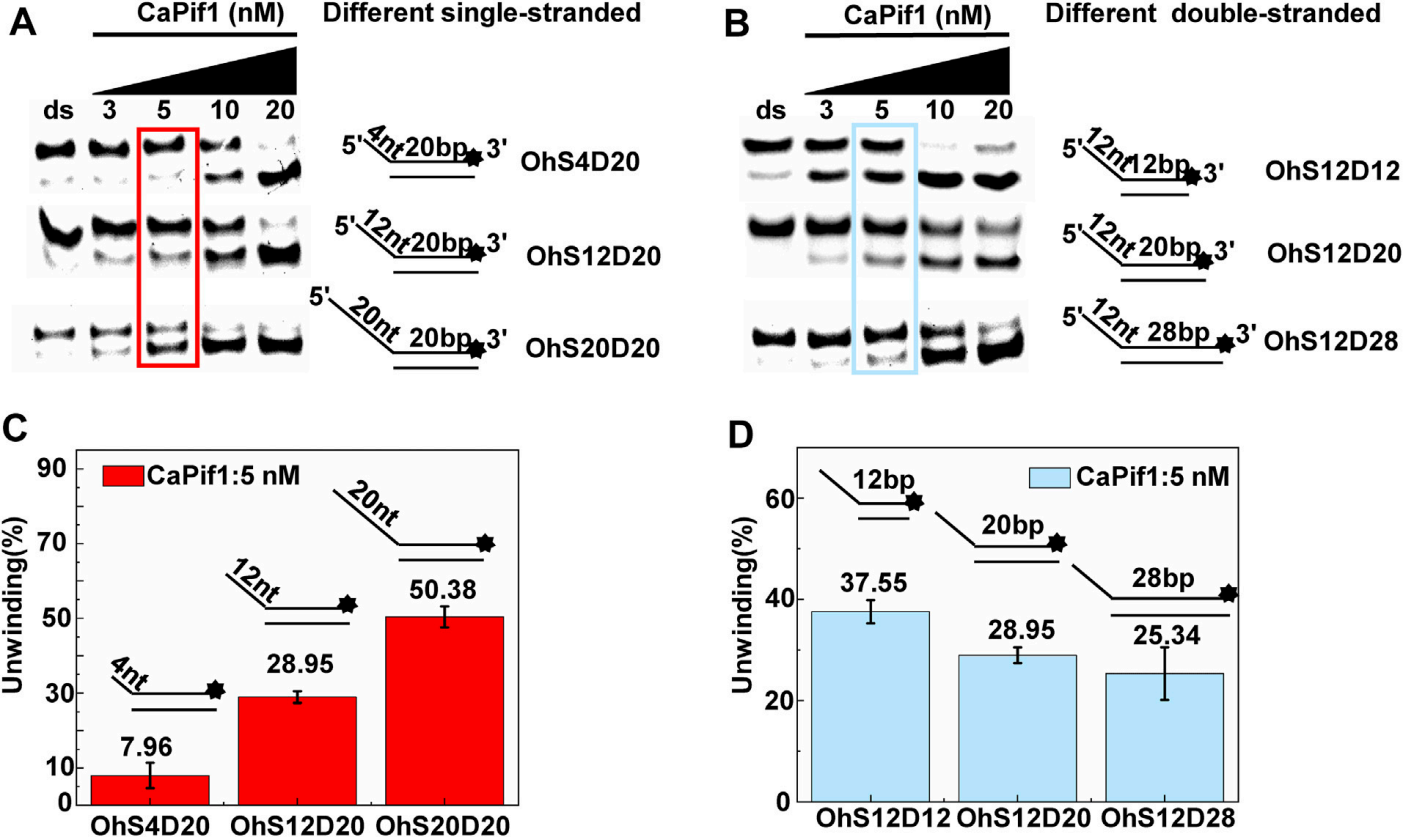
Comparison of the unwinding activity of CaPif1 with DNA substrates with different lengths. **(A)** A series of 20-bp dsDNA substrates with varying lengths of the 5′-tailed duplex DNAs, from 4 to 20 nt. **(B)** The 12 nt tailed duplex DNAs with varying lengths of the duplex region (from 12 to 28 bp) (see Table 1 for substrate sequences). **(C–D)** The bar chart based on the unwinding ratio of at 5 nM CaPif1 by scanning gray values with Image Lab software (Supplementary Table S2). All experiments were performed under standard experimental conditions as described in “Materials and Methods".

We further compared the effects of the 5′-ss tail and double-strand length on unwinding activity. Two sets of experiments were designed: in one set the double-strands were kept at the same length but the length of the 5′-ss tail gradually increased (OhS4D20, OhS12D20, and OhS20D20) (Figure 3A), while in the other set the 5′-ss tail length was fixed, but the double-strand length gradually increased (OhS12D12, OhS12D20, and OhS12D28) (Figure 3B). The results showed that the unwinding ratio of CaPif1 gradually decreased with the increase of the double-strand length, and the unwinding ratio of CaPif1 increased as the tail chain length increased (Figures 3C,D; Supplementary Table S2).

### A Preference for Adenosine Nucleotides of CaPif1

**FIGURE 4:**
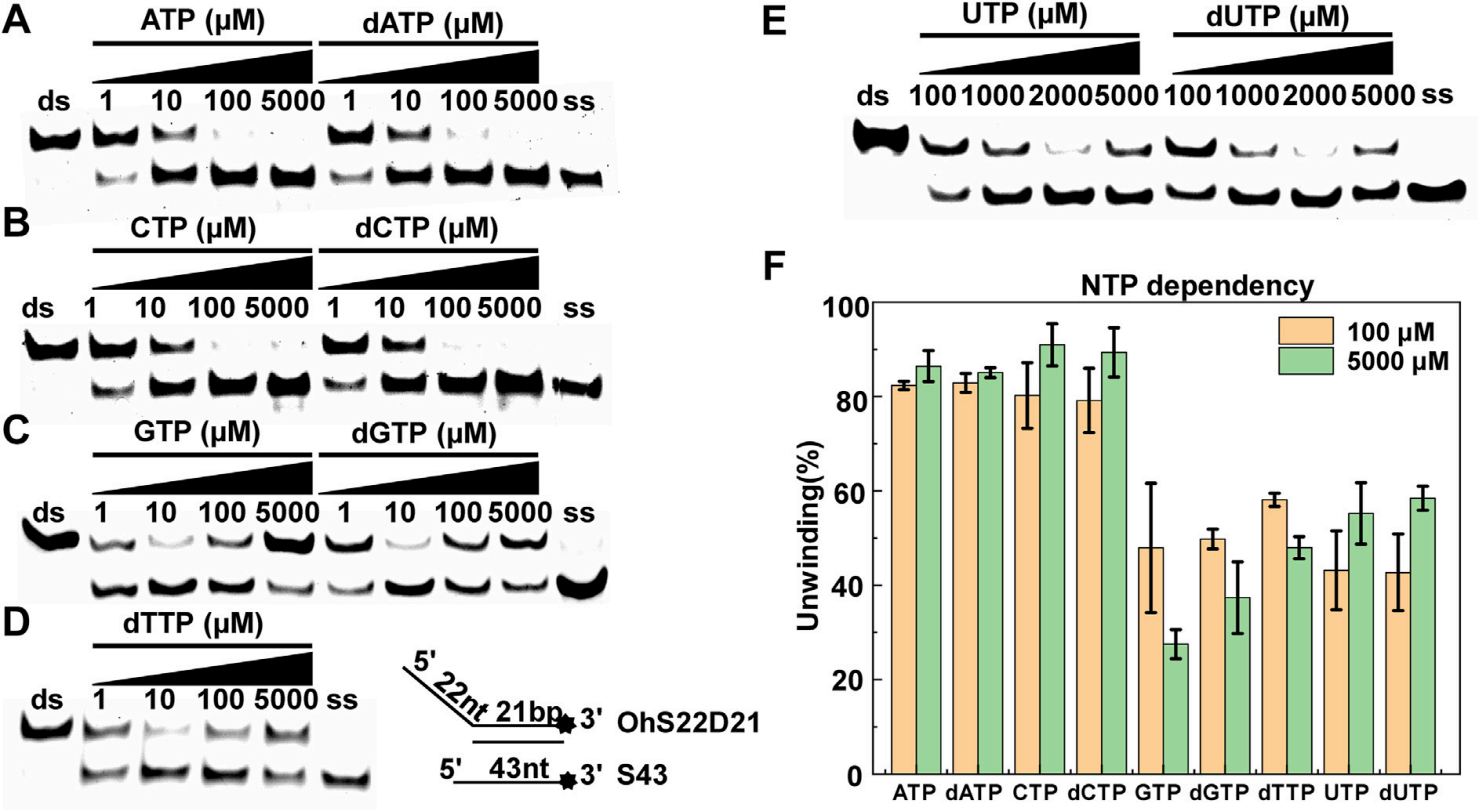
NTP/dNTP preference of CaPif1. DNA was unwound in the presence of the indicated NTP or dNTP **(A–E)**. The 5′-overhanged DNA substrate (OhS22D21) and CaPif1 concentrations were 25 nM, and the unwinding reaction was initiated with various NTP or dNTP concentrations. Lane ds: duplex DNA is not unwound, unwinding buffer (no ATP); Lane ss: single DNA with 3′-fluorescein (3′-FAM) (shown in Table 1). The numbers above the line indicate the concentrations of NTPs and dNTPs (μM). **(F)** The proportion of unwinding DNA (Supplementary Table S3).

Helicase can usually use the energy of hydrolyzing NTPs/dNTPs to melt double-stranded DNA, but whether CaPif1 has a preference for adenosine nucleotides as an energy source. Under the same experimental conditions, the unwinding activity of CaPif1 was measured in the presence of different types of NTPs and dNTPs. The results showed that CaPif1 could efficiently unwind the DNA in the presence of ATP, dATP, CTP, and dCTP, and 80% of DNA was unwound at 100 μM (Figures 4A,B). However, in the presence of GTP, dGTP, dTTP, UTP, and dUTP, less DNA was unwound. We found that when the concentration of these low-efficiency adenosine nucleotides was increased to 5 mM, the unwinding activity of CaPif1 was inhibited (Figures 4C–E). We speculate that for translocation helicases, the unwinding is dependent on the hydrolysis of NTPs or dNTPs, which depends on the ratio of NTPs/dNTPs to Mg^2+^ (Harmon and Kowalczykowski, 2001; Choudhary et al., 2004). Increasing the concentration of NTPs/dNTPs will disrupt the ratio and lead to hydrolysis inhibition, just as in NS3 helicase (Frick et al., 2007). These results indicate that CaPif1 can use the energy from the hydrolysis of a wide range of nucleotides to unwind DNA. We calculated the proportion of unwinding DNA at 100 and 5,000 μM NTP for a clearer analysis by scanning the gray values with Image Lab software (Figure 4F) (Supplementary Table S3).

### The Activity of CaPif1 is Affected by the Metal

**FIGURE 5:**
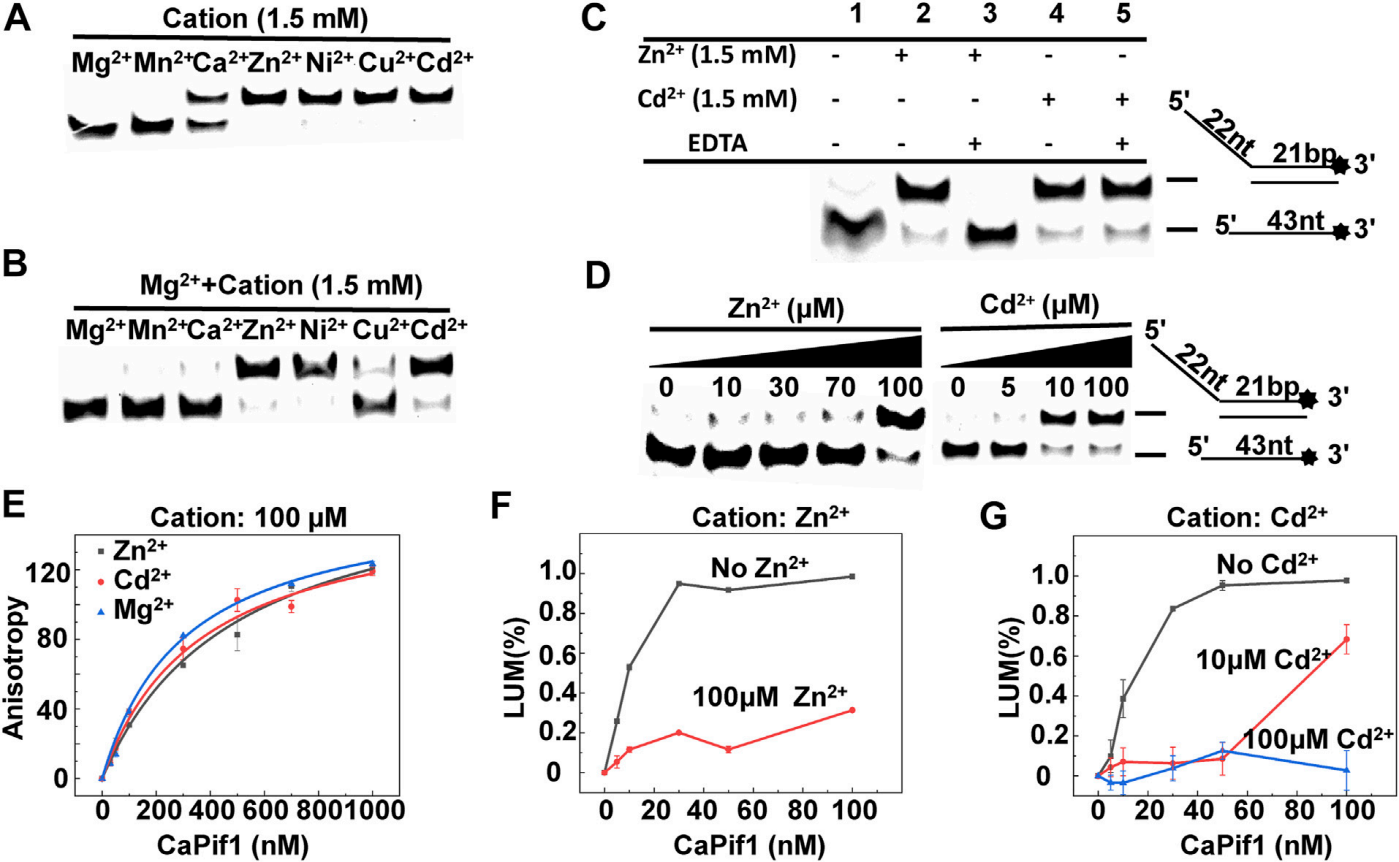
The effects of metals on the unwinding activity of CaPif1. **(A)** CaPif1 (25 nM) was reacted with the DNA OhS22D21 (10 nM) in the presence of the indicated divalent metal (1.5 mM). **(B)** CaPif1 was incubated with indicated divalent metal (1.5 mM) and then the same concentration of MgCl_2_ was added separately. **(C)** Lane 1: The control group containing 1.5 mM Mg^2+^ without Zn^2+^ and Cd^2+^; Lane 2 and Lane 4: same method (Figure 5B); Lane 3 and Lane 5: CaPif1 separately incubated with Zn^2+^ (1.5 mM) or Cd^2+^ (1.5 mM) at 30°C for 5 min, then add MgCl_2_ with a final concentration of 1.5 mM, incubate again at the same temperature for 5 min, and finally add the 5 mM of ATP to start the unwinding reaction. **(D)** Inhibit the unwinding activity of CaPif1 at low concentrations Zn^2+^ or Cd^2+^. A total of 10 nM of fluorescently labeled DNA substrate (OhS22D21) was titrated with increasing concentrations of Zn^2+^ or Cd^2+^. More details are described in the “Materials and Methods” section. **(E)** Polarized light is used to detect the influence of Zn^2+^, Mg^2+^, and Cd^2+^ on the binding activity of CaPif1, the ordinate is the anisotropy value, and the abscissa is the concentration of CaPif1. As detected by the Spectra Max iD3 microplate reader. **(F–G)** The influences of Zn^2+^ **(F)** or Cd^2+^ **(G)** on the ATPase. LUM (%) is the percentage of ATP consumed (CaPif1 group the control group*100%).

Helicases are usually fully and equally active for DNA unwinding in the presence of metal ions. Mg^2+^ and Mn^2+^ are common. Some metal ions are important for maintaining the protein structure. However, some metal ions destroy the protein structure and cause proteins to lose helicase activity. To address the influence of the metallic cofactor, we further determined the unwinding activity in the presence of various metal ions. Both Mg^2+^ and Mn^2+^ had higher unwinding activities, and the results were relatively similar, while Ca^2+^ supported CaPif1 to partially unwind the DNA duplex. In sharp contrast, in the presence of Zn^2+^, Ni^2+^, Cu^2+^, and Cd^2+^, this activity was completely lost at 1.5 mM (Figure 5A). This showed that CaPif1 was unable to use these four ions to melt double-stranded DNA.

To determine if the unwinding activity of CaPif1 was inhibited by the four ions, we first studied the reactions containing both Mg^2+^ and one of the other metal ions. We found that when CaPif1 was incubated in advance with solutions containing Zn^2+^, Ni^2+^, or Cd^2+^, even with the addition of sufficient Mg^2+^, CaPif1 could not unwind the DNA duplex, while in a reaction containing Cu^2+^, with the addition of Mg^2+^, the unwinding activity of CaPif1 could be clearly observed. The results indicate that although CaPif1 cannot use Cu^2+^ to unwind dsDNA, Cu^2+^ does not inhibit the unwinding activity of CaPif1; however Zn^2+^, Ni^2+^, and Cd^2+^ have significant inhibitory effects on the unwinding activity of CaPif1 (Figure 5B). Ni^2+^ research showed the same result as Cd^2+^, and also demonstrated strong inhibition. However, in the reaction containing Ni^2+^, a brownish-yellow precipitation occurred that made it impossible for us to make an accurate quantitative analysis of the inhibition of the unwinding activity of CaPif1 by Ni^2+^ (Supplementary Table S4.1).

We have also adjusted the different addition order of Zn^2+^/Ni^2+^/Cd^2+^, Mg^2+^, and CaPif1. Furthermore, regardless of whether Zn^2+^, Ni^2+^, or Cd^2+^ was added to reaction system first, or Mg^2+^ was added to reaction system first, or Zn^2+^/Ni^2+^/Cd^2+^ and Mg^2+^ were added at the same time, the inhibitory effects were similar for each heavy metal (Supplementary Table S4.2).

Next, we tested whether EDTA could restore the unwinding activity inhibited by Cd^2+^ and Zn^2+^. After the complete reaction of 1.5 mM Zn^2+^ or Cd^2+^ with 25 nM CaPif1 at 30°C for 5 min, we cleared the metal with 3 mM EDTA (this concentration could completely remove the divalent metals in the mixture but was not sufficient to affect subsequent experiments), and then 1.5 mM Mg^2+^ and 5 mM ATP were added to the mixture to initiate the spin reaction. The Zn^2+^ effect was abolished by adding EDTA (Figure 5C, lane 2 and lane 3), while the Cd^2+^ effect was not counteracted by EDTA (Figure 5C, lane 4 and lane 5). It is possible that Cd^2+^ can damage the active center of CaPif1 more severely at high concentrations (1.5 mM), so that even after removing the ions, the unwinding activity of CaPif1 cannot be restored. Further research showed that lower concentrations of Zn^2+^ and Cd^2+^ significantly inhibited the unwinding activity of CaPif1. When the concentrations of Zn^2+^ and Cd^2+^ were 100 and 10 μM, respectively, almost the same inhibitory effect was observed. Cd^2+^ showed stronger inhibition compared with Zn^2+^, which was at a concentration 10 times lower than that of Zn^2+^ (Figure 5D).

In addition, we sought to examine whether the Zn^2+^ and Cd^2+^ could also inhibit the ATPase and DNA binding activities of CaPif1. Our data showed that whether the binding buffer contained 100 μM of Zn^2+^ (or Cd^2+^) or not, the fluorescence anisotropy values were not significantly different (Figure 5E), indicating that Zn^2+^ and Cd^2+^ had no significant effect on the DNA binding activity of CaPif1. The ATP hydrolysis activity of CaPif1 was a function of ATPase, so the ATP concentration was detected before and after the reaction. The data showed that the ATPase activity containing Zn^2+^ and Cd^2+^ was significantly lower than that without Zn^2+^ and Cd^2+^ (Figures 5F,G). Further analysis revealed that when the concentration of Cd^2+^ reached 100 μM, the ATPase activity of CaPif1 was almost completely inhibited. Thus, we speculate that heavy metals inhibit the unwinding activity of CaPif1 by blocking its energy pathway.

### The Effects of Amino Acids and ATP on the Zn^2+^ (Cd^2+^) inhibition

Although it was difficult to recover the effect of high concentration of Cd^2+^ on CaPif1, we found that when Cd^2+^ (10 µM) and Zn^2+^ (70 µM) were at low concentrations (significantly inhibiting the unwinding of CaPif1), Cys and ATP could reduce this damage. Previous studies have reported that free sulfhydryl groups of Cys are good candidates for Cd^2+^ binding (Witkiewicz-Kucharczyk and Bal, 2006).

**FIGURE 6:**
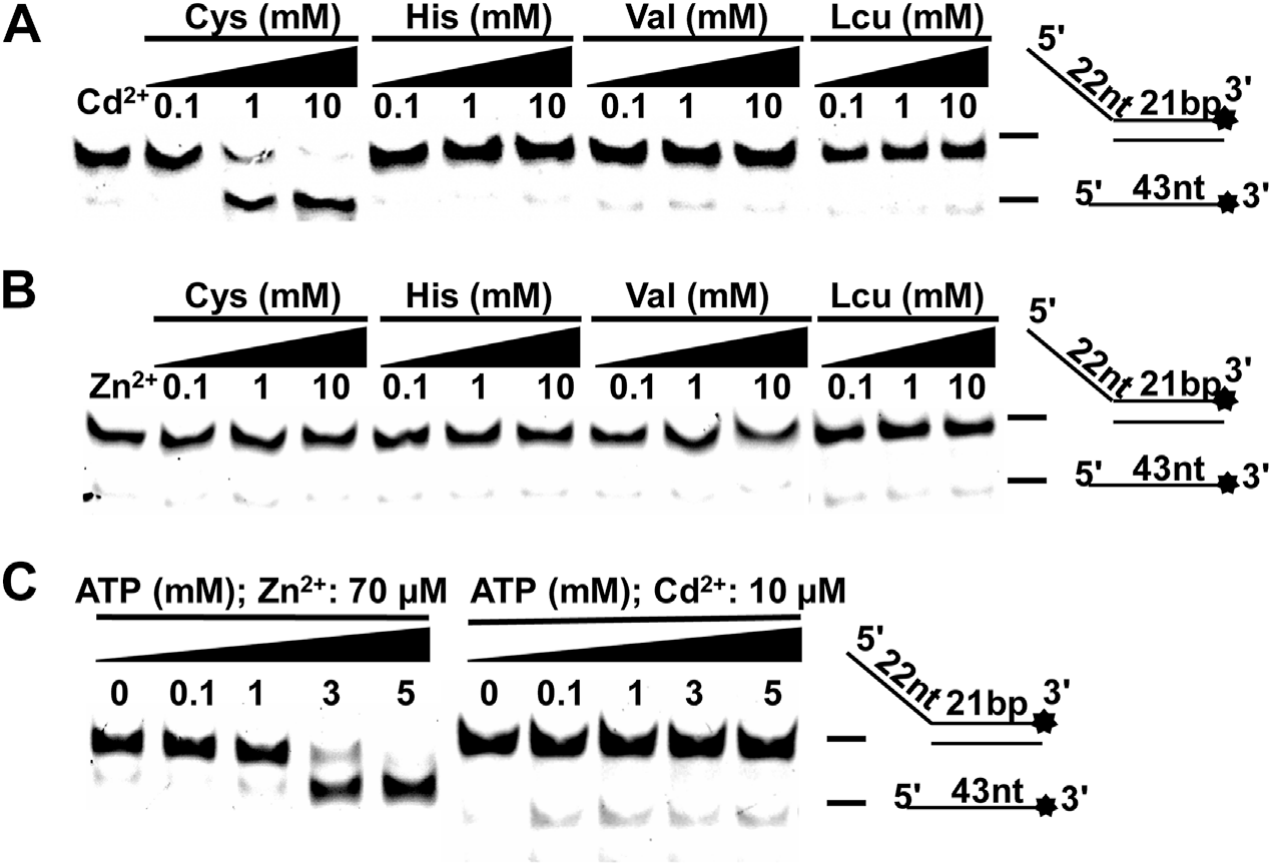
The effects of amino acids and ATP on the Zn^2+^ (Cd^2+^) inhibition. **(A–B)** CaPif1 (25 nM) was reacted with the DNA OhS22D21 (10 nM) in the presence of the indicated amino acids. Lane 1: The control group containing Cd^2+^ (10 μM) or Zn^2+^ (70 μM) without amino acid; 0.1, 1, and 10 represent the amino acid concentration (mM). **(C)** In the reaction of lower concentrations of Cd^2+^ (10 µM) or Zn^2+^ (70 µM), the concentration of was increased ATP.

At the same time, Cys, histidine (His), valine (Val), and leucine (Leu) were also included to check their interaction with Cd^2+^/Zn^2+^. They were incubated with unwinding buffer containing Cd^2+^ at 30°C for 5 min. Then CaPif1 was added and incubated at 30°C for another 5 min, before ATP was added to start the reaction. We found that with increasing Cys concentrations, the inhibition of the unwinding activity of CaPif1 by Cd^2+^ decreased significantly. This may have been due to Cys binding to the Cd^2+^ in the reaction, so the concentration of free Cd^2+^ was low, which reduced the damage to the activity of CaPif1 (Figure 6A). None of the four amino acids significantly alleviated damage in samples containing Zn^2+^ (Figure 6B).

We changed the order that samples were added in. For example, CaPif1 was first pre-incubated with the solution containing 10 µM Cd^2+^, and then 10 mM Cys was added. This process still showed a reduction of the inhibition of CaPif1’s unwinding activity by Cd^2+^ (Supplementary Table S5). This may imply that during the unwinding process of CaPif1, increasing the concentration of Cys to a certain level can prevent the inhibitory effect of Cd^2+^.

Finally, we increased the concentration of ATP to alleviate the inhibition of CaPif1’s unwinding activity by low concentrations of Zn^2+^ and Cd^2+^. The two experiments showed different results. Increasing ATP concentration obviously reduced the inhibition of Zn^2+^, while Cd^2+^ did not resume the unwinding activity of CaPif1 to an obvious degree (Figure 6C). The possible reason may be that the CaPif1 unwinding activity is ATP-dependent. Therefore, increasing the ATP concentration within a certain range can increase the activity of CaPif1, showing an apparent recovery of CaPif1 activity. The damage caused by Cd^2+^ is more serious than that caused by Zn^2+^, so it may not be able to offset this damage through the increase of ATP.

### The Effects of DTT on CaPif1 Unwinding Activity inhibited by Zn^2+^ and Cd^2+^

**FIGURE 7:**
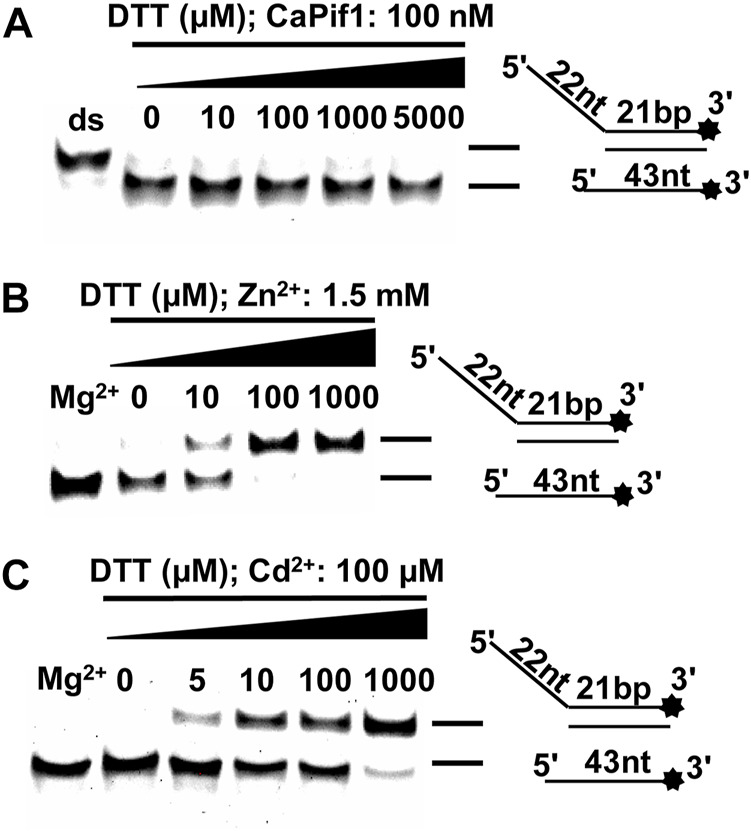
The effects of DTT on CaPif1 unwinding activity inhibited by Zn^2+^ and Cd^2+^. **(A)** The unwinding activity of CaPif1 at different concentrations of DTT, containing only MgCl_2_, no other divalent metal ions. **(B–C)** The unwinding activity of CaPif1 at 1.5 mM Zn^2+^ or 100 μM Cd^2+^ and different concentrations of DTT. Lane Mg^2+^: A standard unwinding experiment containing MgCl_2_ without Zn^2+^ or Cd^2+^.

DTT has been previously described having Cd^2+^-induced cell damage and enzyme inhibition (Stacey, 1986; Ahammadsahib et al., 1989). All the results mentioned above were obtained under reducing conditions (in the presence of DTT). We first fixed the concentration of CaPif1 in the reaction without Zn^2+^ and Cd^2+^, and increased the concentration of DTT. The results showed that DTT had no effect on the unwinding activity of CaPif1 (Figure 7A). Then we compared the unwinding activity of CaPif1 in the presence of 1.5 mM Zn^2+^ or 100 μM Cd^2+^ and gradually increased the concentration of DTT. In the reaction without DTT, neither Zn^2+^ nor Cd^2+^ could inhibit the unwinding activity of CaPif1 (Figures 7B,C). This implied that CaPif1 resisted the inhibitory effect of Zn^2+^ and Cd^2+^ in less reducing environments. We speculate that when there is a certain concentration of DTT in the environment, the disulfide bond of the protein is opened, thereby releasing more sulfhydryl groups on the surface of the protein, and Zn^2+^/Cd^2+^ can bind tightly to the sulfhydryl group of cysteine, resulting in more heavy metals destroying the activity of the protein. Interestingly, the reducing status in the body will decrease in the presence of Cd^2+^, which stimulates ROS production in the body (Srinivas et al., 2019). We speculate that CaPif1 has formed a mechanism to respond to heavy metal poisoning by effectively sensing the reducing status changes.

## Discussion

Cadmium is a recognized human and animal carcinogen. It can cause DNA damage directly by inhibiting the activity of DNA-repair proteins and stimulating ROS production, thus reducing the status of the body (Hengstler et al., 2003). Zinc and cadmium have similar physical and chemical properties. Both are closely related to DNA-repair proteins. Pif1, a DNA-repair protein, acts at telomeres, rDNA, G-quadruplexes, R-loops, break-induced replication, and Okazaki fragment maturation (Garcia-Rodriguez et al., 2018). In this context, the study of heavy metal toxicity on Pif1 is of great significance to understand the mechanism of heavy metal toxicity and the role of Pif1 in maintaining genomic stability.

First, we reported the expression, purification, and biochemical analysis of CaPif1. It was confirmed that it had DNA binding, annealing, and ATP- and Mg^2+^-dependent 5′→3′ direction helicase activity. CaPif1 can utilize the five types of NTP or dNTP, showing a preference for ATP and CTP; even a high concentration (5 mM) of GTP, UTP, or dTTP can inhibit the unwinding activity. Pif1 has been reported to efficiently unwind various structured DNA preferences to duplex DNA with 5′-overhangs (Liu et al., 2015). CaPif1 prefers to unwind 5′-overhang DNA with long 5′-tail strands and a short duplex stranded region, indicating that the unwinding activity of CaPif1 has a certain persistence.

Second, the effects of six heavy metals (Mn, Ca, Zn, Ni, Cu, and Cd) on the unwinding activity of CaPif1 were detected. The presence of Mn^2+^ or Ca^2+^ can also replace Mg^2+^ and support the CaPif1-driven unwinding of 5′-overhang DNA. Notably, Zn^2+^ and Cd^2+^ cannot support it and inhibit the unwinding activity and ATPase of CaPif1 but have no effects on DNA binding activity. The inhibitory effect of Zn^2+^ at 100 μM (Cd^2+^ at 10 μM) was more than 70%, and Cd^2+^ was 10 times stronger than Zn^2+^. Free cysteine protects the unwinding activity of the enzyme from Cd^2+^, indicating that Cd^2+^-dependent unwinding activity effect may be associated with the cysteine residues of CaPif1. EDTA could not reverse high concentration of Cd^2+^ inhibition, which suggests that high concentration of Cd^2+^ causes irreversible changes in the protein structure. In contrast, the inhibition of Zn^2+^ can be reversed by EDTA and can be mitigated by adding ATP, thus showing that Cd^2+^ is more destructive. Zn^2+^ has been previously reported to inhibit cadmium toxicity (Qin et al., 2016). This is similar to our experimental results. With the same inhibitory effect, the concentration of Zn^2+^ is nearly 10 times higher than that of Cd^2+^.

Finally, we found an interesting phenomenon: Cd^2+^ and Zn^2+^ could not significantly inhibit the unwinding activity of CaPif1 under non-reducing conditions, and the inhibition was proportional to the degree of reduction. Since it was previously shown that the reducing environment of the body decrease in the presence of Cd^2+^, as the metal stimulated an increase of ROS in the body, CaPif1 may resist cadmium poisoning by monitoring the reducing status of the body. This may be an ancient mechanism for organisms to adapt to heavy metal stress.

Collectively, we report the functions of CaPif1 and the toxicity mechanism of Zn^2+^ and Cd^2+^ to CaPif1. This work provides new clues to help us better understand the toxicological mechanism of heavy metal toxicity.

## Data Availability

The original contributions presented in the study are included in the article/Supplementary Material, further inquiries can be directed to the corresponding authors.
