# Supplementary material for: Mechanistic Insight Into Cadmium- and Zinc-Induced Inactivation of the Candida albicans Pif1 Helicase
Source: Front Mol Biosci. 2022 Jan 21;8:778647. doi: 10.3389/fmolb.2021.778647 (PMC8815974; doi:10.3389/fmolb.2021.778647)
Supplement: Supplementary file 1 [file DataSheet3.ZIP › Supplement 3.docx]

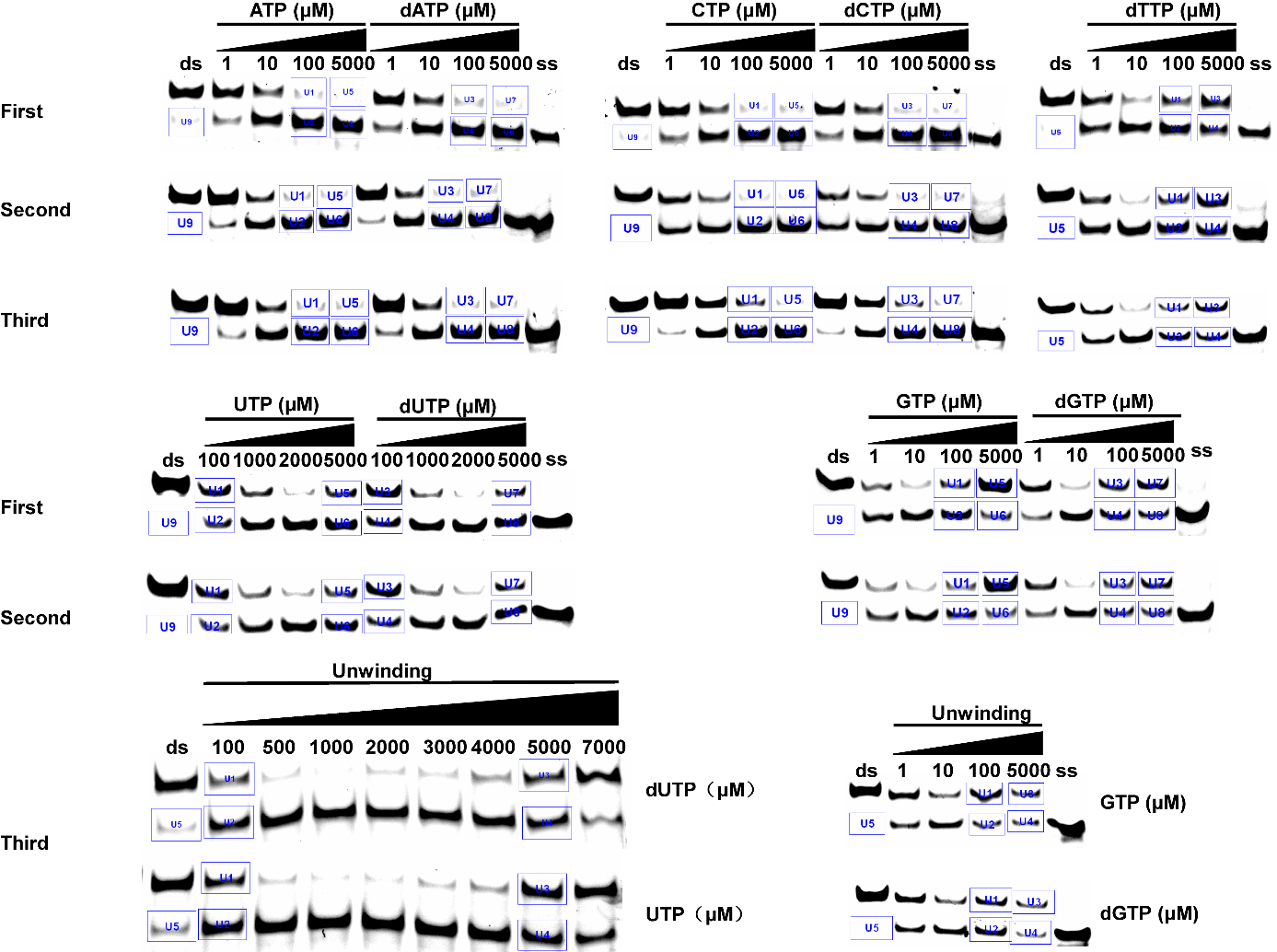


**Supplement 3.** DNA was quantitated as shown above by using the Image Lab software (Bio-Rad) to get the adjusted volume (See file“Supplement 3, Grey value.xlsx”for details), and using it to calculate the fraction using the following formula: $\%unwinding=100\times\frac{P}{S+P}$, where P is the product and S is the substrate. Take ATP as an example： $\% unwinding=100\times\frac{U2－U9}{U1＋U2－U9}$.where U2 is the product, U1 is the substrate, U10 is the spontaneously unwind product, U2-U10 is the CaPif1 unwind product.

**Supplement 3, table.** The original data of the unwinding ratio

| **Unwinding (%)** | **100μM** | **Stdev** | **5000μM** | **Stdev** |
| --- | --- | --- | --- | --- |
| **ATP** | 82.31939 | 0.8733 | 86.43191 | 3.27329 |
| **d ATP** | 82.86804 | 1.99388 | 85.02742 | 1.02705 |
| **CTP** | 80.20047 | 6.95228 | 90.94713 | 4.4759 |
| **d CTP** | 79.13833 | 6.81434 | 89.34732 | 5.23081 |
| **GTP** | 47.91723 | 13.69129 | 27.51965 | 3.08198 |
| **d GTP** | 49.74043 | 2.08734 | 37.39483 | 7.57920 |
| **d TTP** | 58.07861 | 1.40009 | 47.95039 | 2.32311 |
| **UTP** | 43.16939 | 8.3201 | 55.17961 | 6.48931 |
| **d UTP** | 42.71477 | 8.13689 | 58.41231 | 2.55509 |
