# Supplementary figures and images for: Mechanistic Insight Into Cadmium- and Zinc-Induced Inactivation of the Candida albicans Pif1 Helicase
Source: Front Mol Biosci. 2022 Jan 21;8:778647. doi: 10.3389/fmolb.2021.778647 (PMC8815974; doi:10.3389/fmolb.2021.778647)

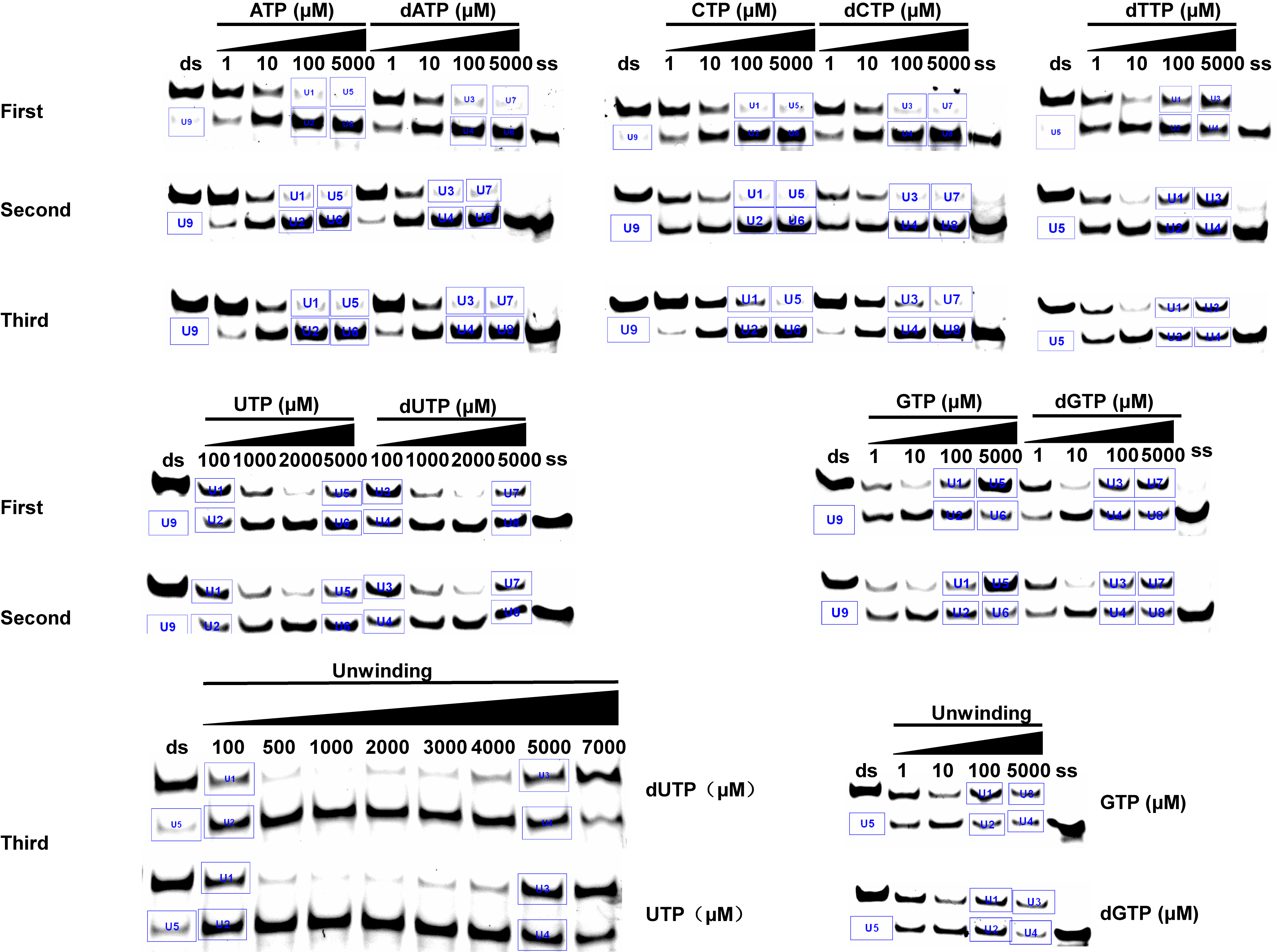

Supplement: Supplementary file 1 [file DataSheet3.ZIP › Supplement 3.tif]

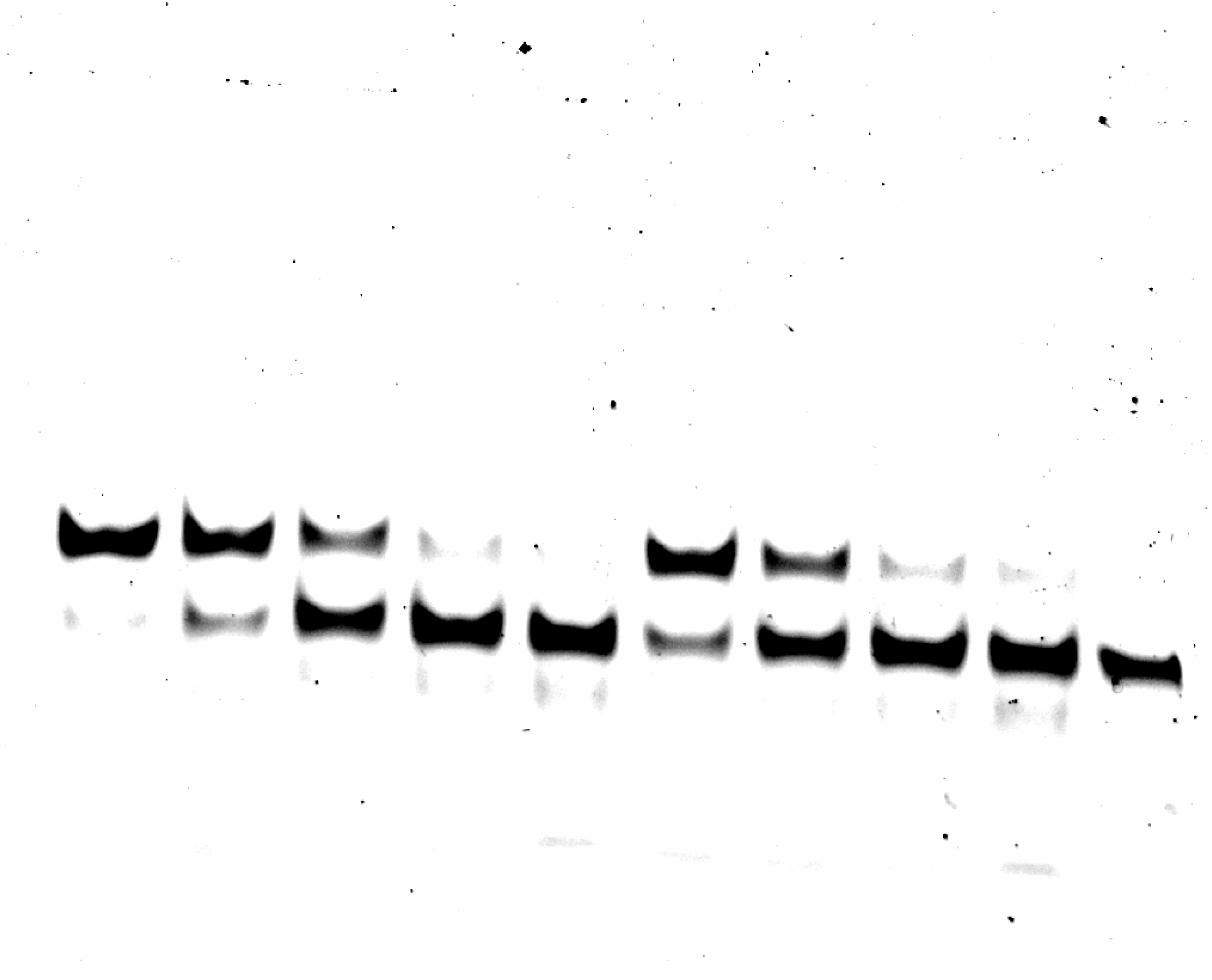

Supplement: Supplementary file 1 [file DataSheet3.ZIP › The original image/ATP dATP-First, the original image.tif]

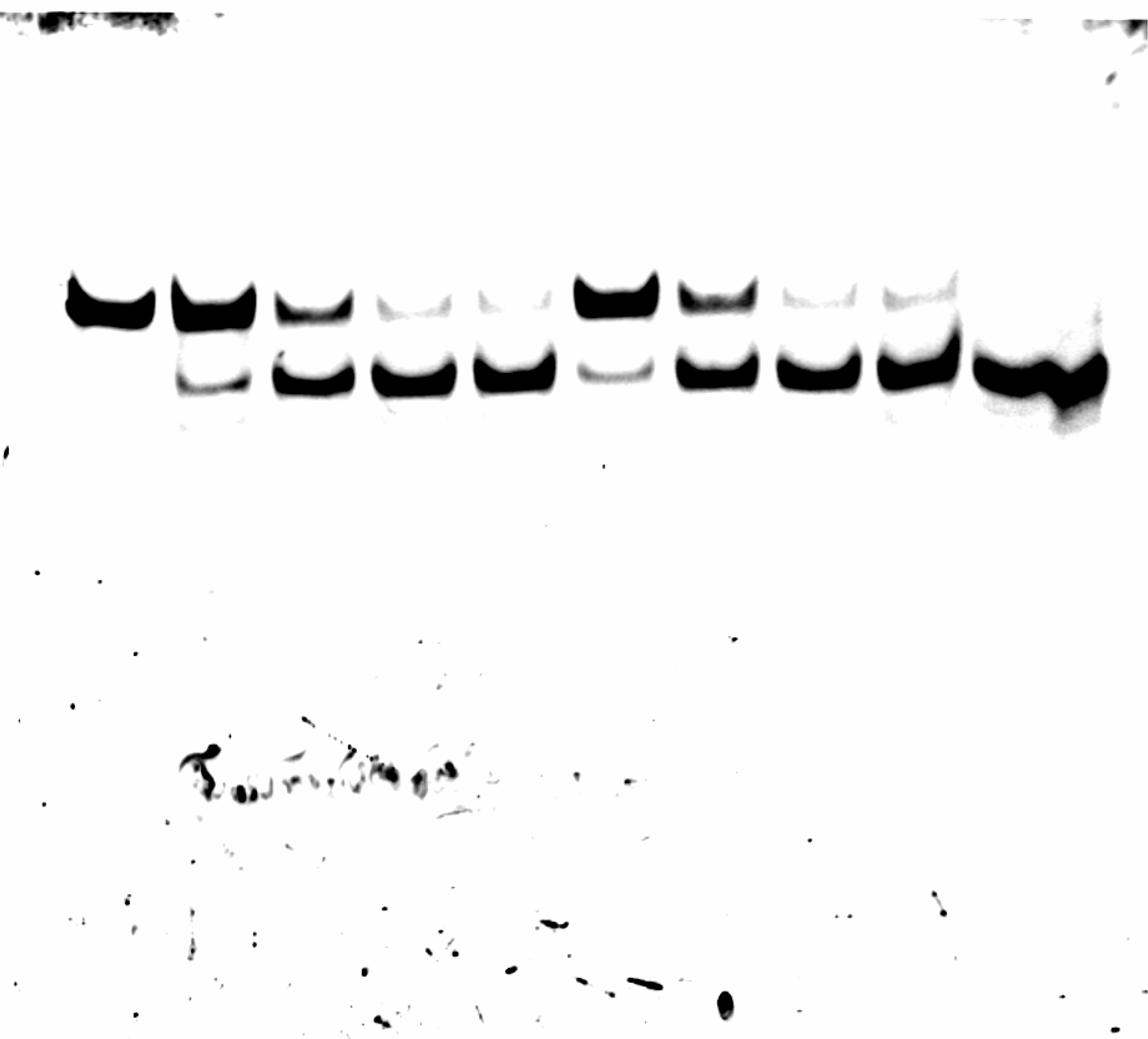

Supplement: Supplementary file 1 [file DataSheet3.ZIP › The original image/ATP dATP-Second, the original image.tif]

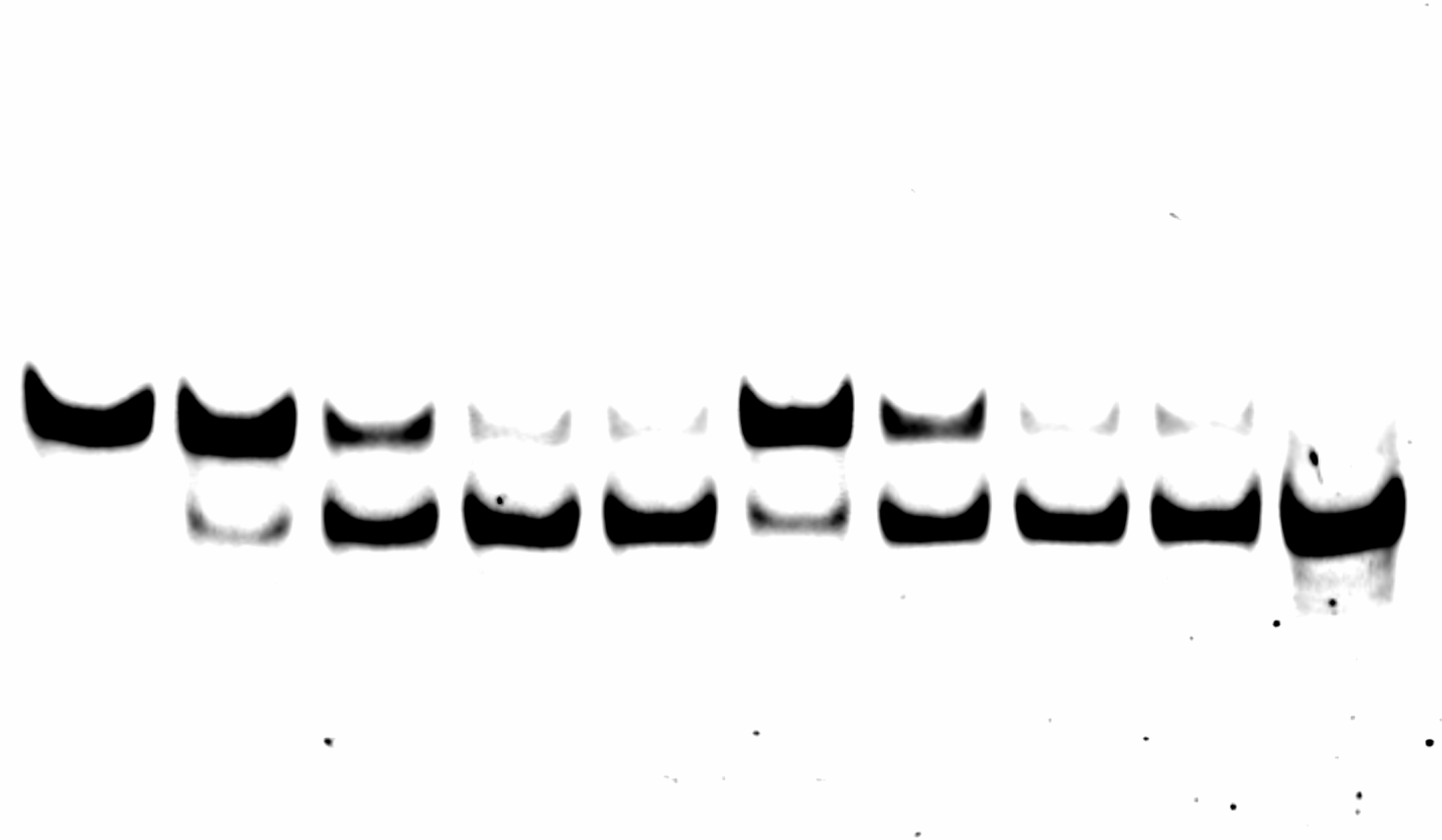

Supplement: Supplementary file 1 [file DataSheet3.ZIP › The original image/ATP dATP-Third, the original image.tif]

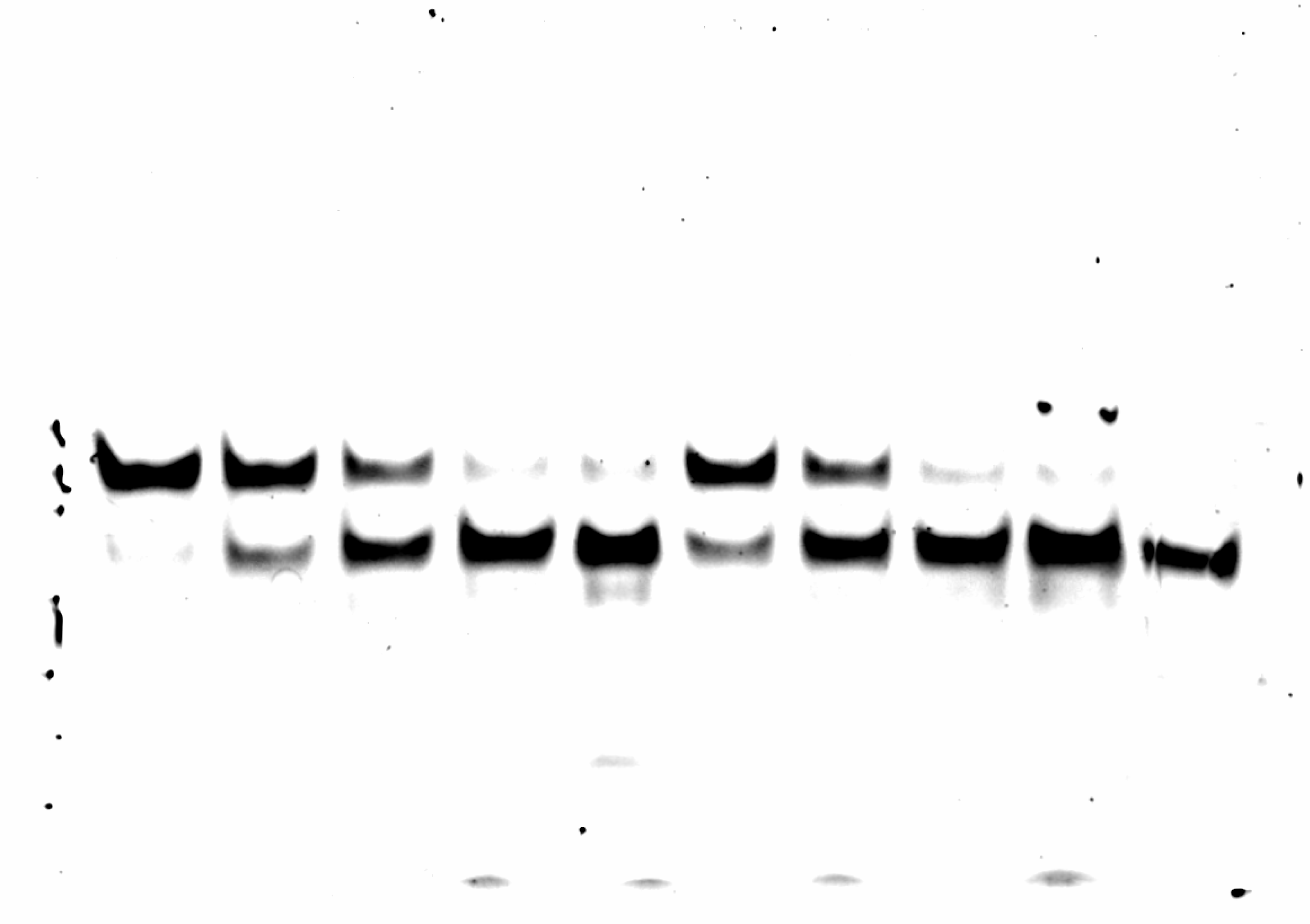

Supplement: Supplementary file 1 [file DataSheet3.ZIP › The original image/CTP dCTP-First, the original image.tif]

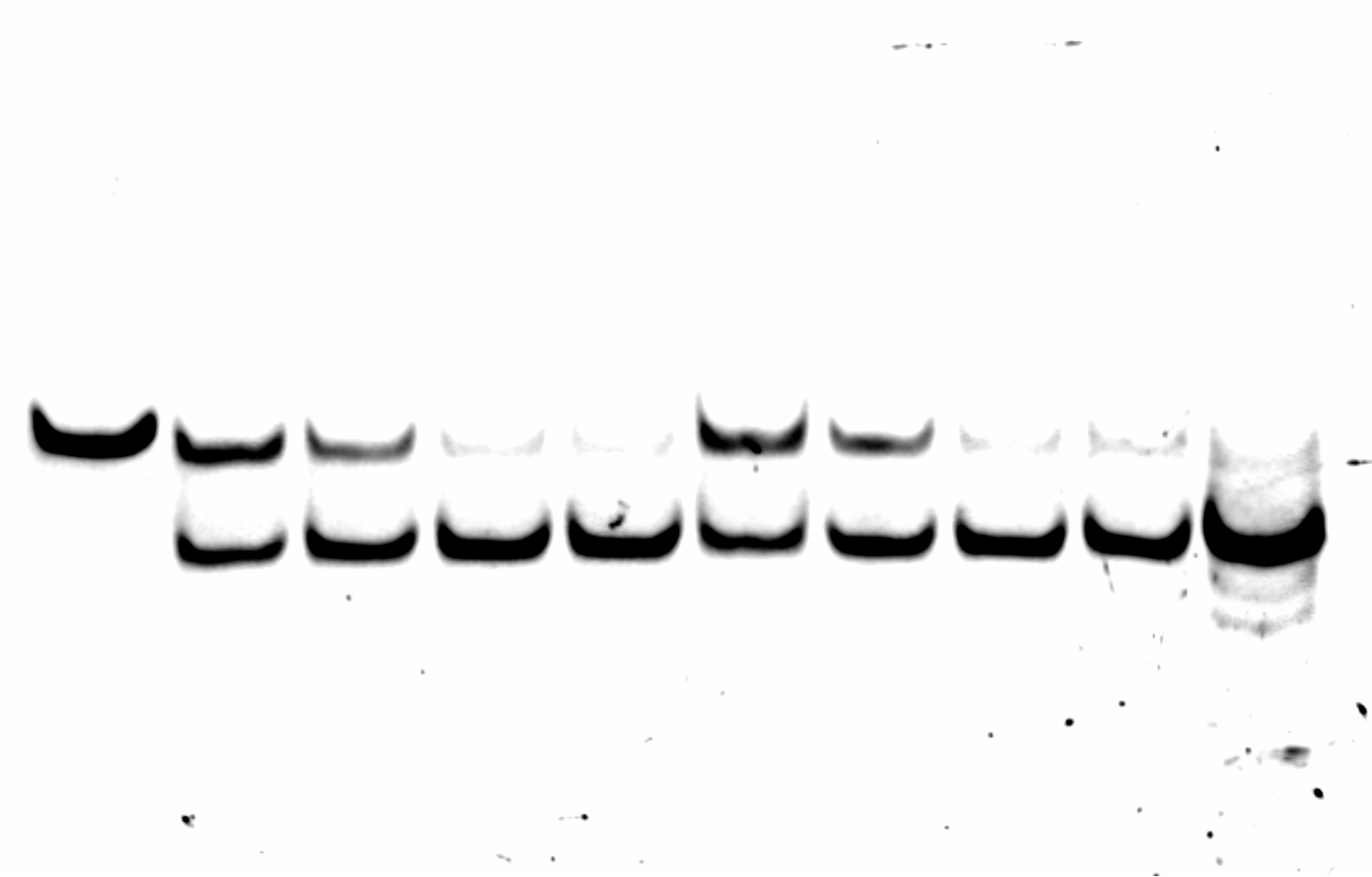

Supplement: Supplementary file 1 [file DataSheet3.ZIP › The original image/CTP dCTP-Second, the original image.tif]

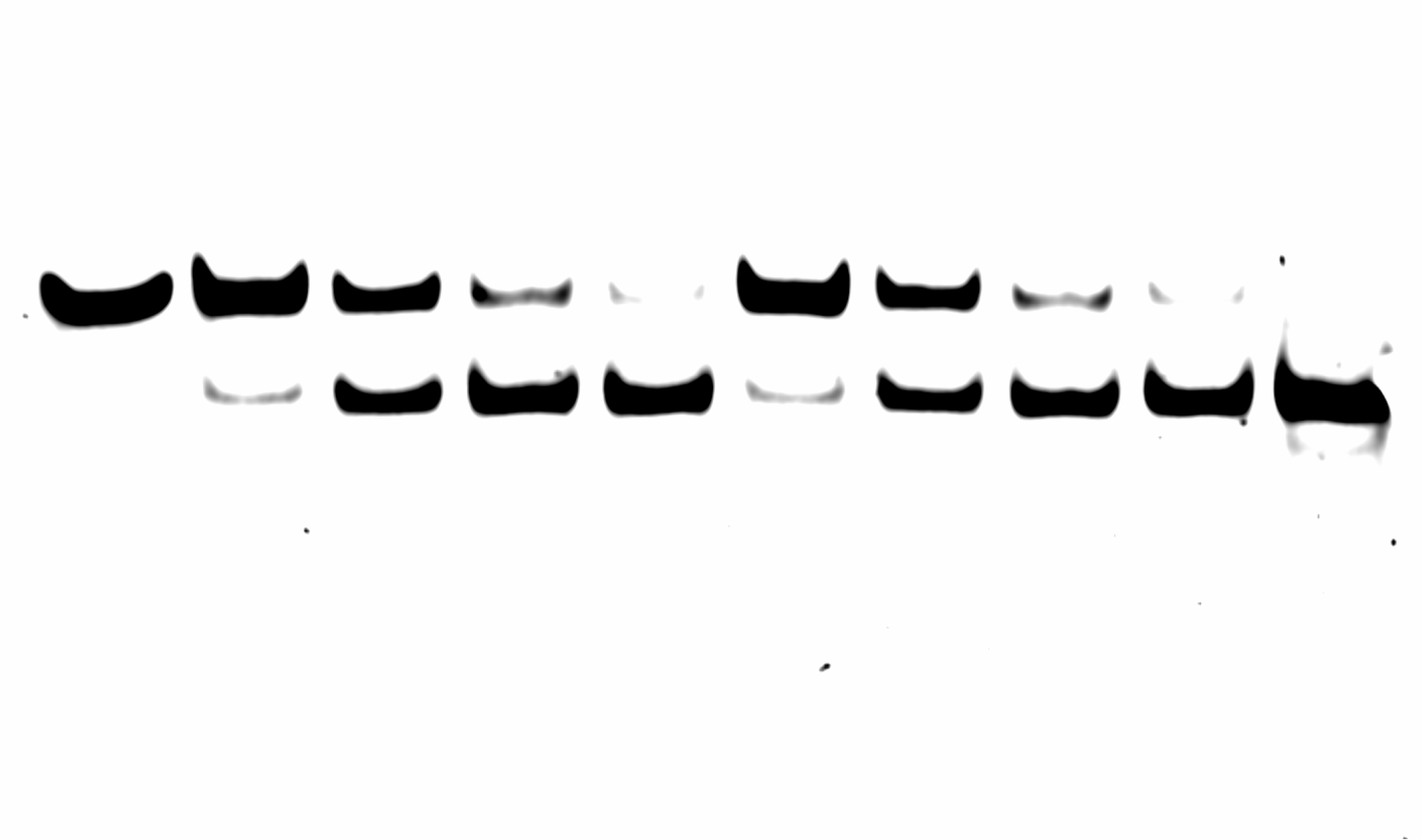

Supplement: Supplementary file 1 [file DataSheet3.ZIP › The original image/CTP dCTP-Third, the original image.tif]

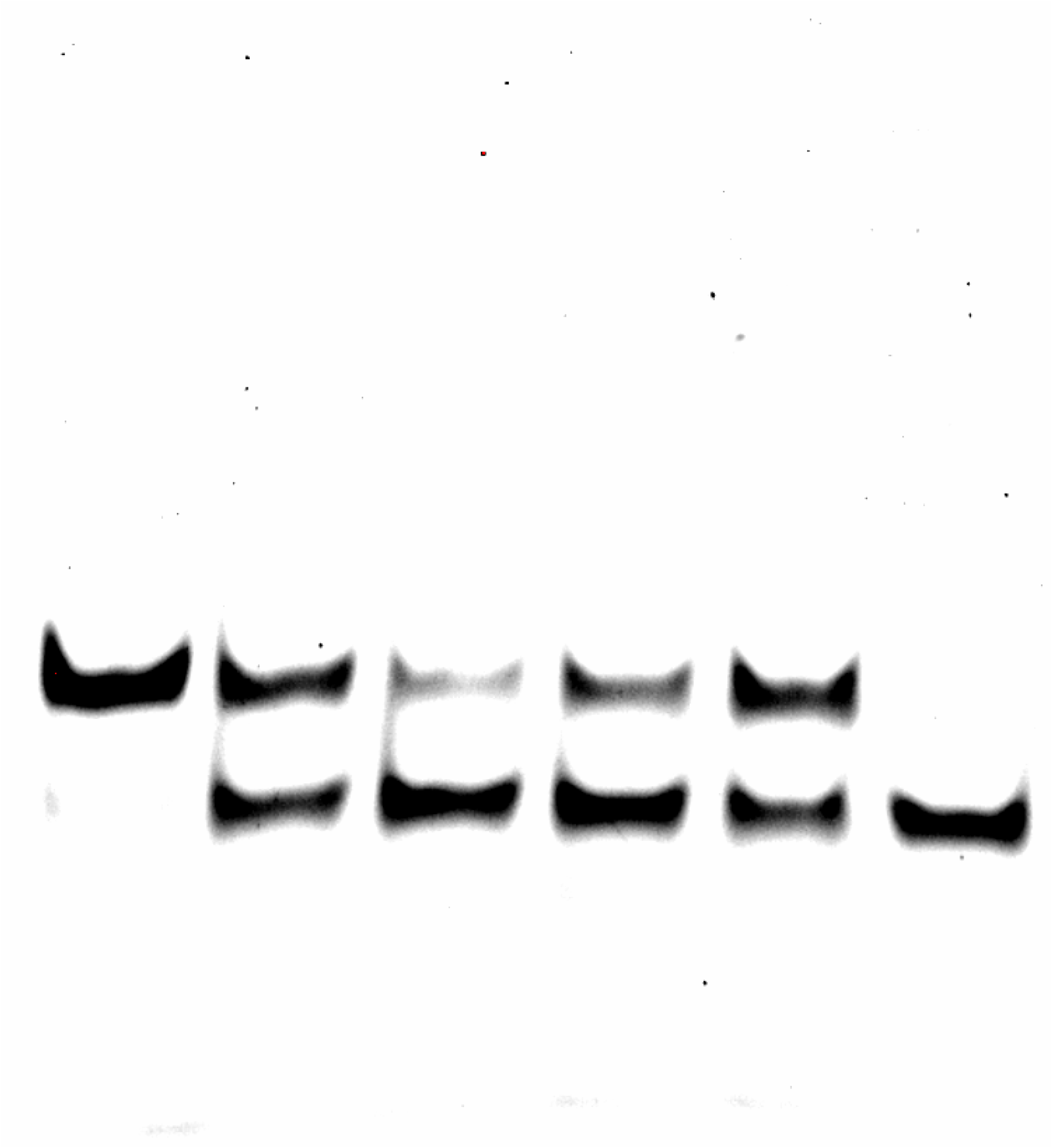

Supplement: Supplementary file 1 [file DataSheet3.ZIP › The original image/dTTP-First,the original image.tif]

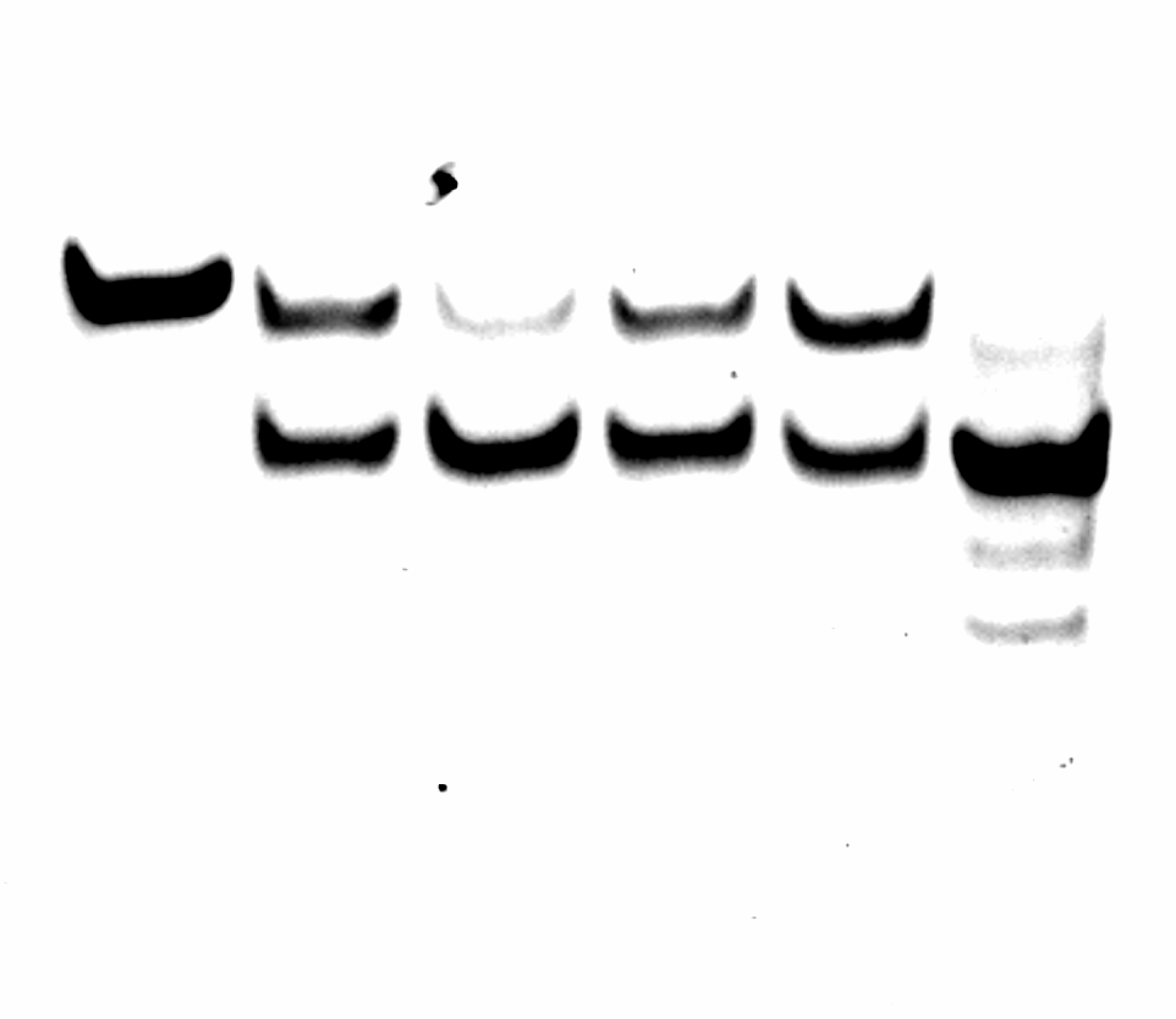

Supplement: Supplementary file 1 [file DataSheet3.ZIP › The original image/dTTP-Second,the original image.tif]

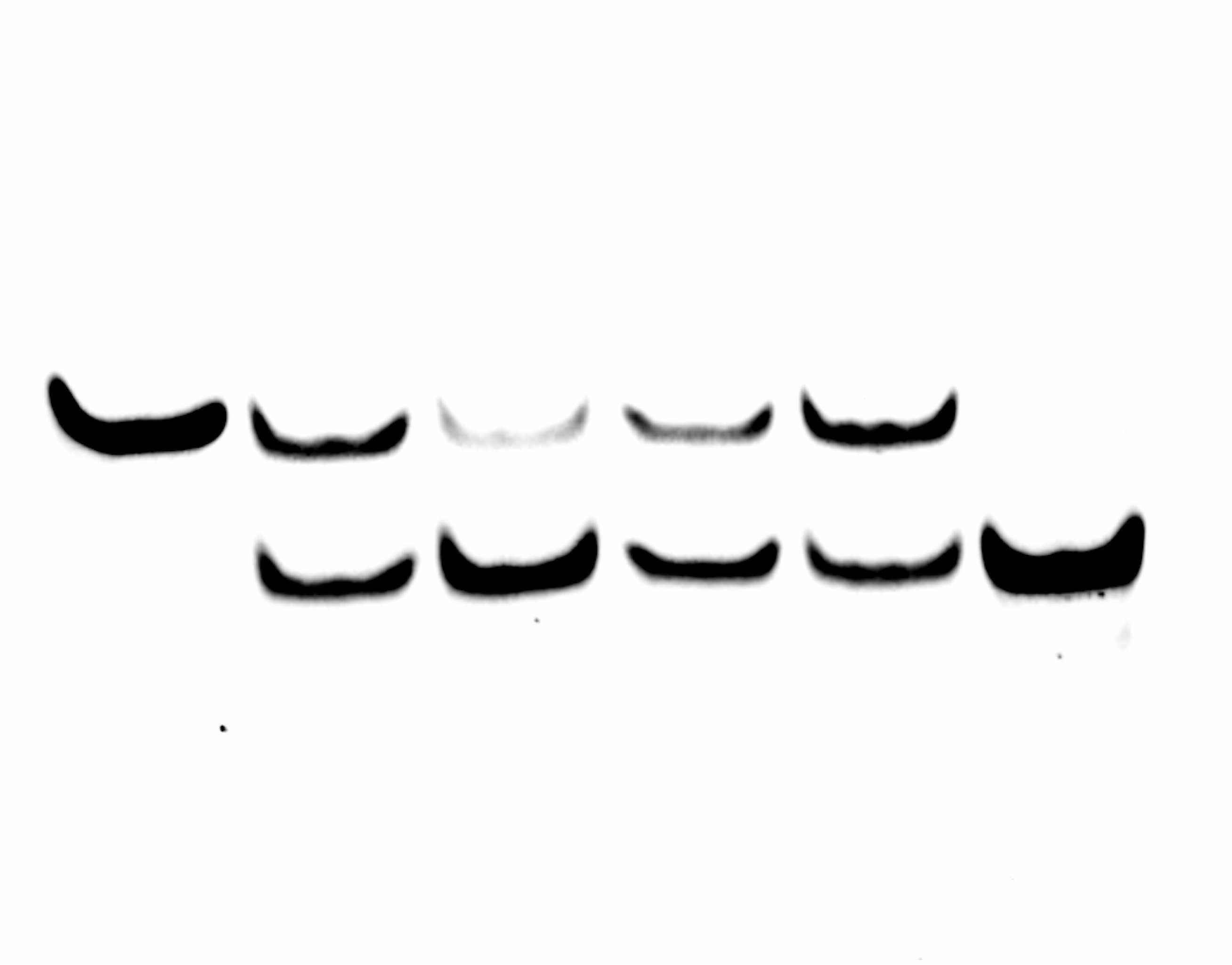

Supplement: Supplementary file 1 [file DataSheet3.ZIP › The original image/dTTP-Third,the original image.tif]

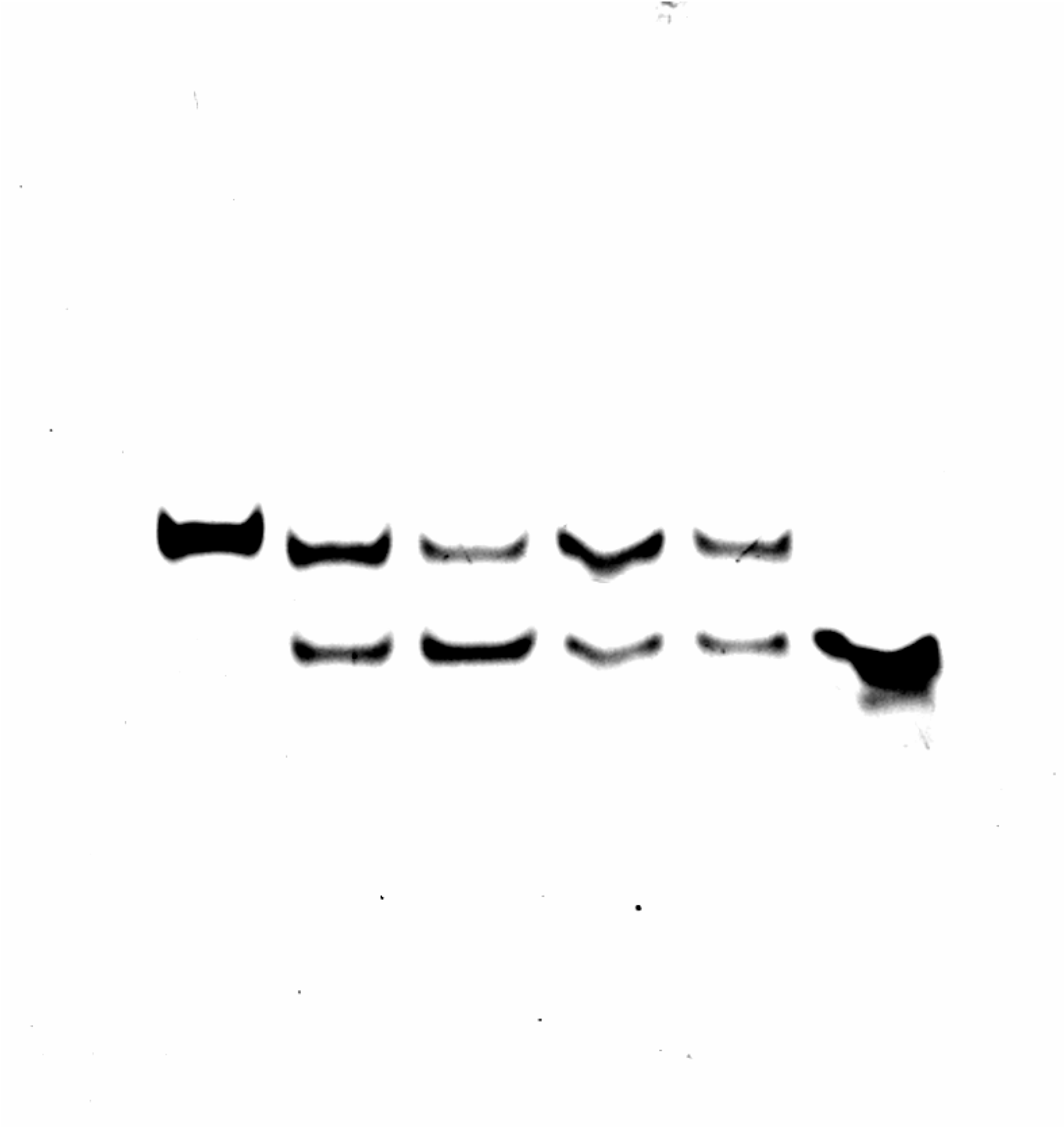

Supplement: Supplementary file 1 [file DataSheet3.ZIP › The original image/GTP -Third, the original image.tif]

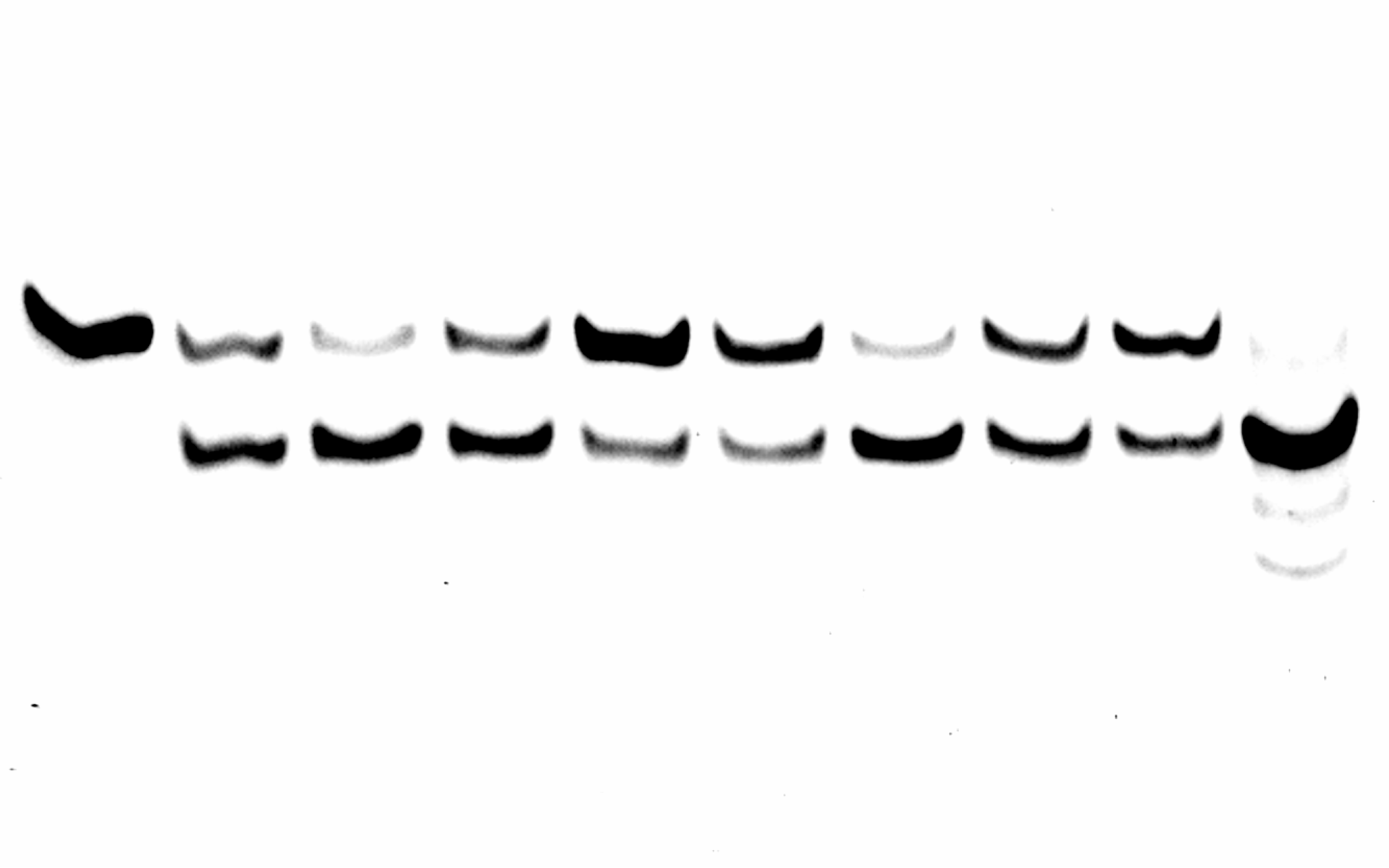

Supplement: Supplementary file 1 [file DataSheet3.ZIP › The original image/GTP dGTP-First, the original image.tif]

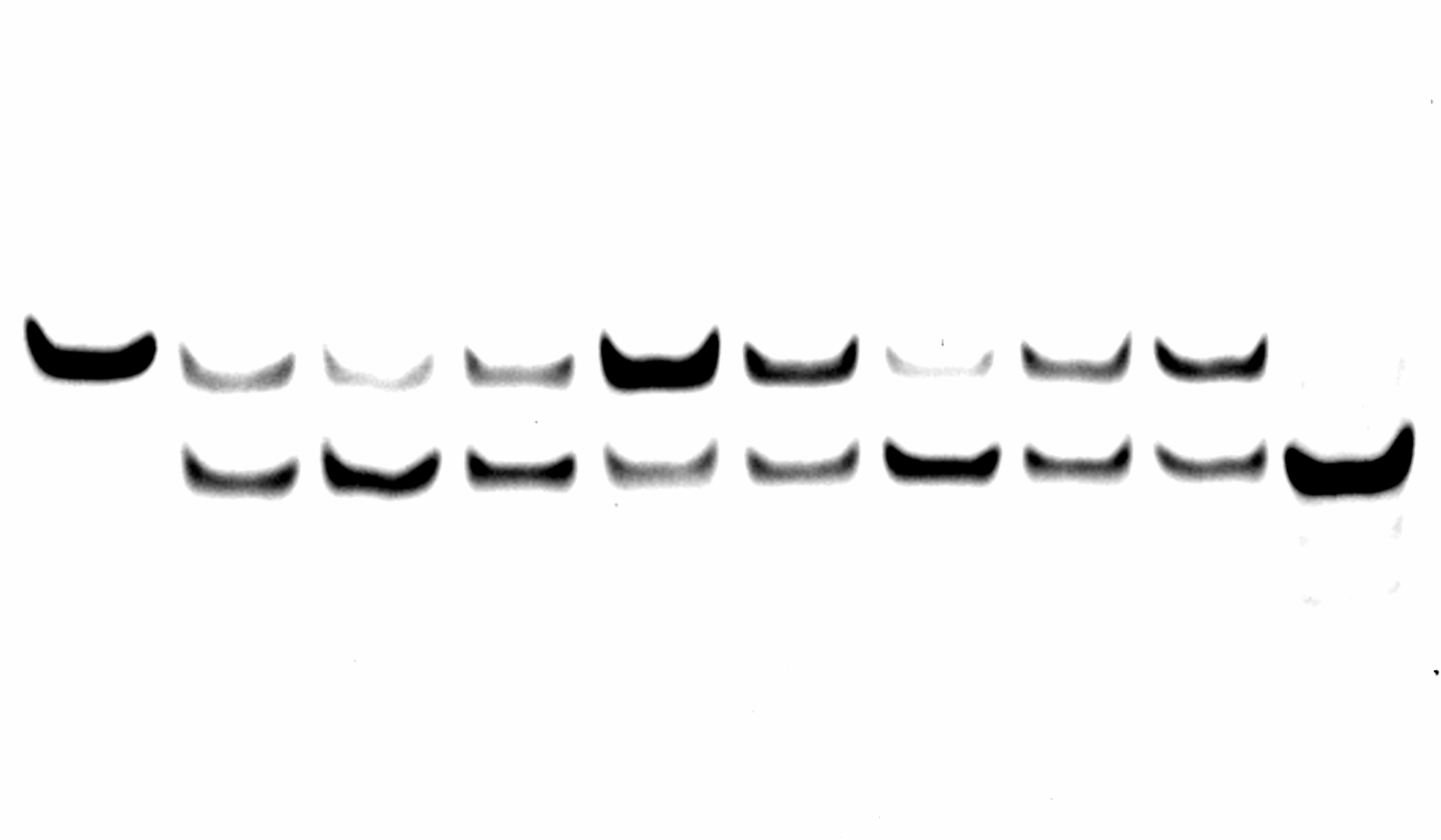

Supplement: Supplementary file 1 [file DataSheet3.ZIP › The original image/GTP dGTP-Second, the original image.tif]

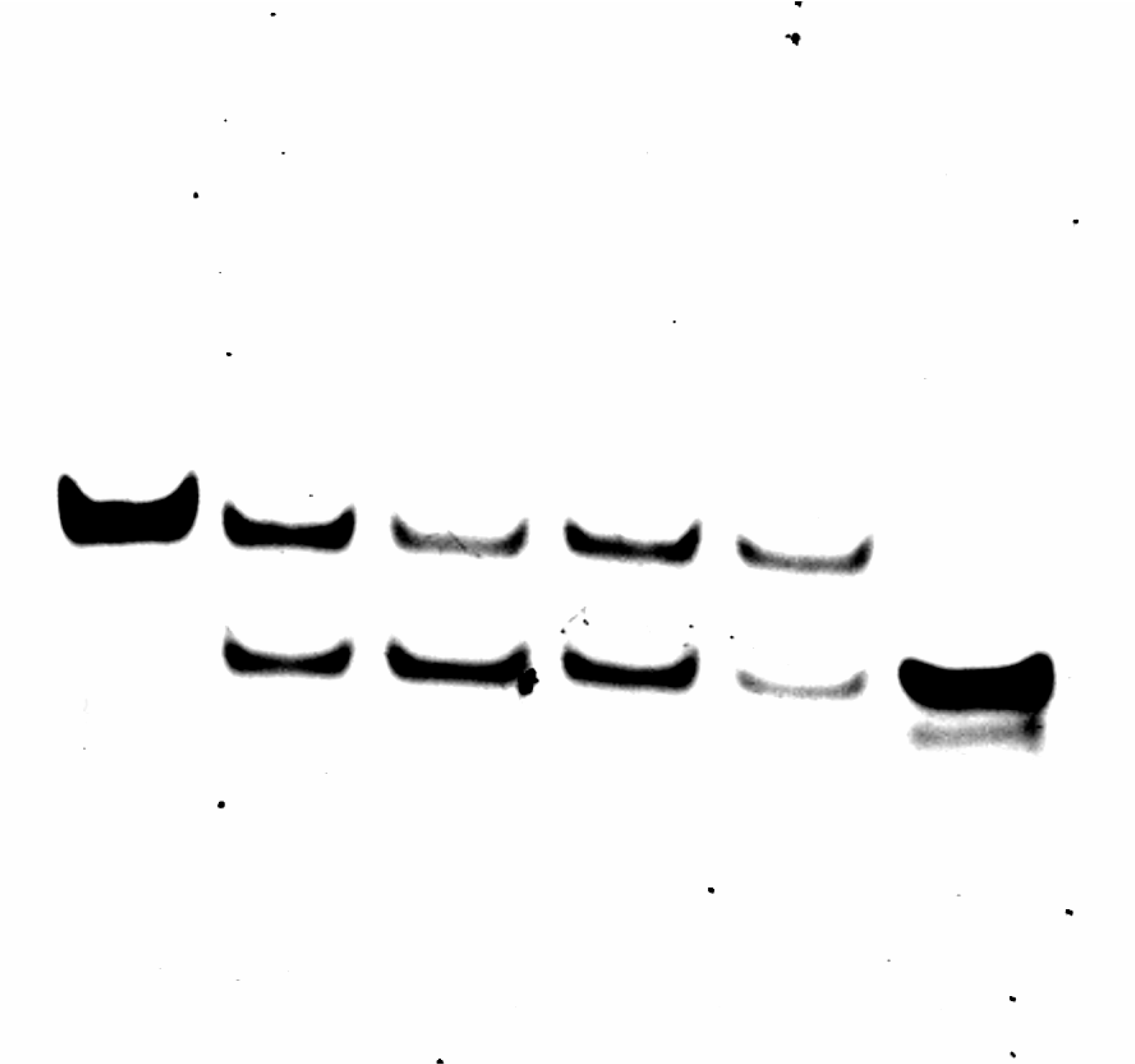

Supplement: Supplementary file 1 [file DataSheet3.ZIP › The original image/GTP(d) -Third, the original image.tif]

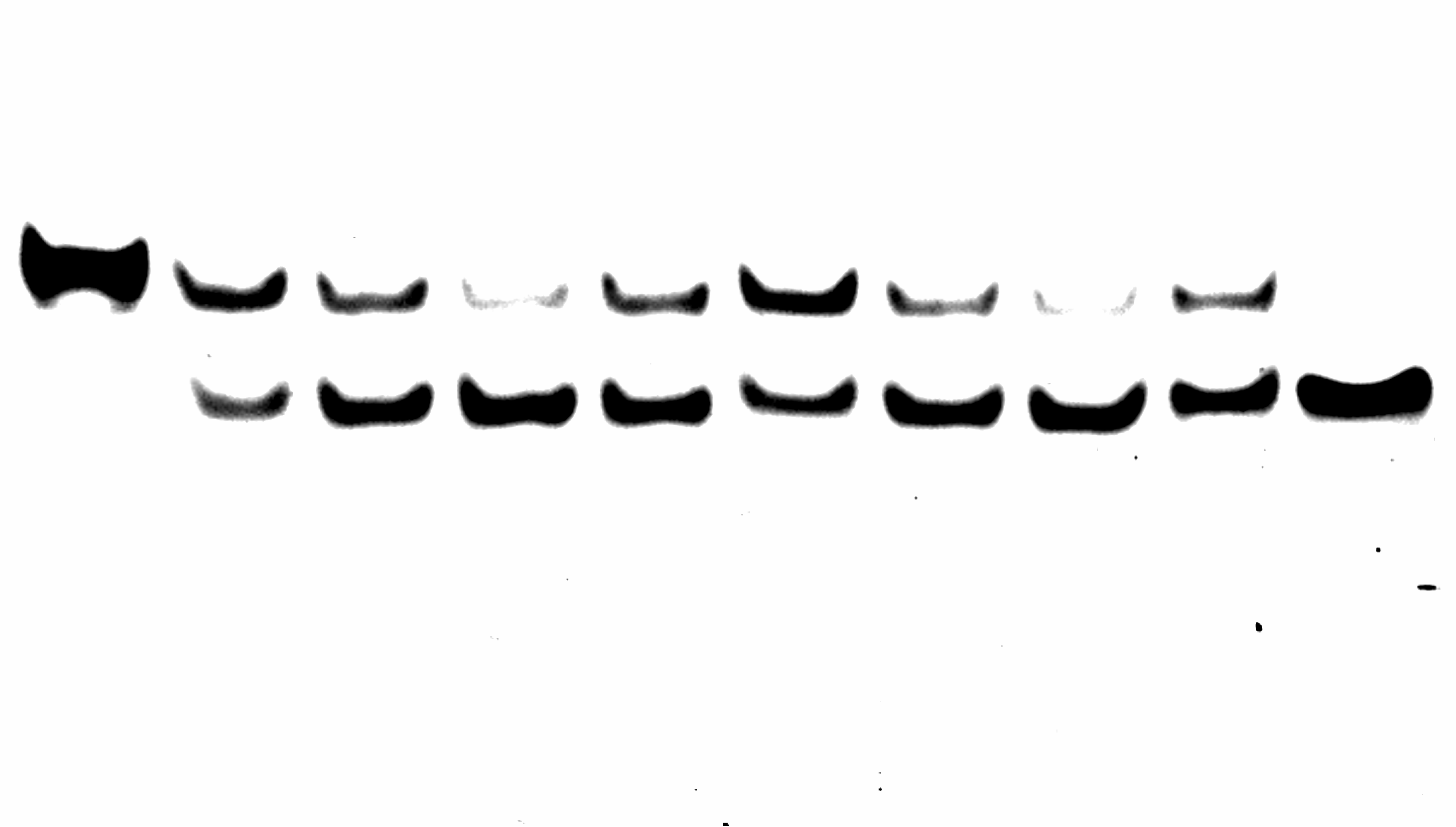

Supplement: Supplementary file 1 [file DataSheet3.ZIP › The original image/UTP dUTP-First, the original image.tif]

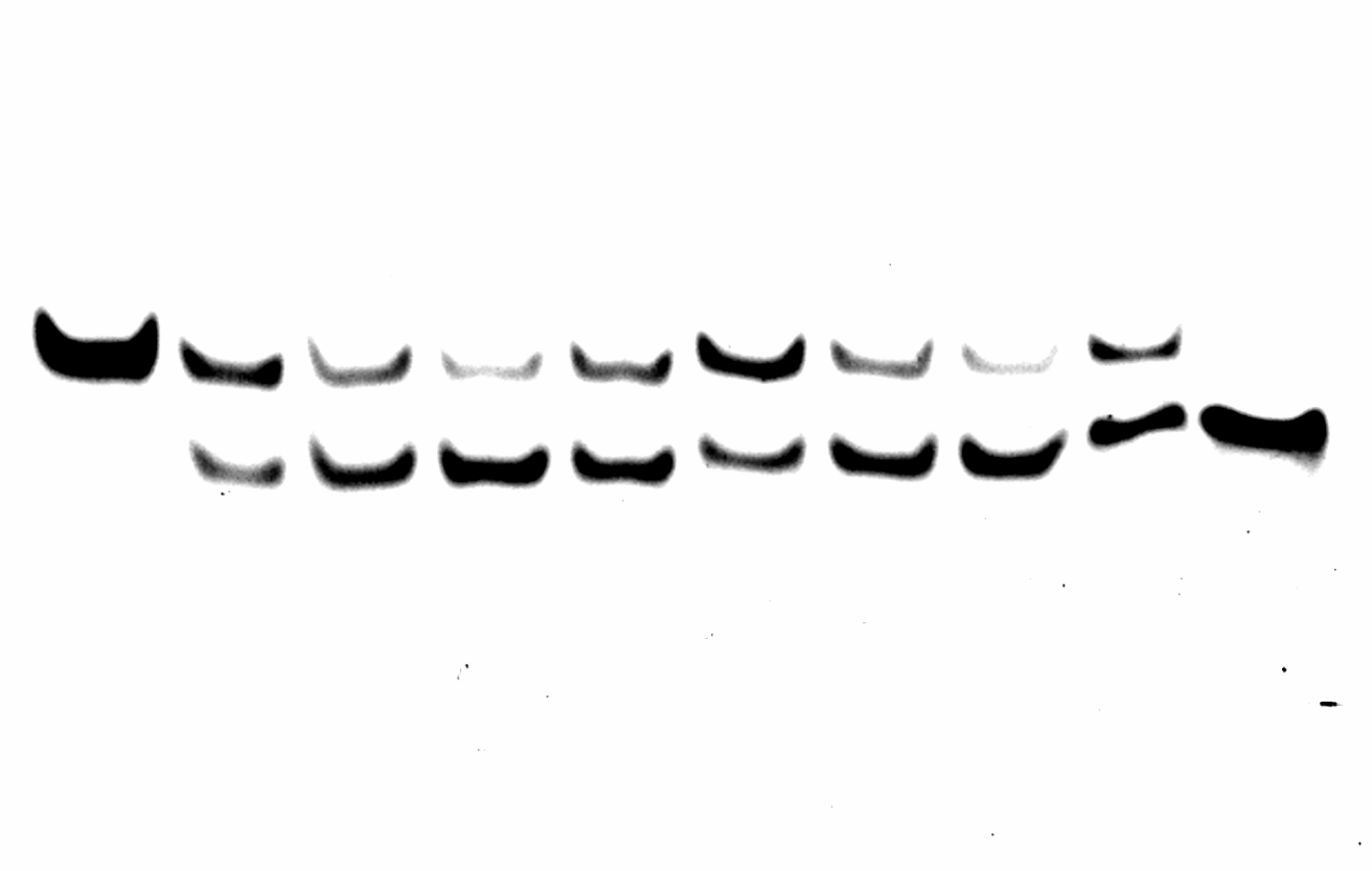

Supplement: Supplementary file 1 [file DataSheet3.ZIP › The original image/UTP dUTP-Second, the original image.tif]

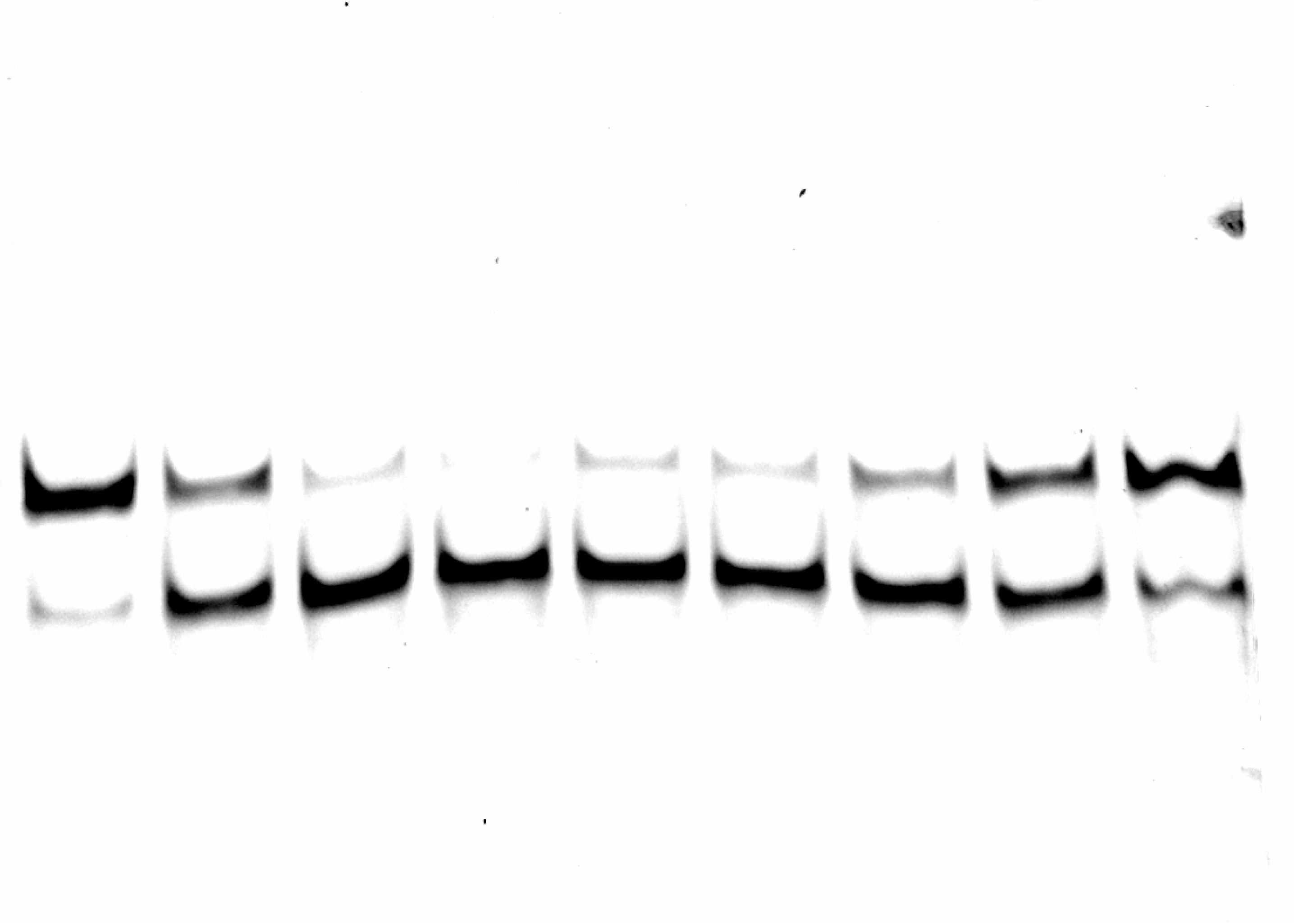

Supplement: Supplementary file 1 [file DataSheet3.ZIP › The original image/UTP(d)-Third, the original image.tif]

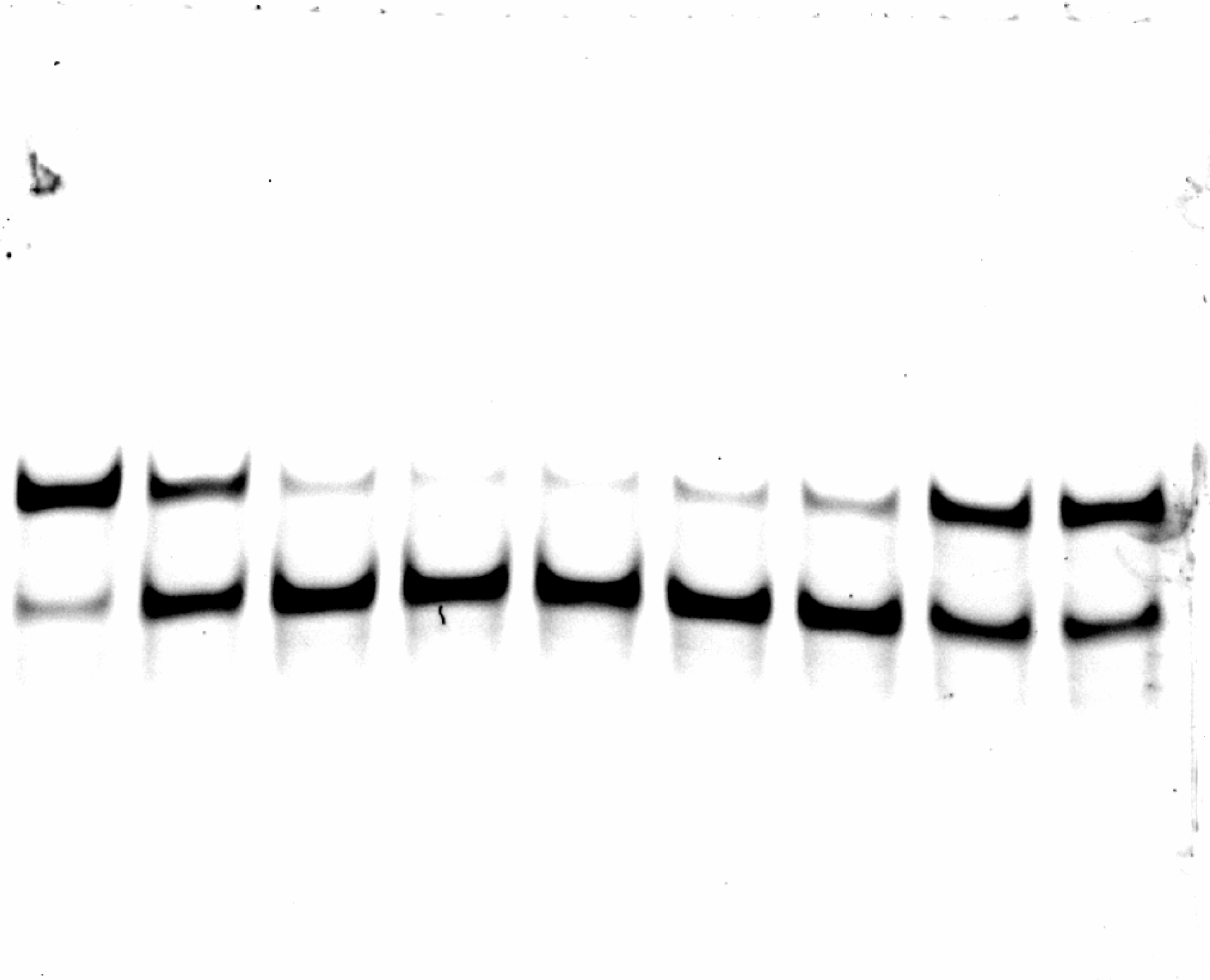

Supplement: Supplementary file 1 [file DataSheet3.ZIP › The original image/UTP-Third, the original image.tif]

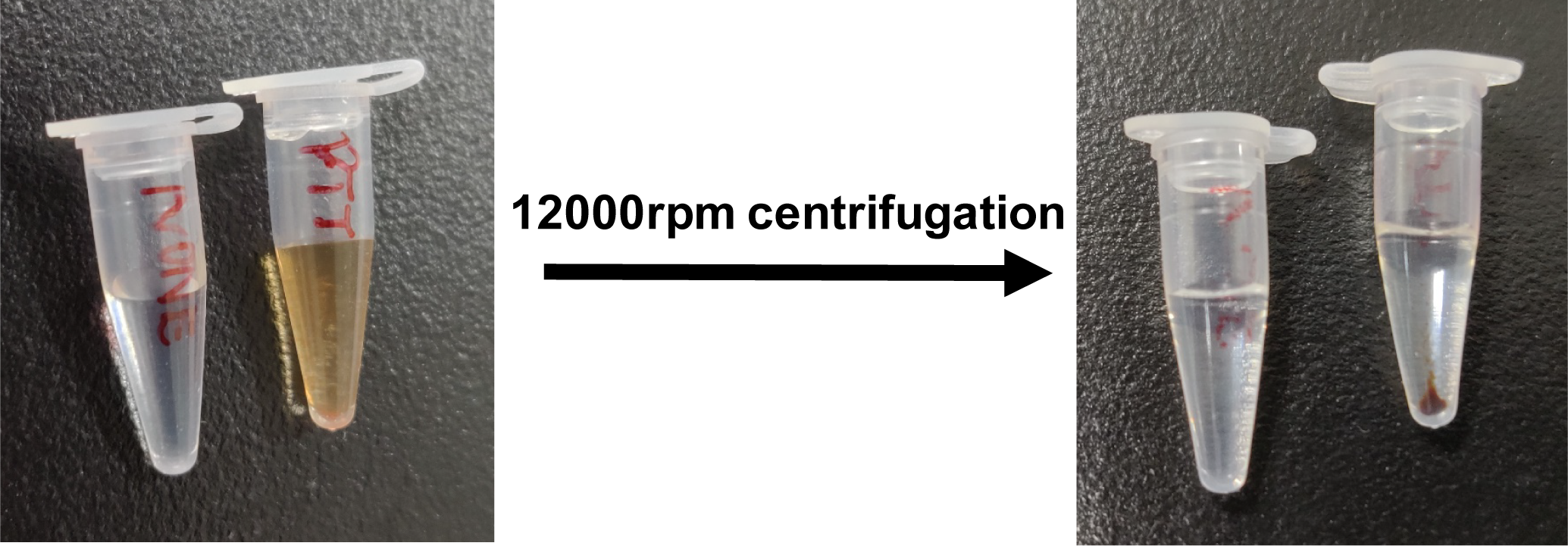

Supplement: Supplementary file 2 [file DataSheet4.ZIP › Supplement 4-1.tif]

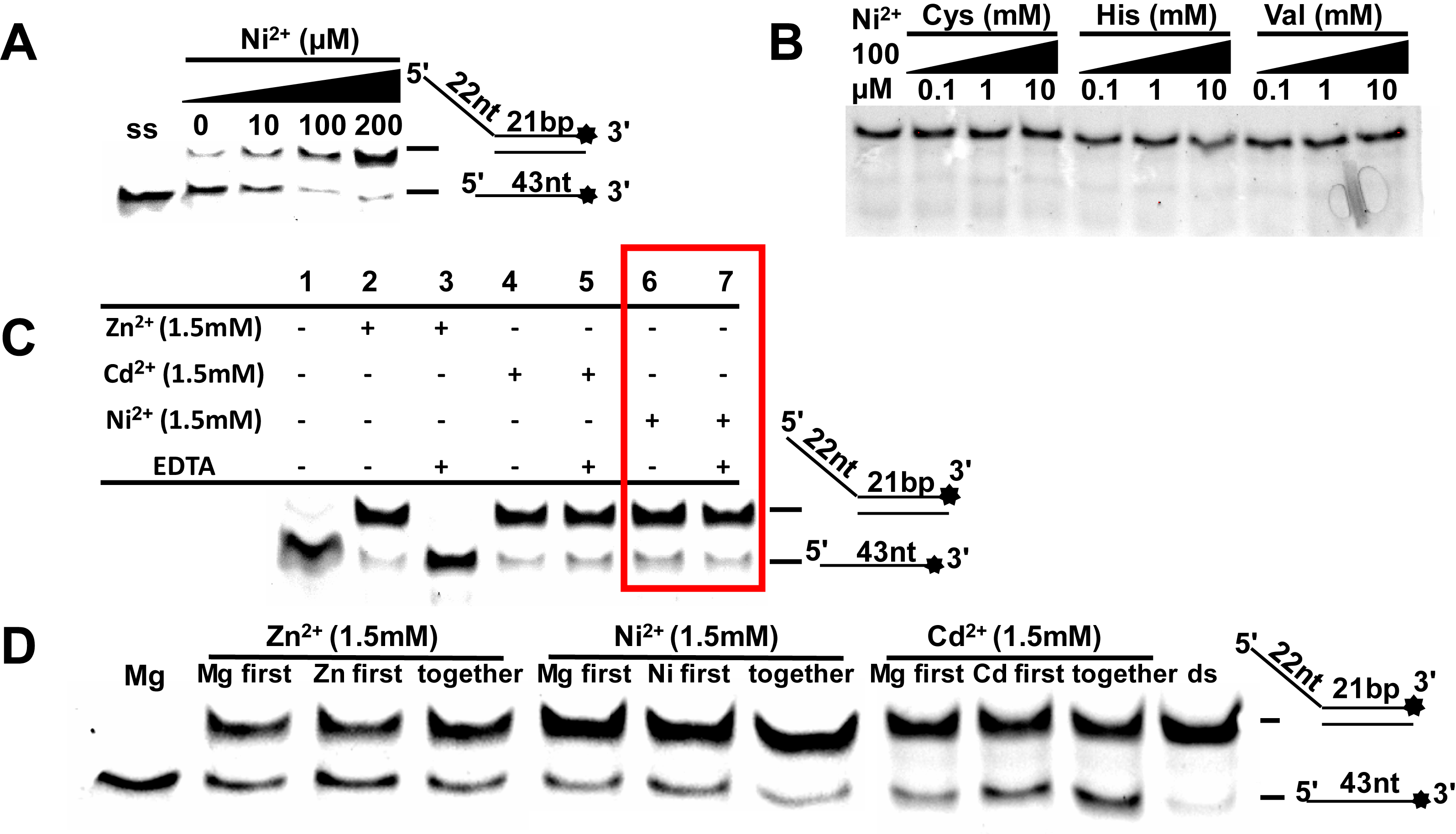

Supplement: Supplementary file 2 [file DataSheet4.ZIP › Supplement 4-2.tif]

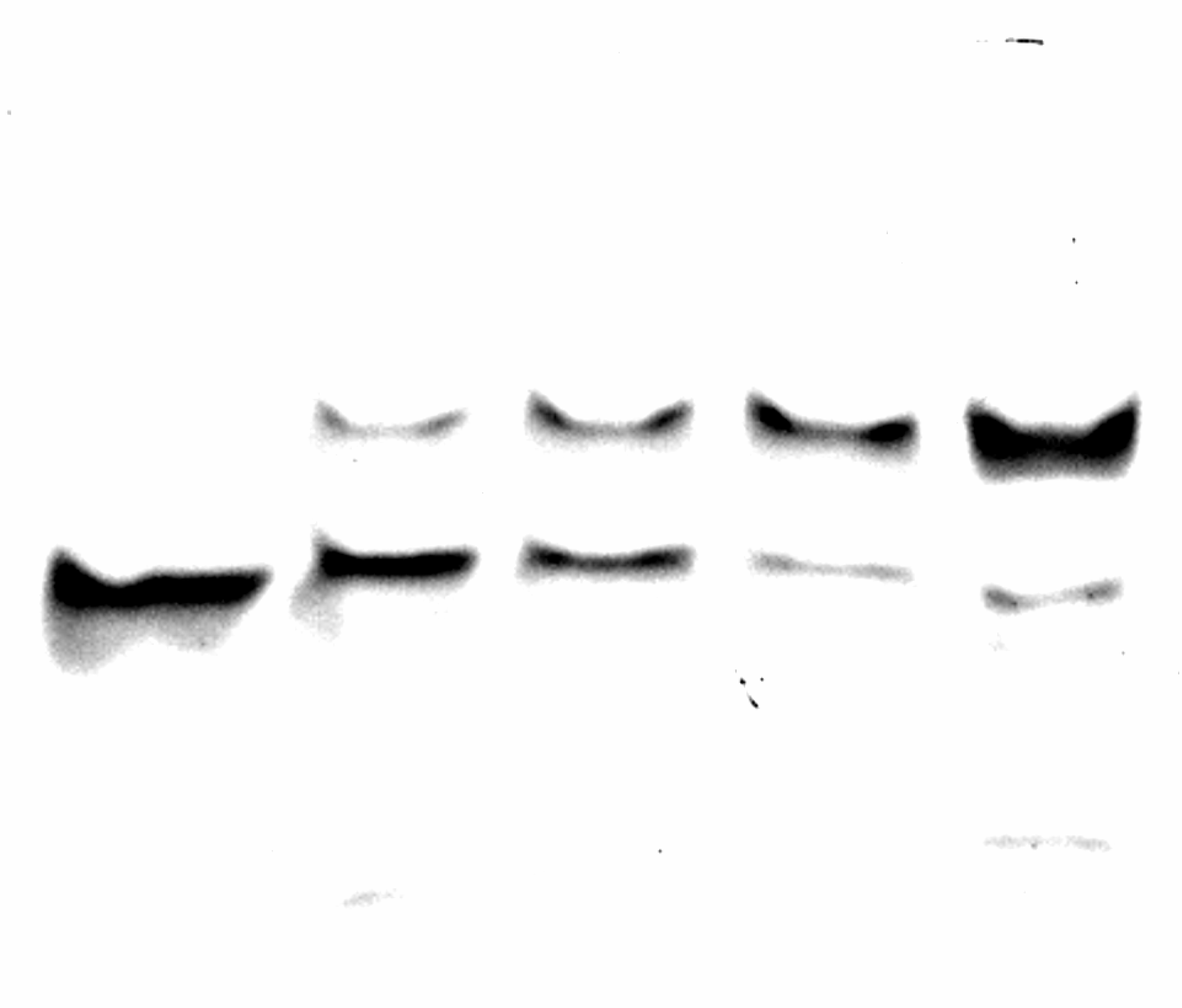

Supplement: Supplementary file 2 [file DataSheet4.ZIP › The original image/Supplement 4-2, A, the original image.tif]

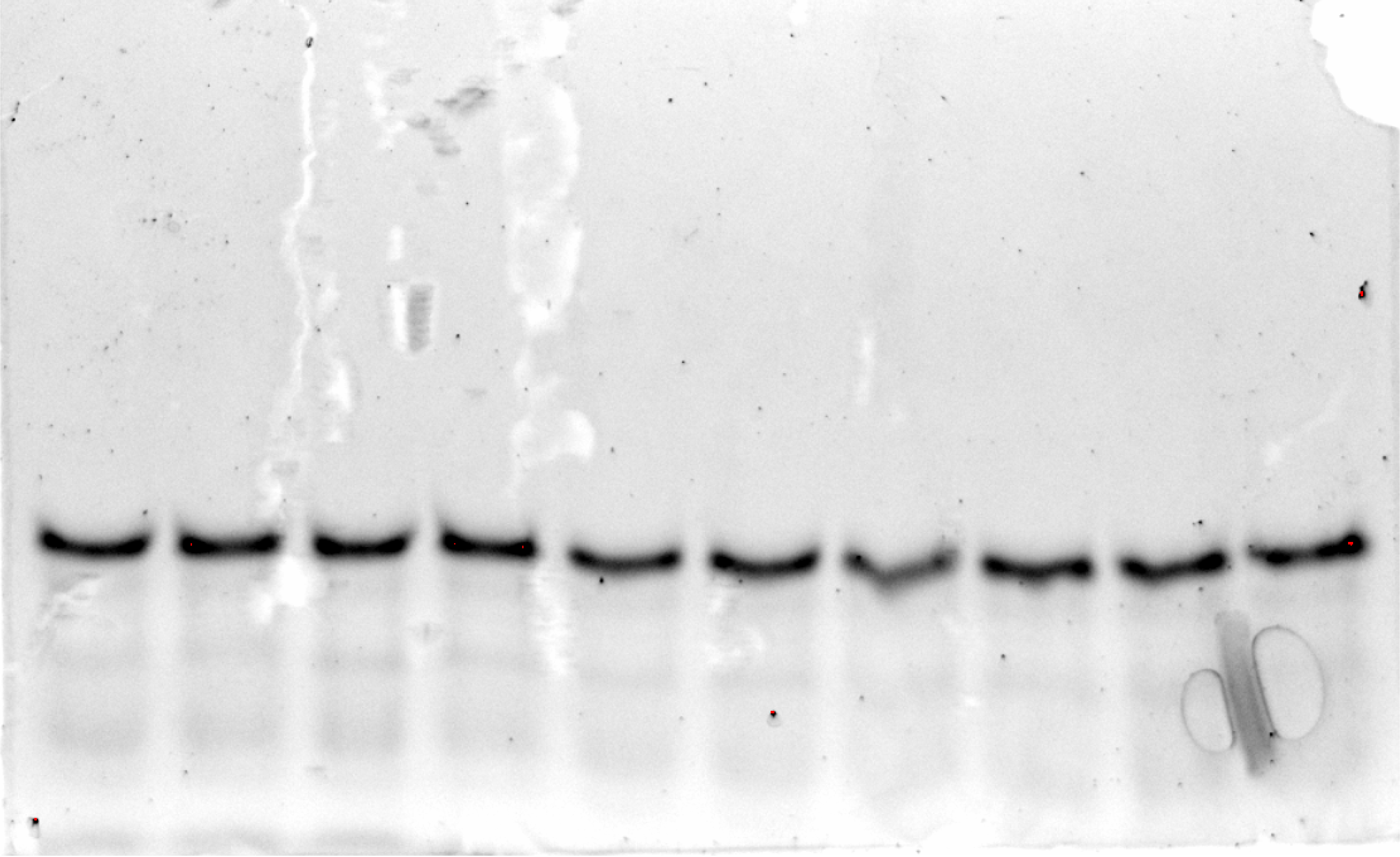

Supplement: Supplementary file 2 [file DataSheet4.ZIP › The original image/Supplement 4-2, B, the original image.tif]

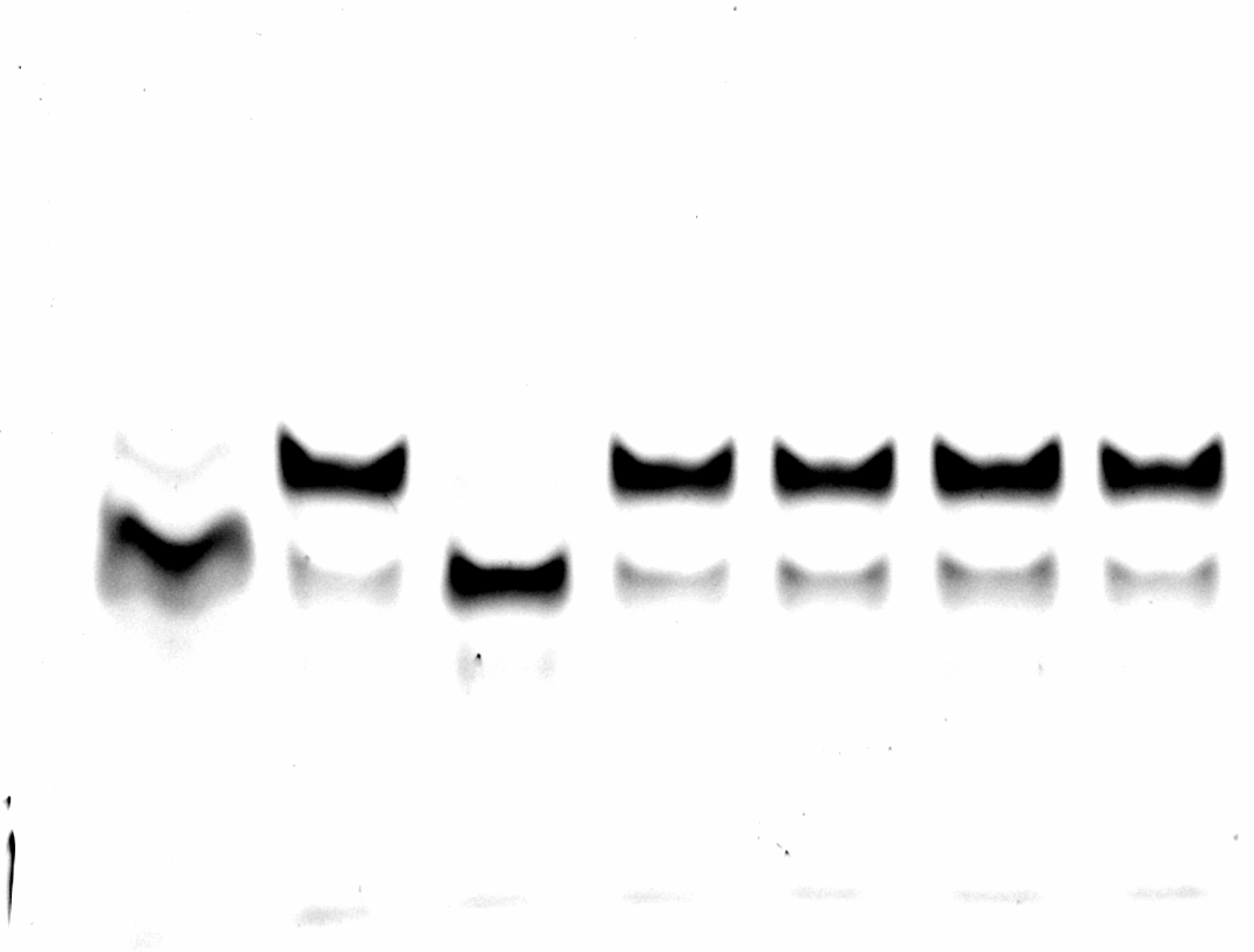

Supplement: Supplementary file 2 [file DataSheet4.ZIP › The original image/Supplement 4-2, C, the original image.tif]

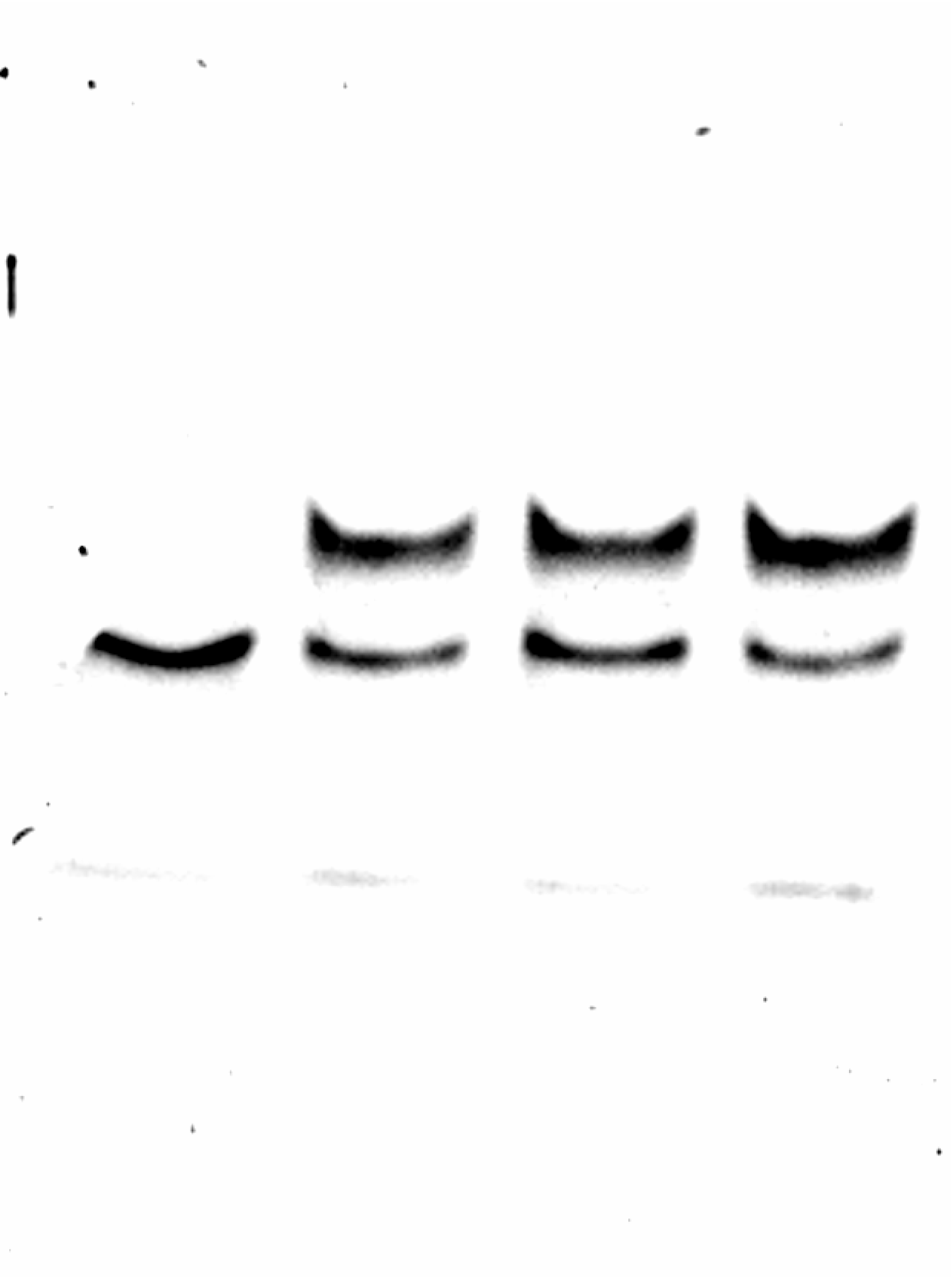

Supplement: Supplementary file 2 [file DataSheet4.ZIP › The original image/Supplement 4-2, D, lane 1-4,the original image.tif]

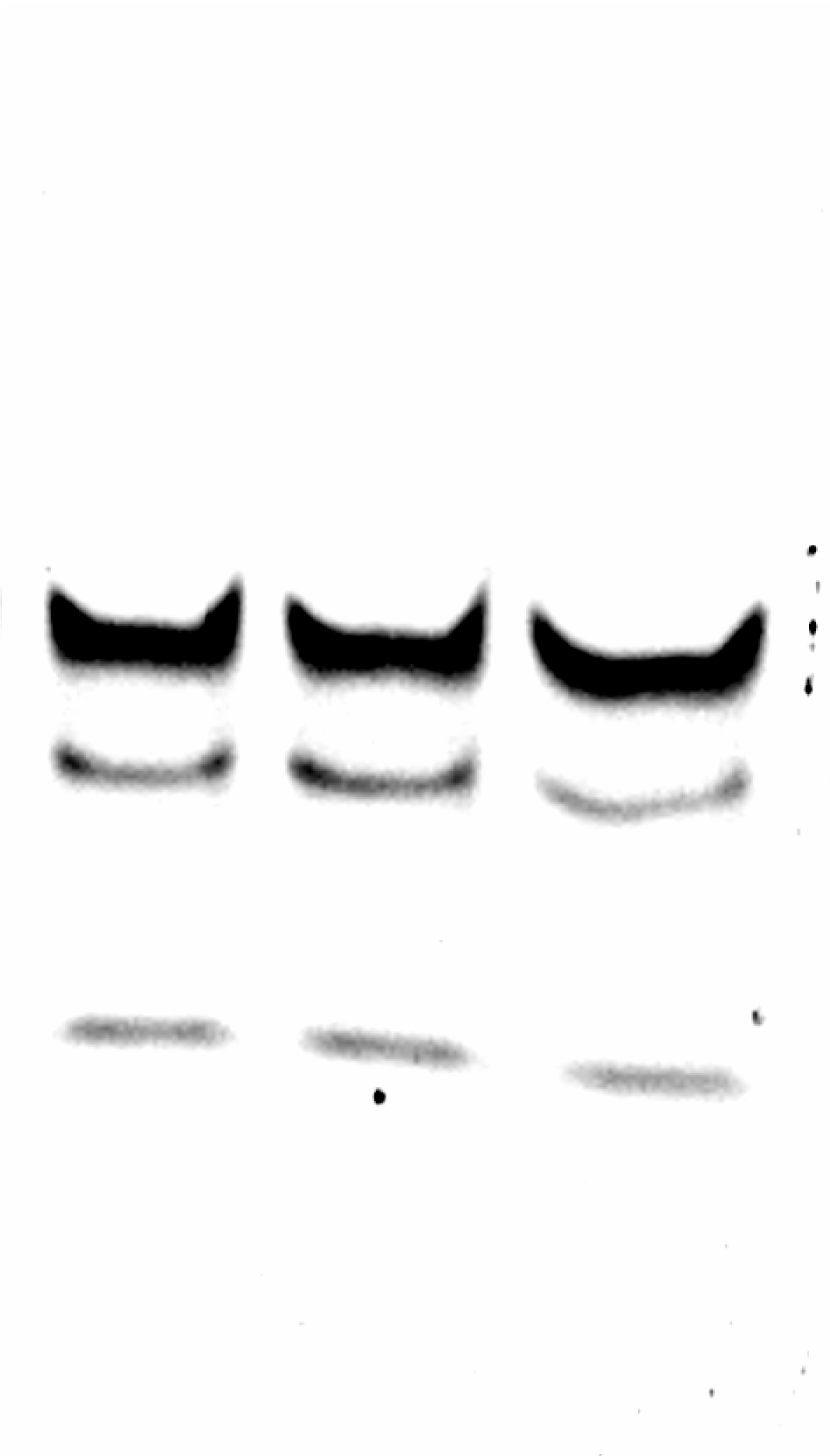

Supplement: Supplementary file 2 [file DataSheet4.ZIP › The original image/Supplement 4-2, D, lane 5-7,the original image.tif5.tif]

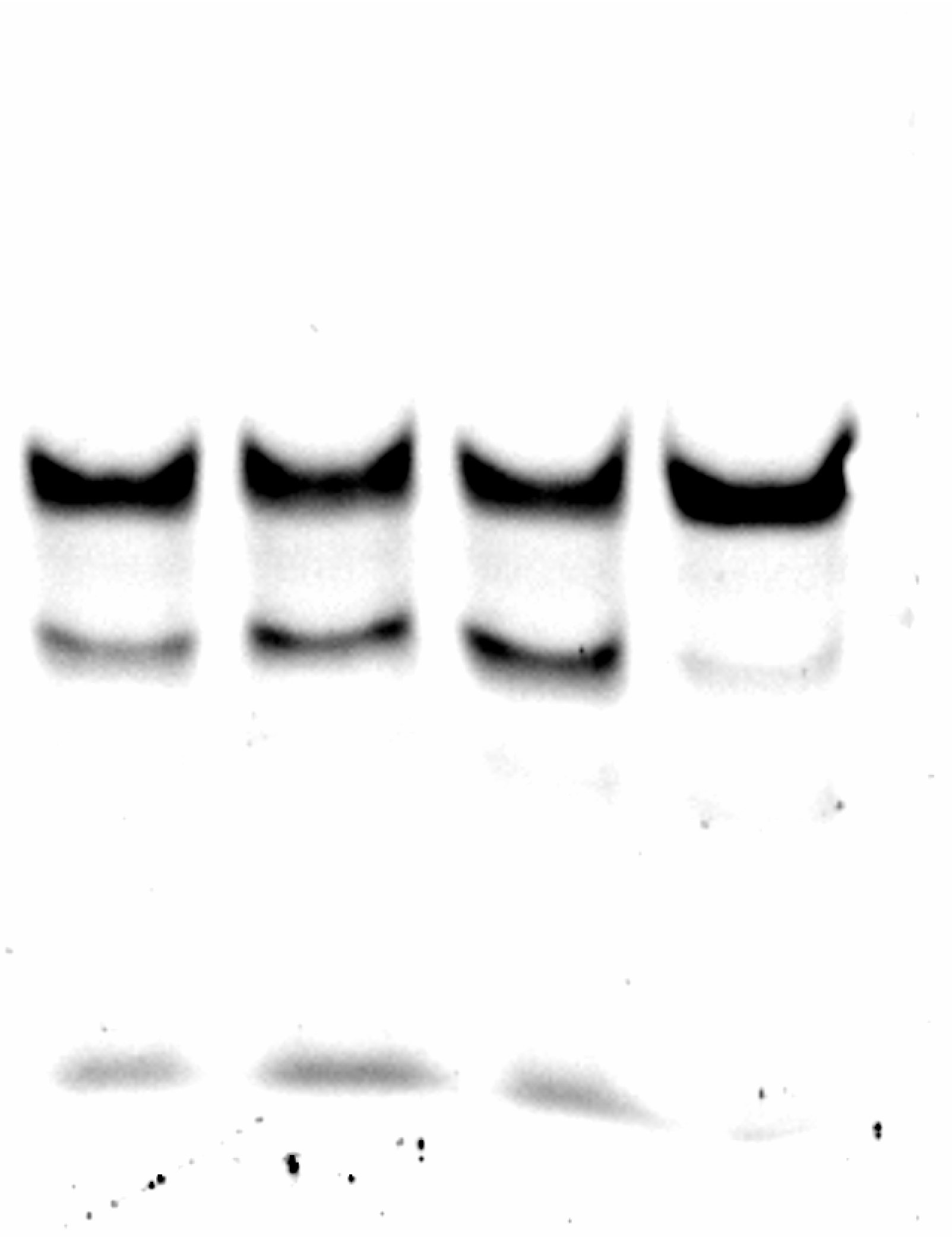

Supplement: Supplementary file 2 [file DataSheet4.ZIP › The original image/Supplement 4-2, D, lane 8-11,the original image.tif5.tif]

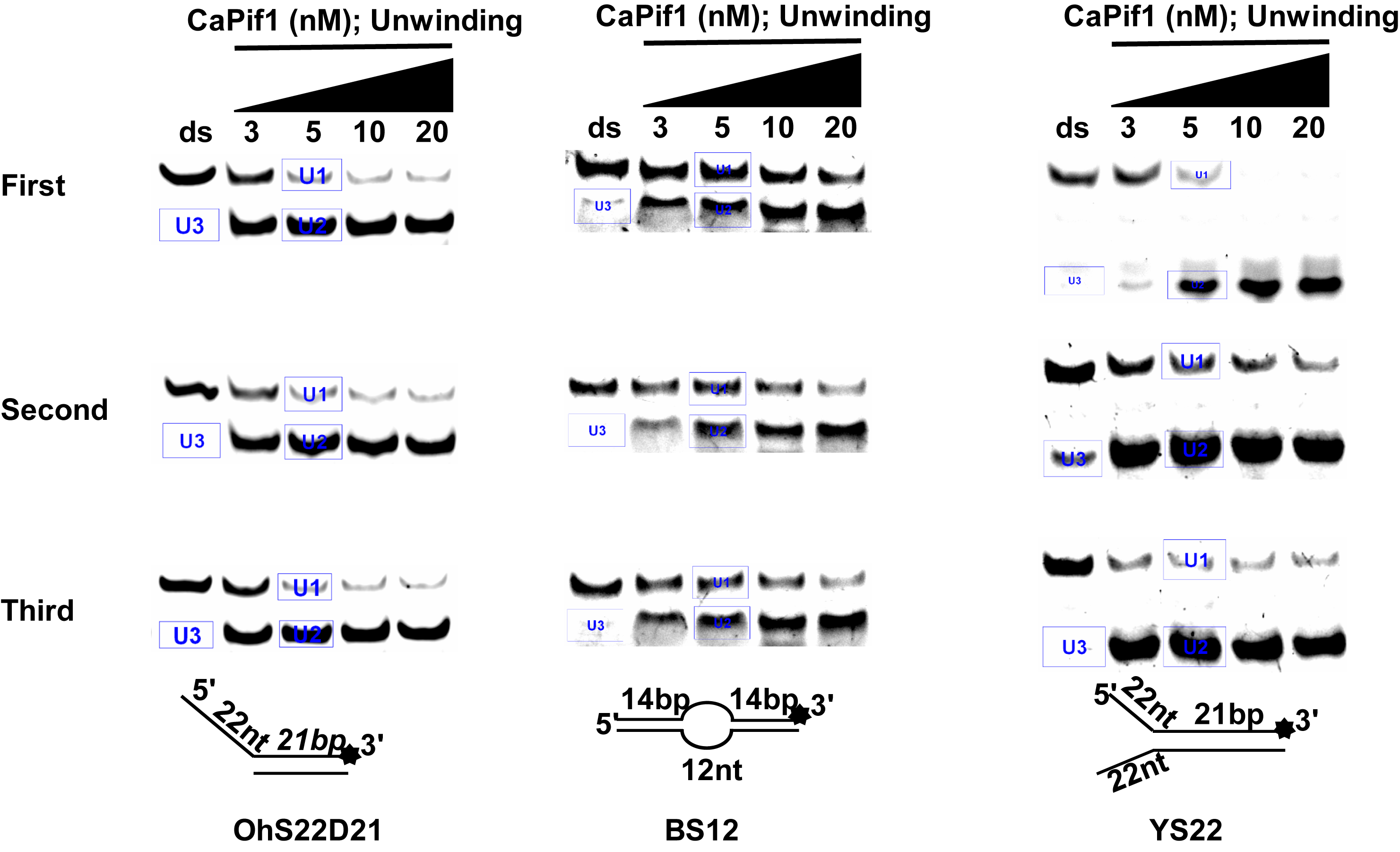

Supplement: Supplementary file 3 [file DataSheet1.ZIP › Supplement 1.tif]

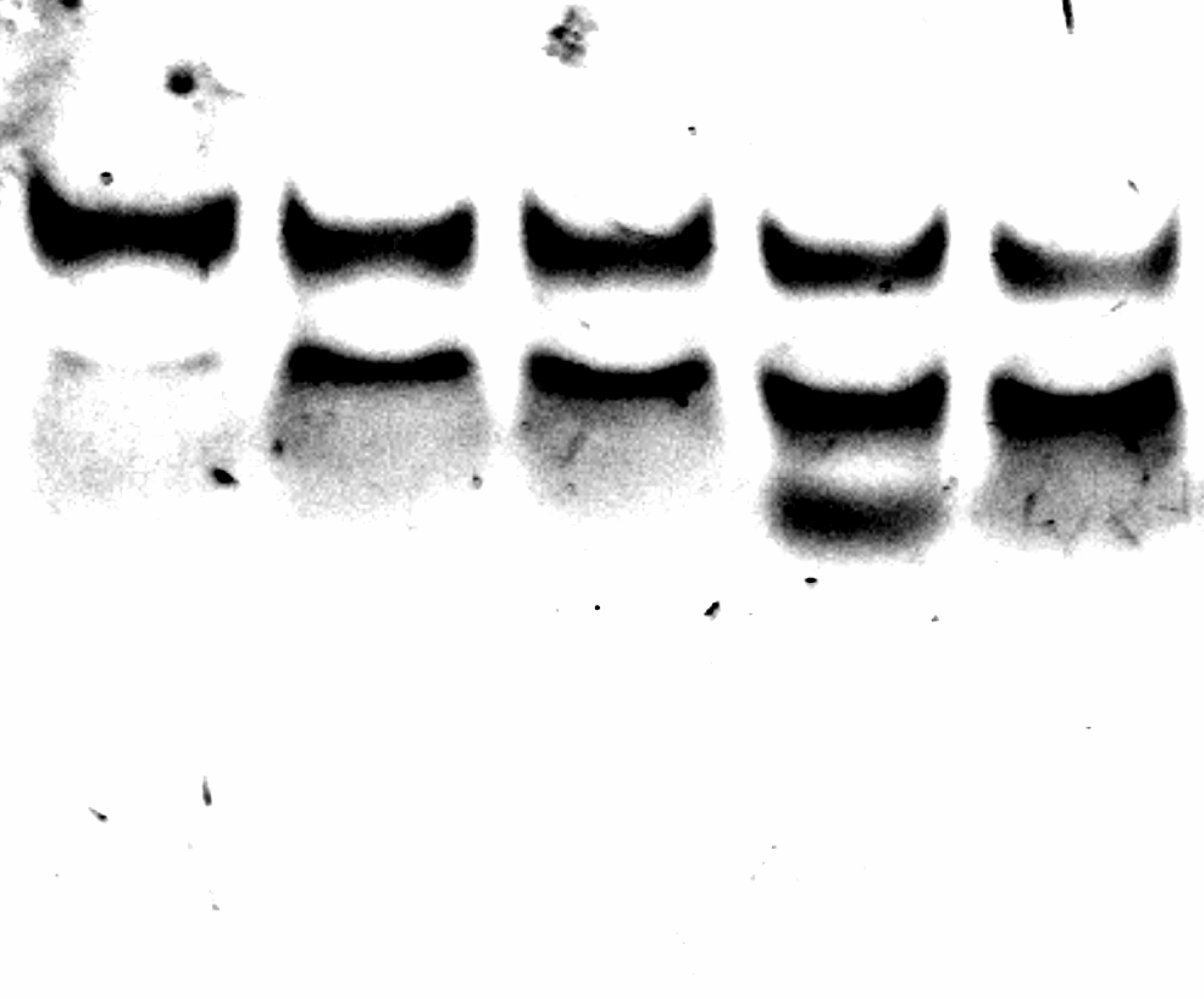

Supplement: Supplementary file 3 [file DataSheet1.ZIP › The original image/BS12-First, the original image.tif]

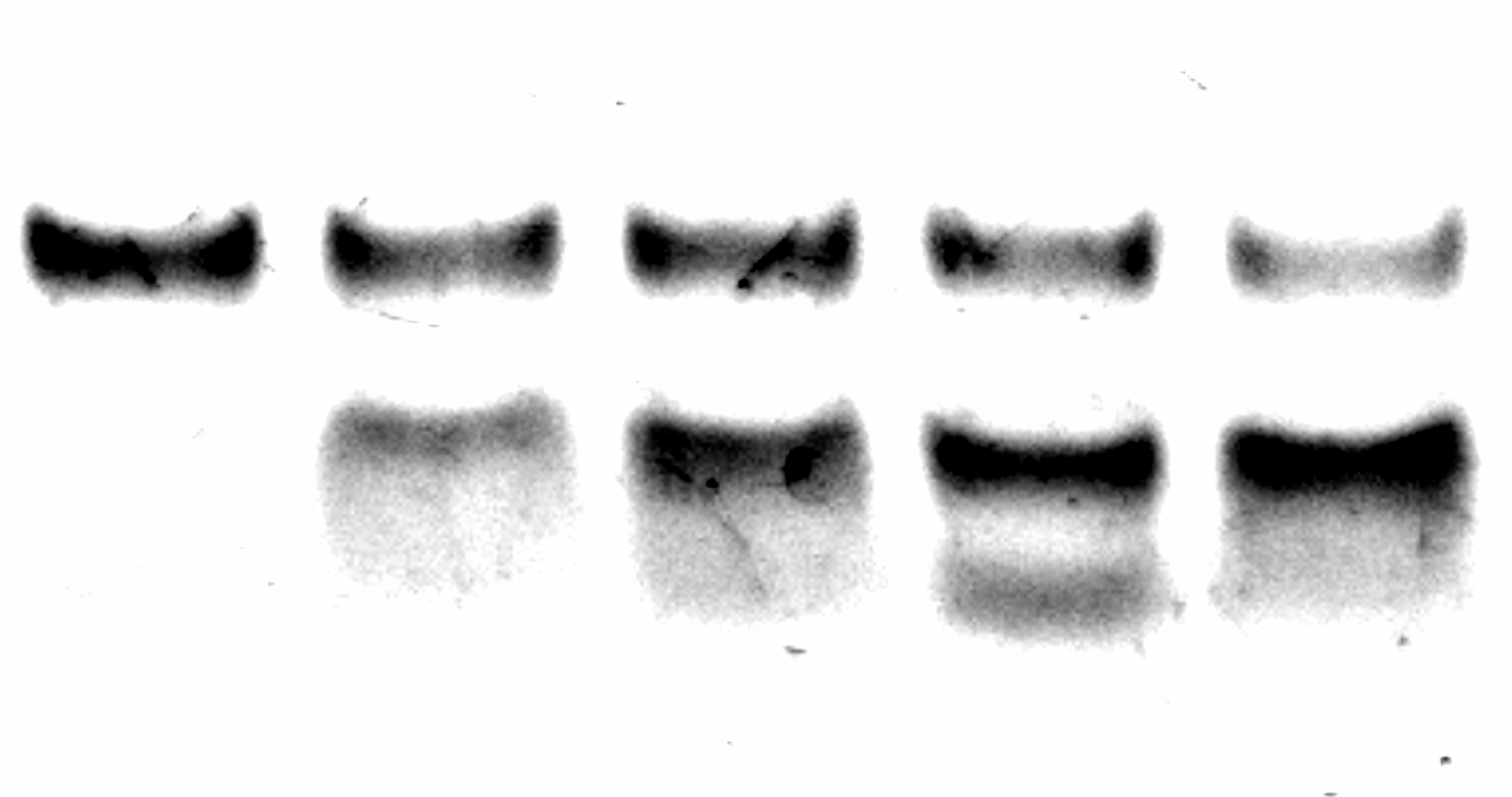

Supplement: Supplementary file 3 [file DataSheet1.ZIP › The original image/BS12-Second, the original image.tif]

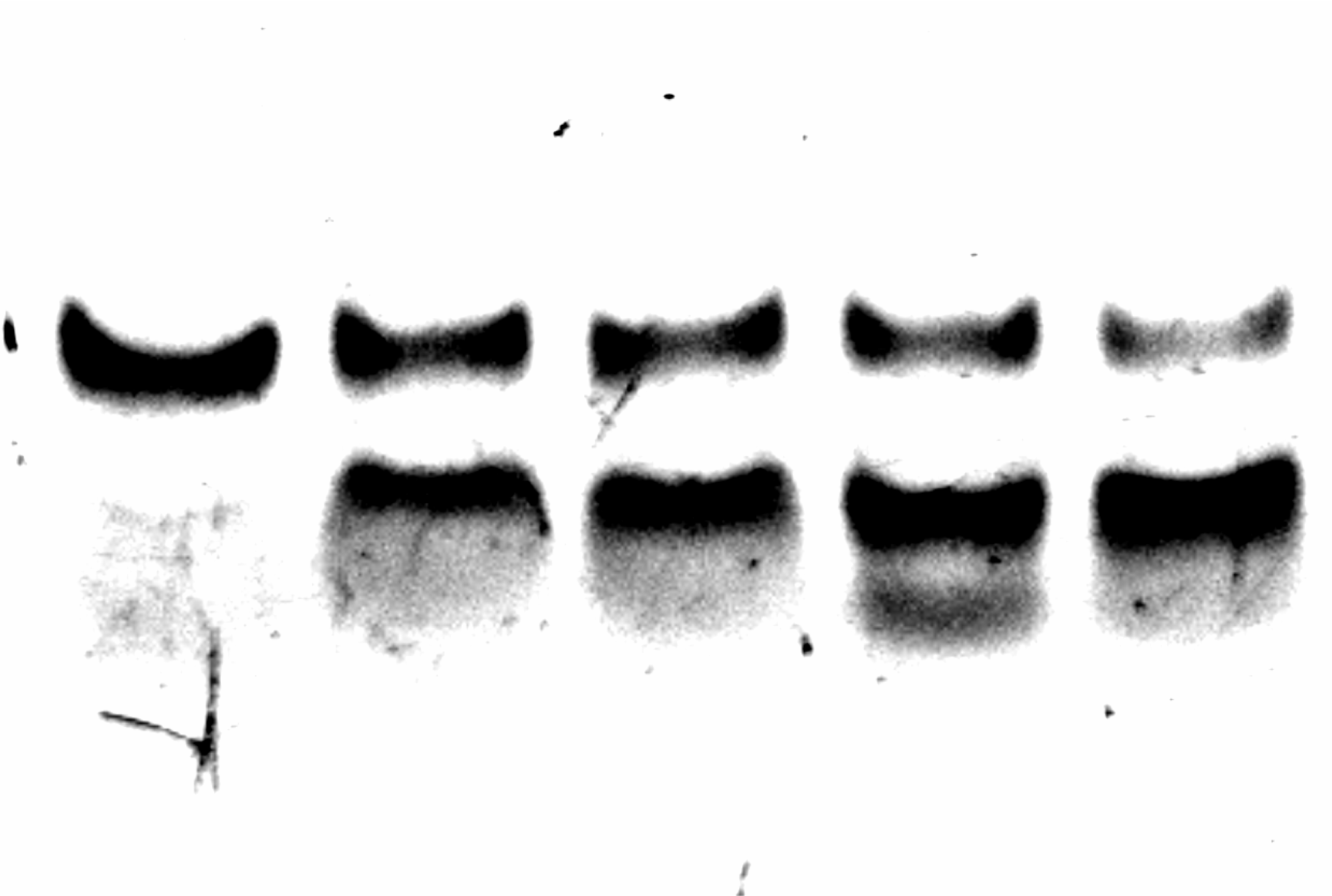

Supplement: Supplementary file 3 [file DataSheet1.ZIP › The original image/BS12-Third, the original image.tif]

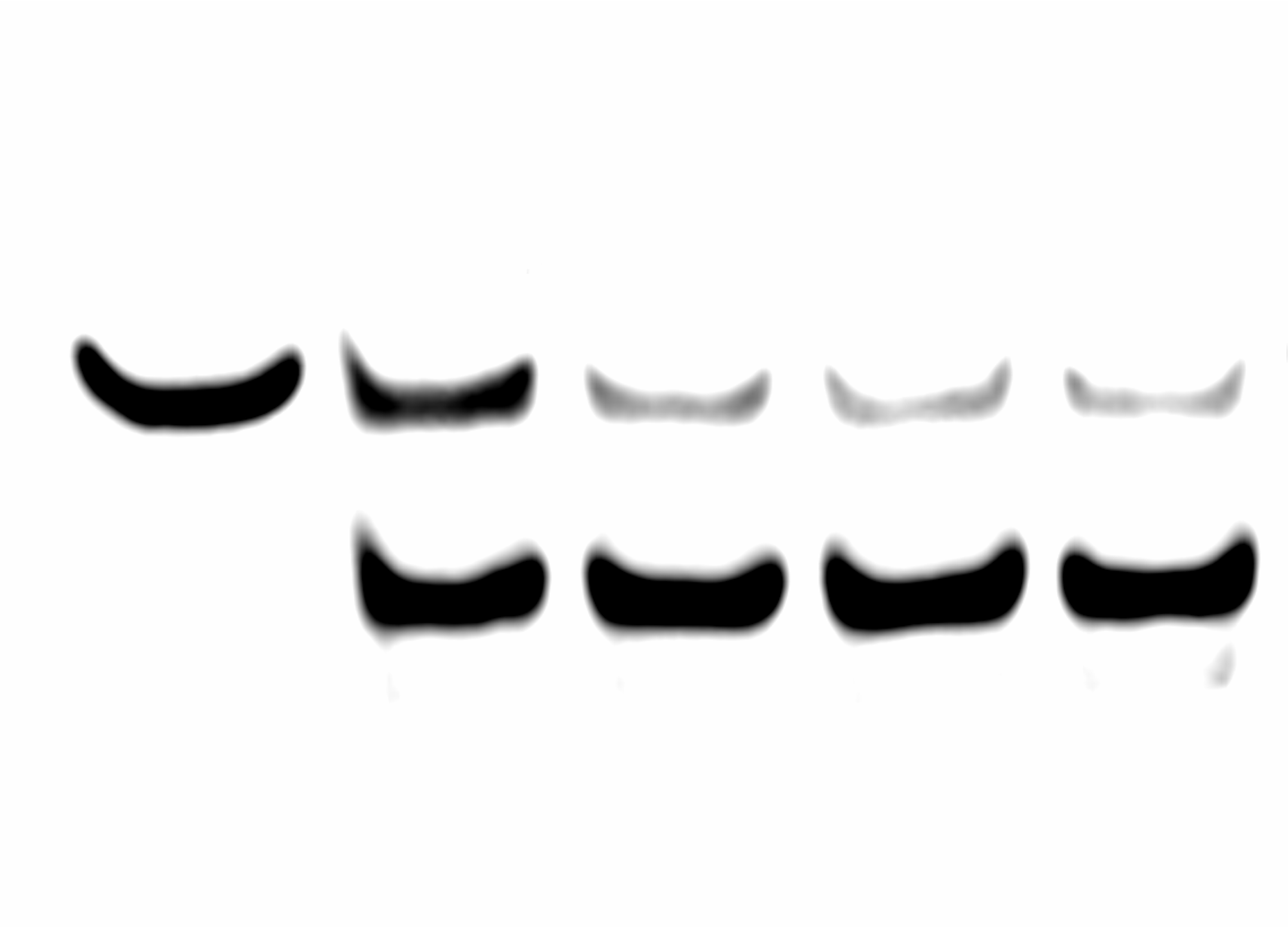

Supplement: Supplementary file 3 [file DataSheet1.ZIP › The original image/OhS22D21-First, the original image.tif]

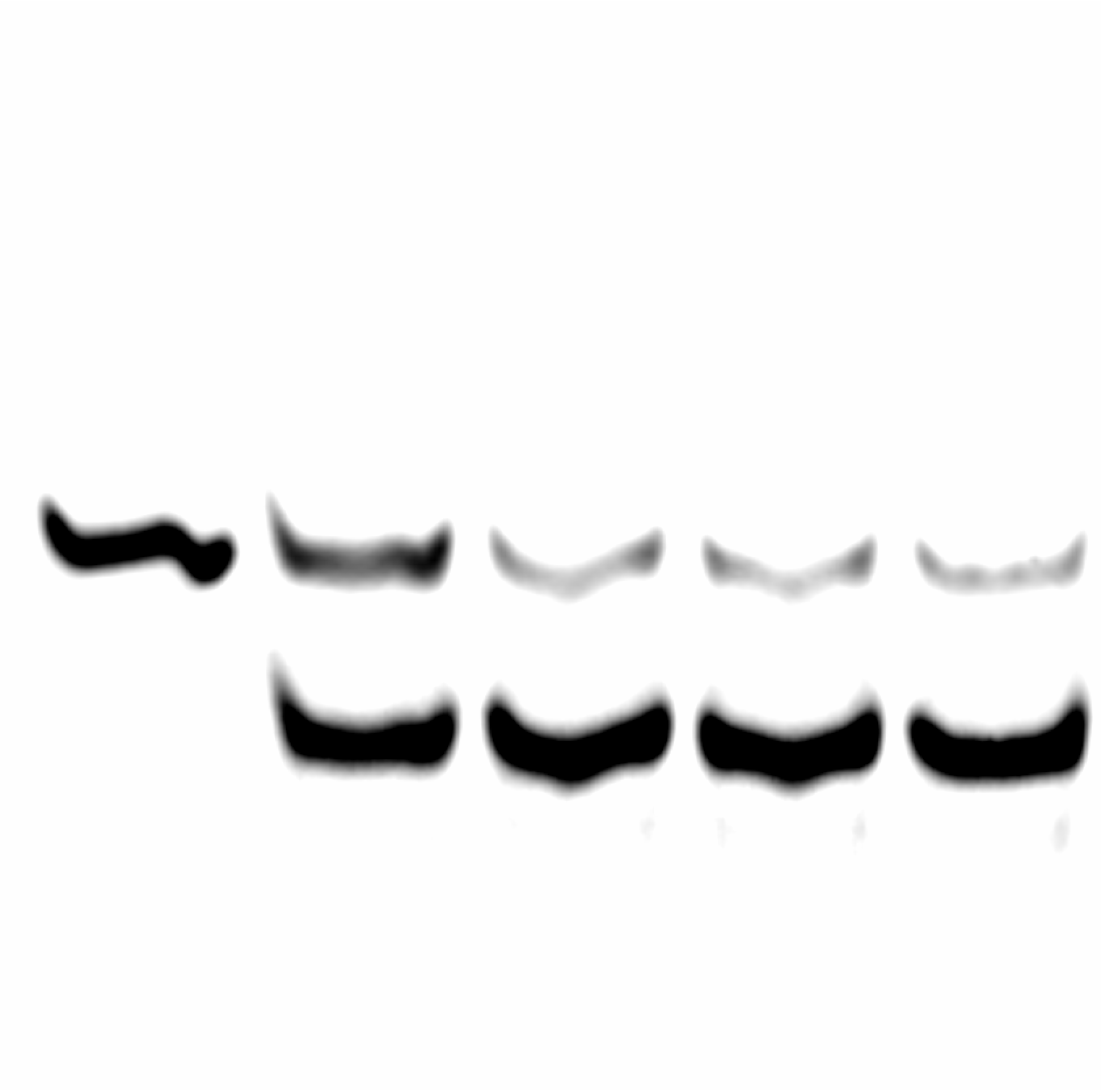

Supplement: Supplementary file 3 [file DataSheet1.ZIP › The original image/OhS22D21-Second, the original image.tif]

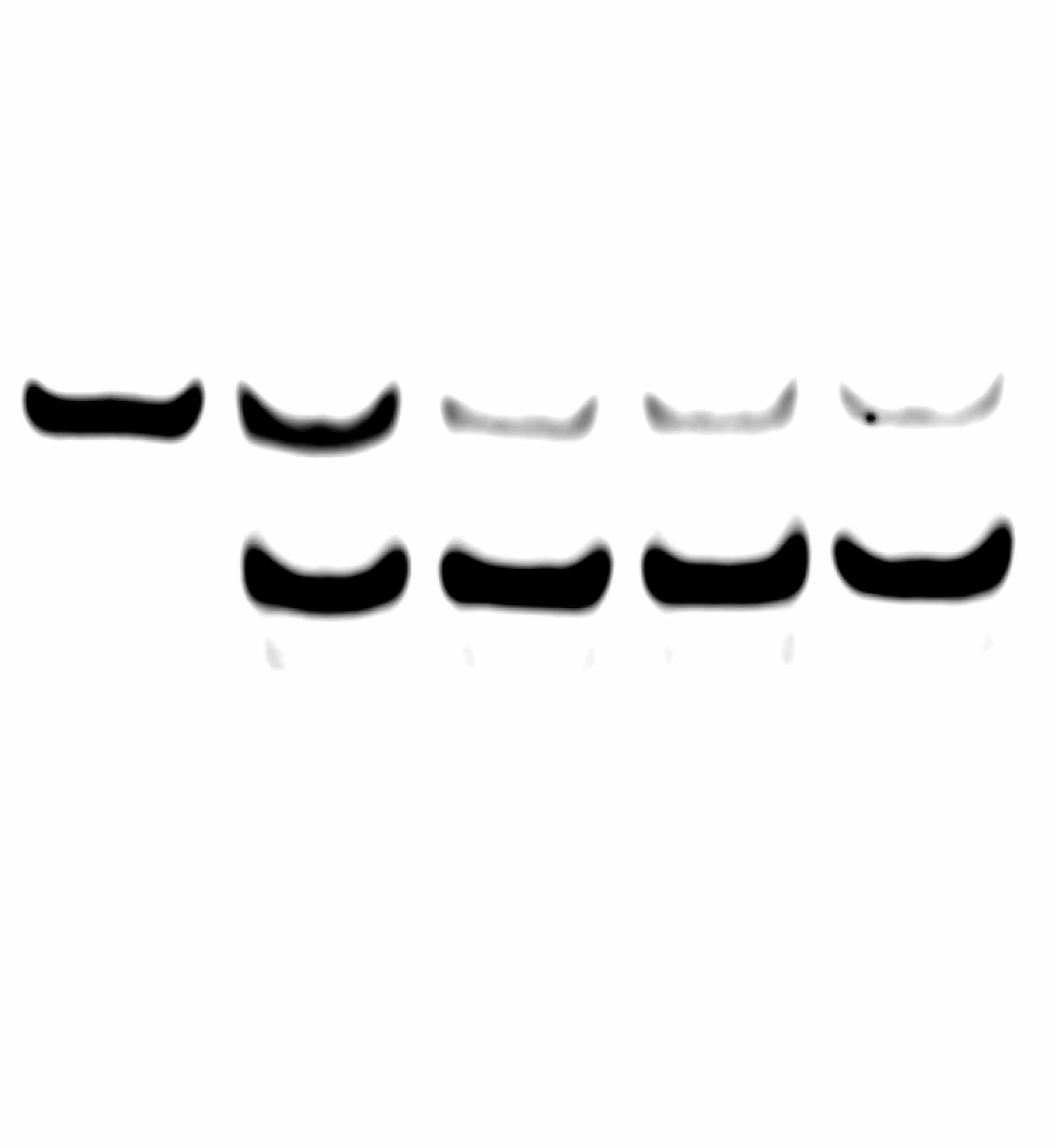

Supplement: Supplementary file 3 [file DataSheet1.ZIP › The original image/OhS22D21-Third, the original image.tif]

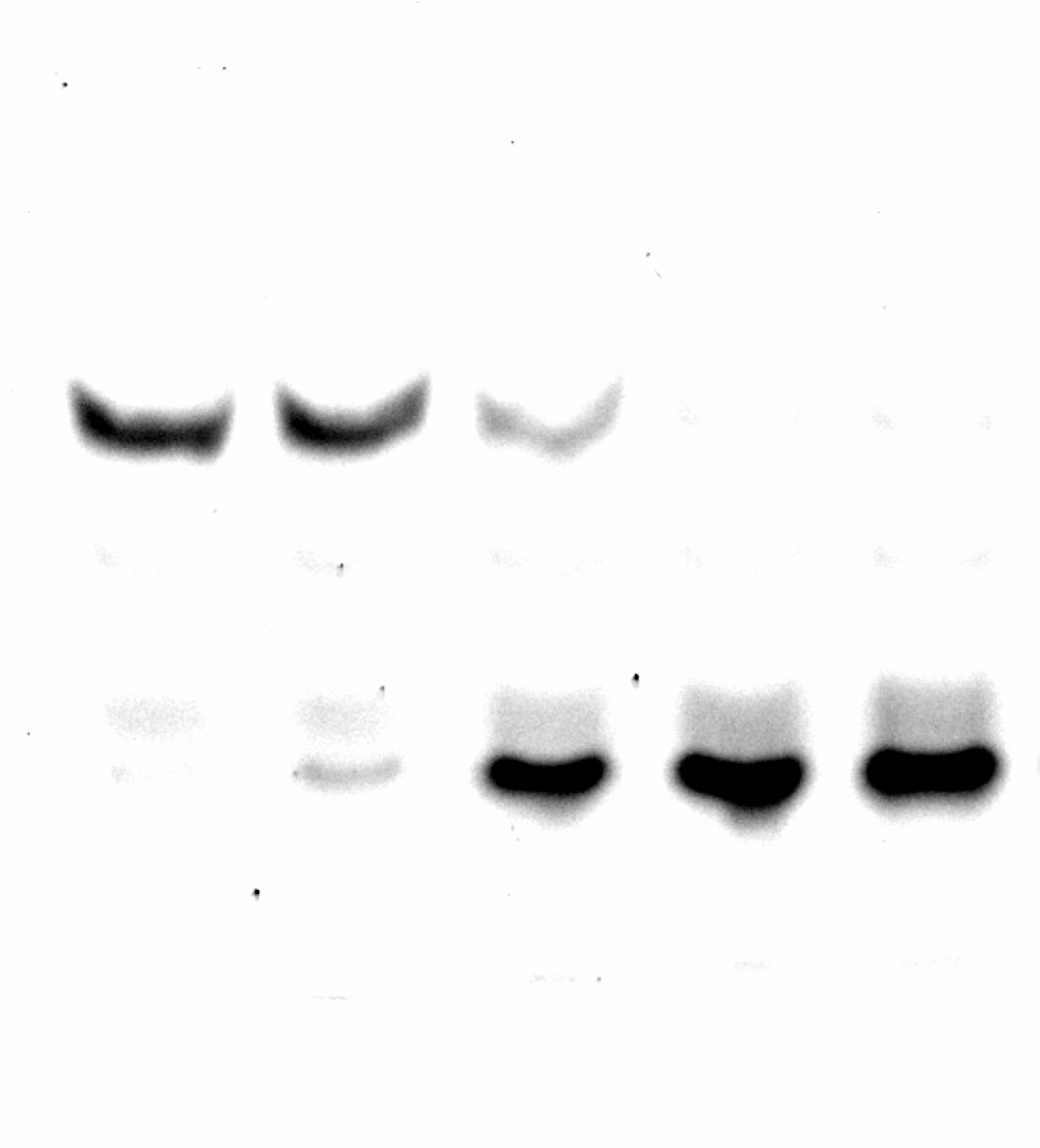

Supplement: Supplementary file 3 [file DataSheet1.ZIP › The original image/YS22-First, the original image.tif]

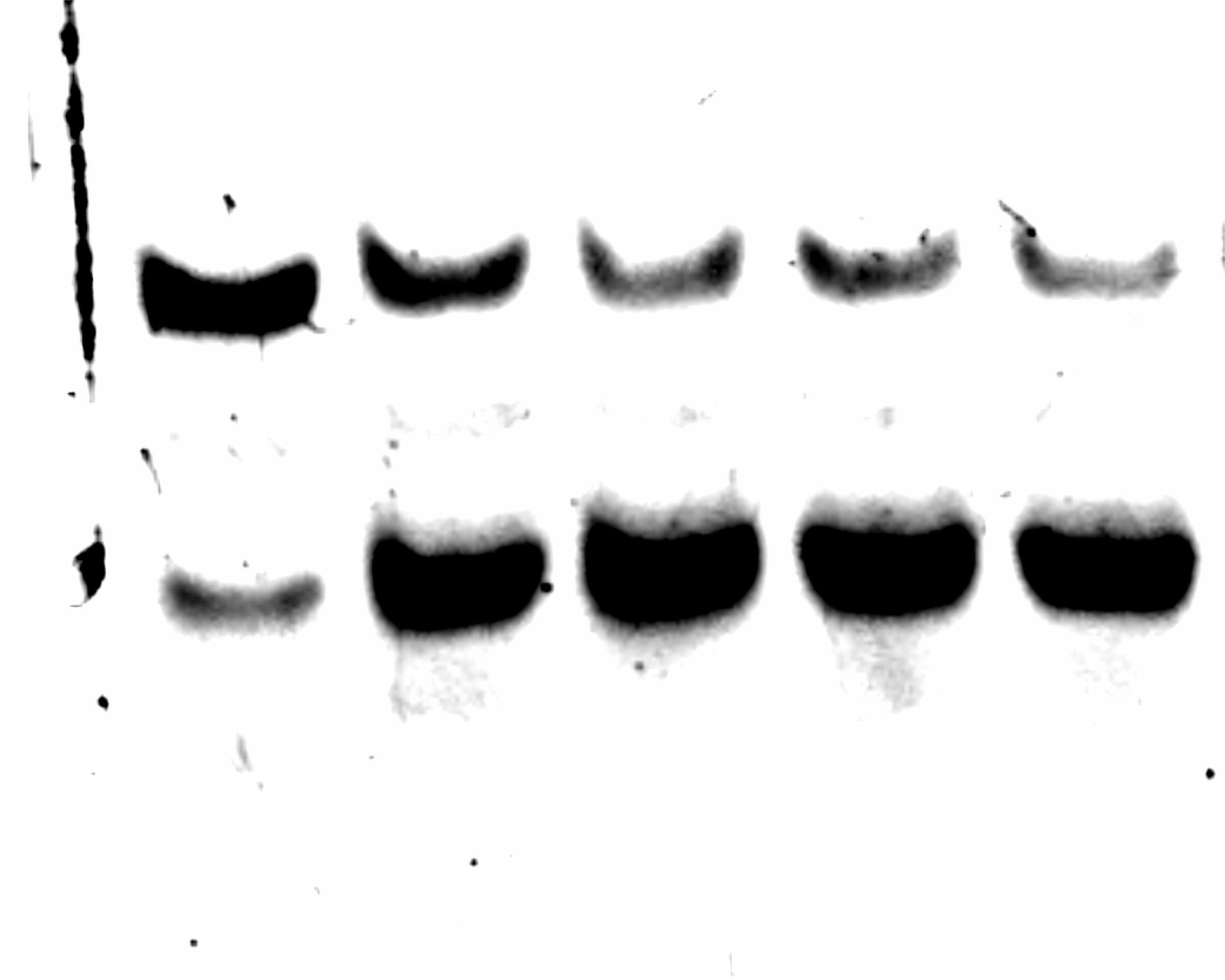

Supplement: Supplementary file 3 [file DataSheet1.ZIP › The original image/YS22-Second, the original image.tif]

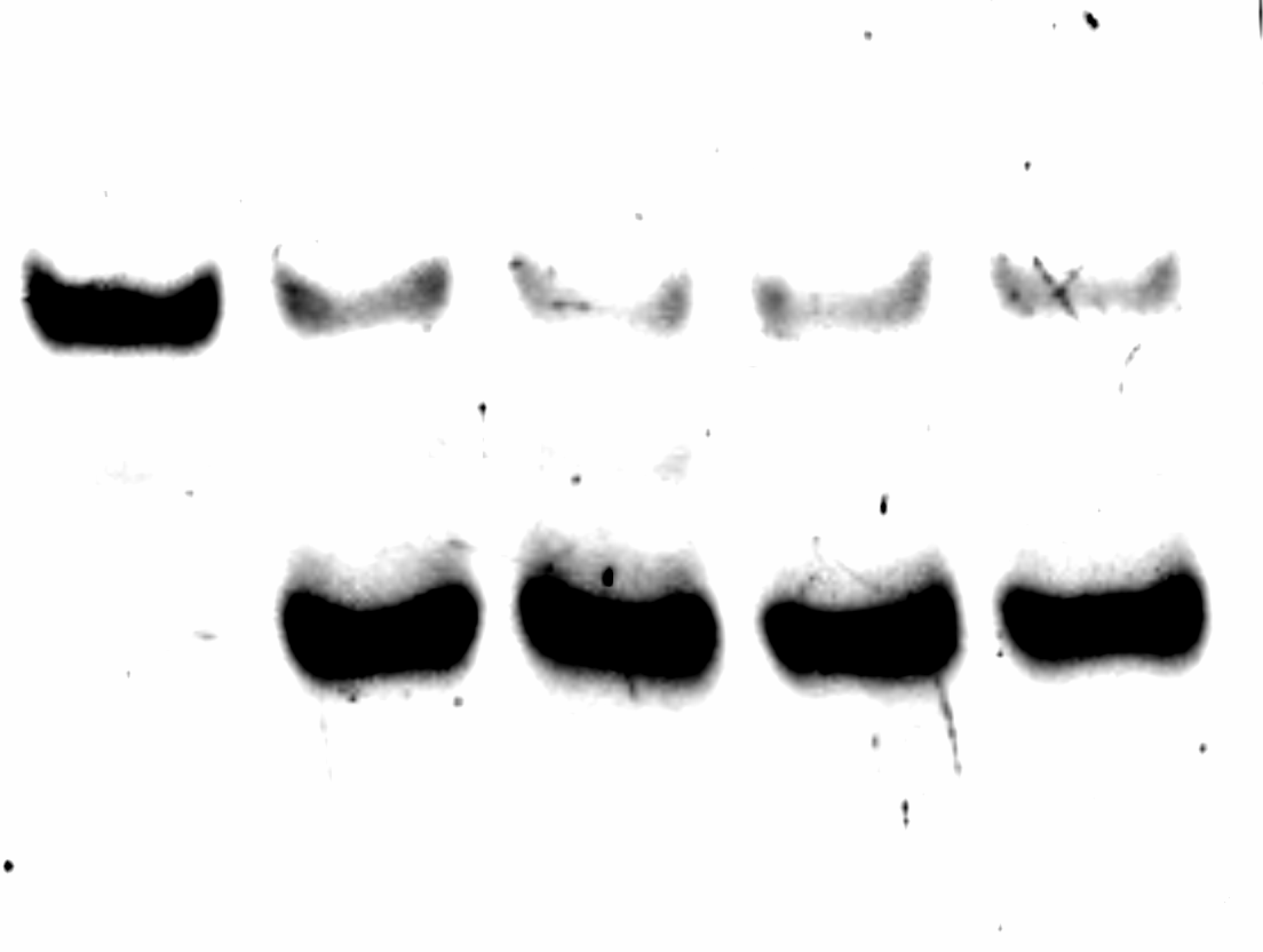

Supplement: Supplementary file 3 [file DataSheet1.ZIP › The original image/YS22-Third, the original image.tif]

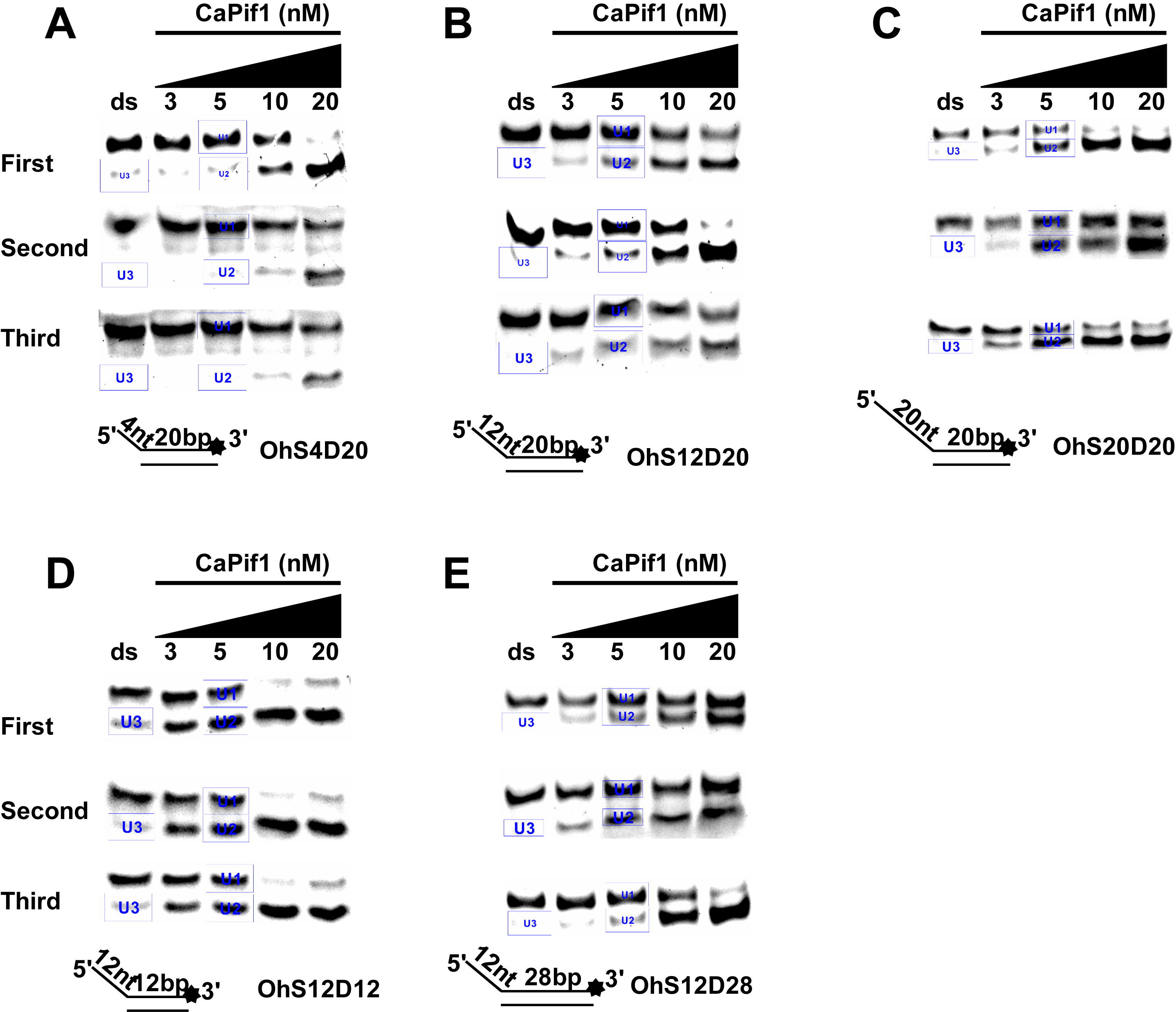

Supplement: Supplementary file 4 [file DataSheet2.ZIP › Supplement 2.tif]

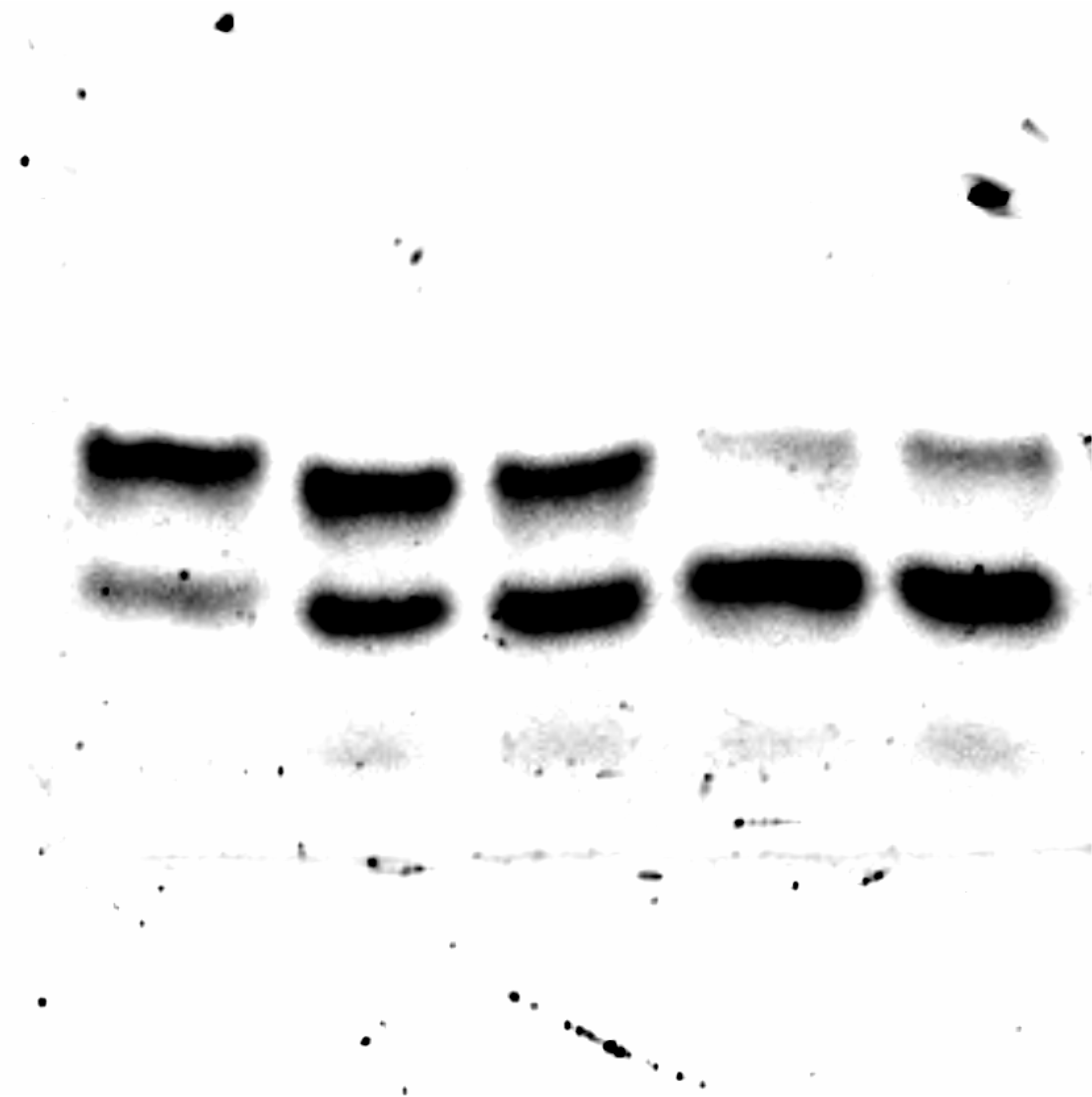

Supplement: Supplementary file 4 [file DataSheet2.ZIP › The original image/OhS12D12,first,The original image.tif]

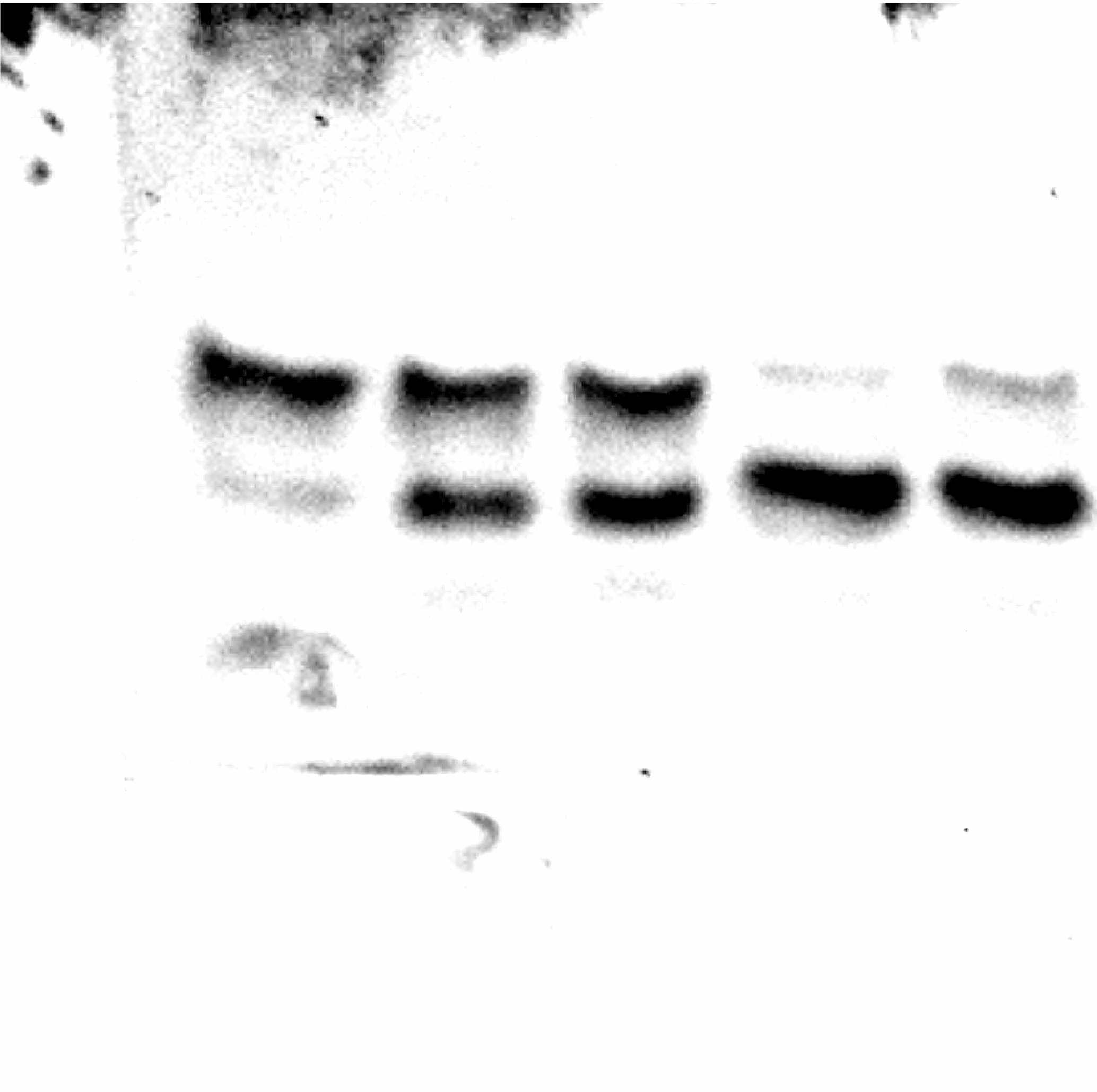

Supplement: Supplementary file 4 [file DataSheet2.ZIP › The original image/OhS12D12,second,The original image.tif]

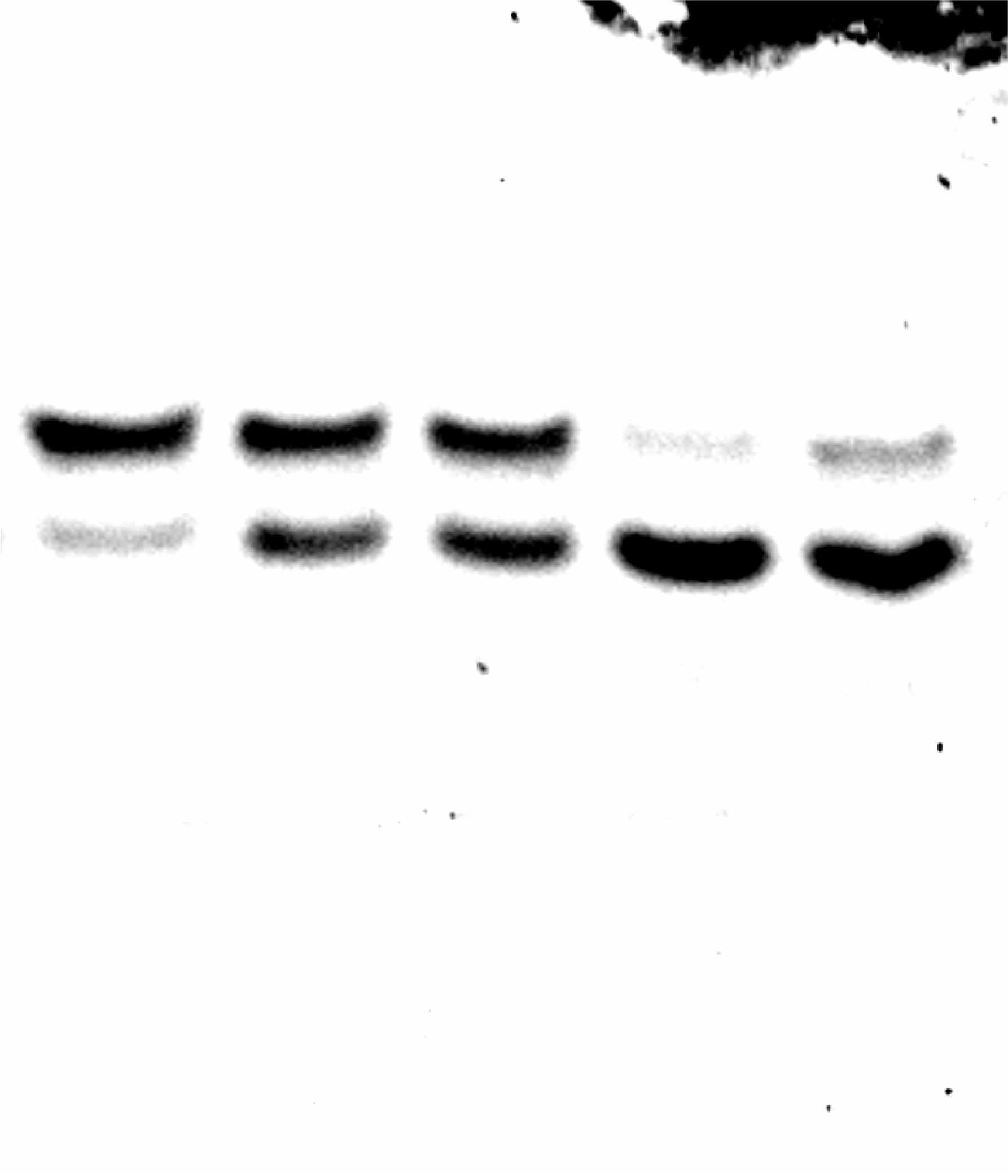

Supplement: Supplementary file 4 [file DataSheet2.ZIP › The original image/OhS12D12,third,The original image.tif]

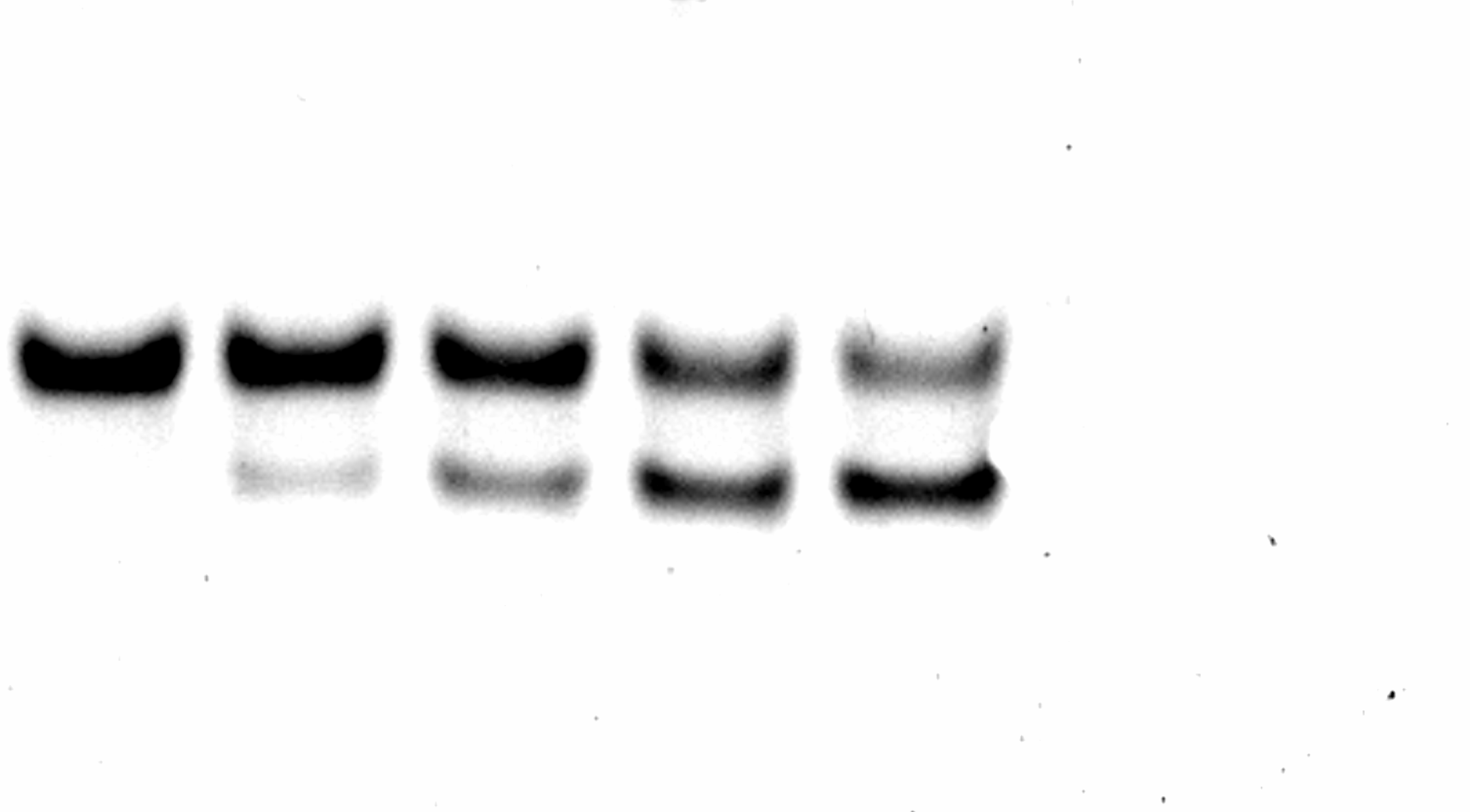

Supplement: Supplementary file 4 [file DataSheet2.ZIP › The original image/OhS12D20,first,The original image.tif]

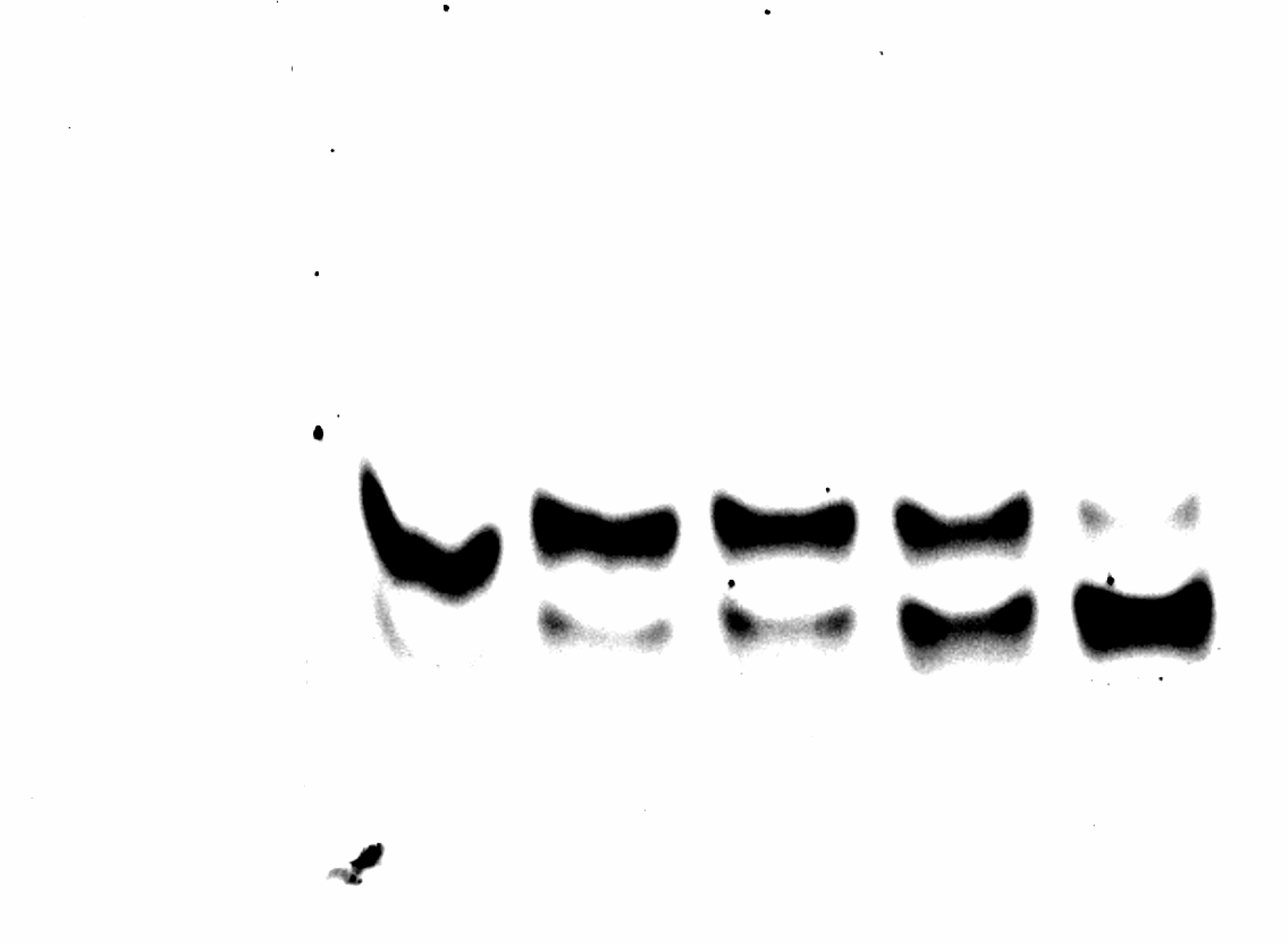

Supplement: Supplementary file 4 [file DataSheet2.ZIP › The original image/OhS12D20,second,The original image.tif]

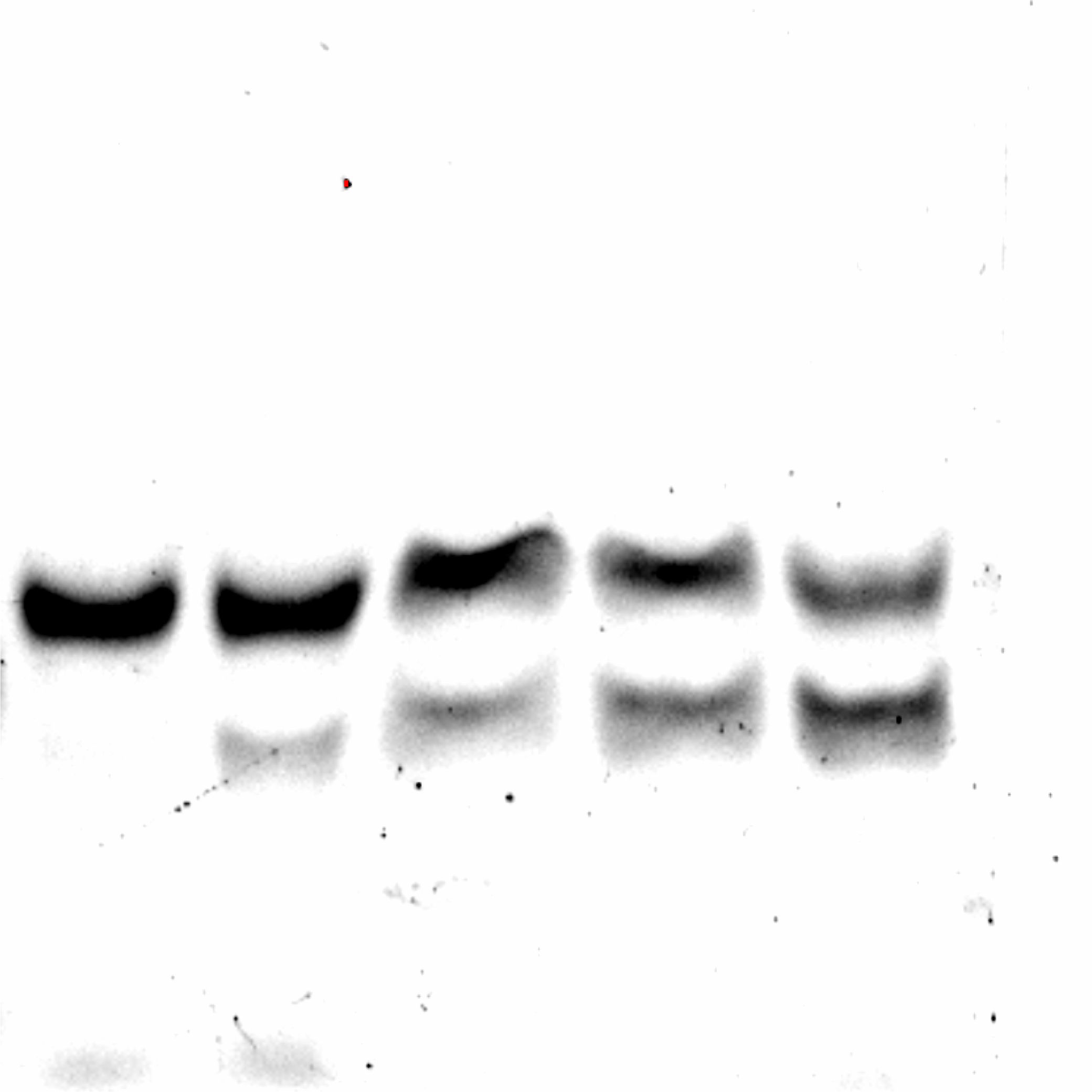

Supplement: Supplementary file 4 [file DataSheet2.ZIP › The original image/OhS12D20,third,The original image.tif]

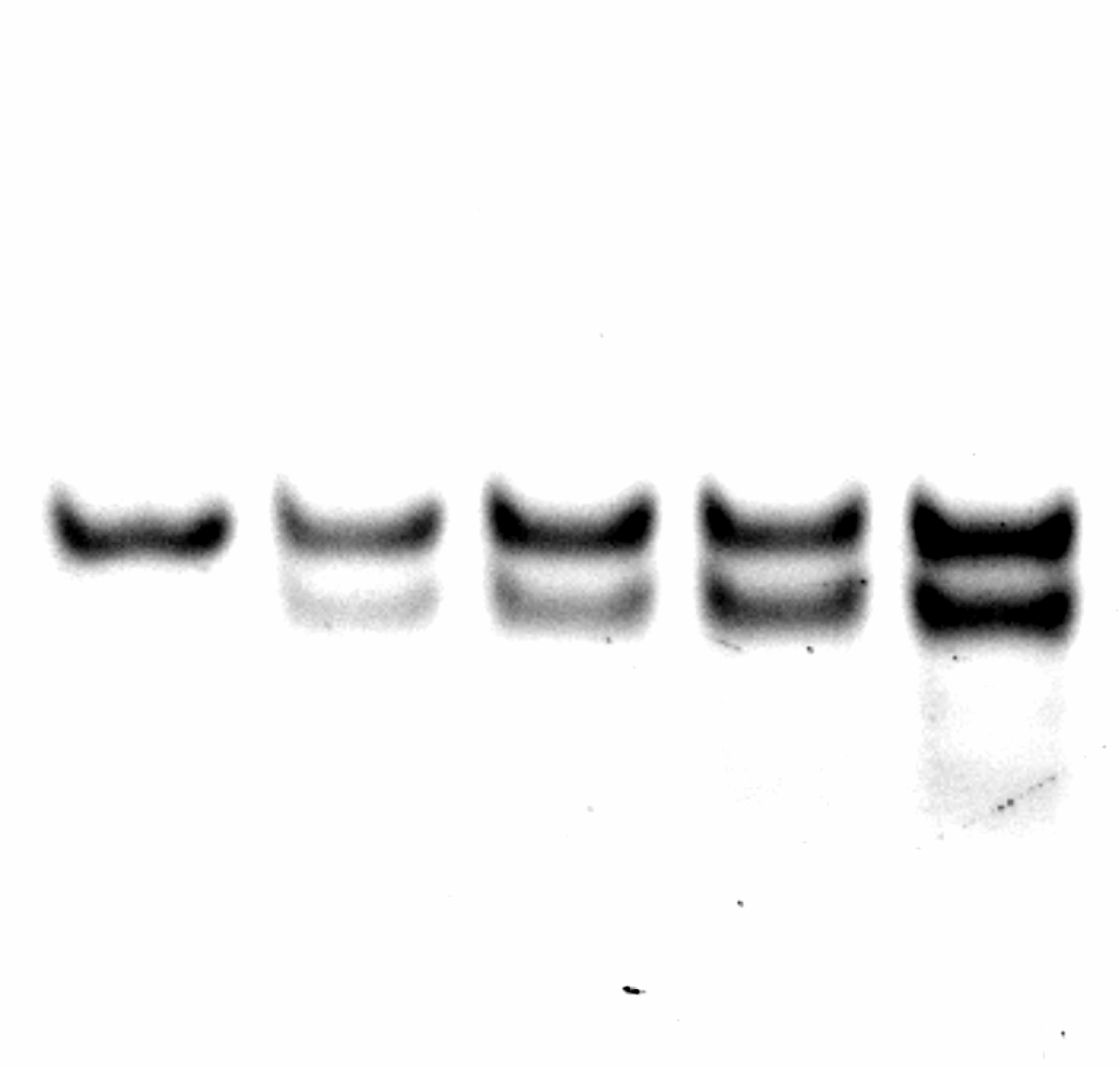

Supplement: Supplementary file 4 [file DataSheet2.ZIP › The original image/OhS12D28,first,The original image.tif]

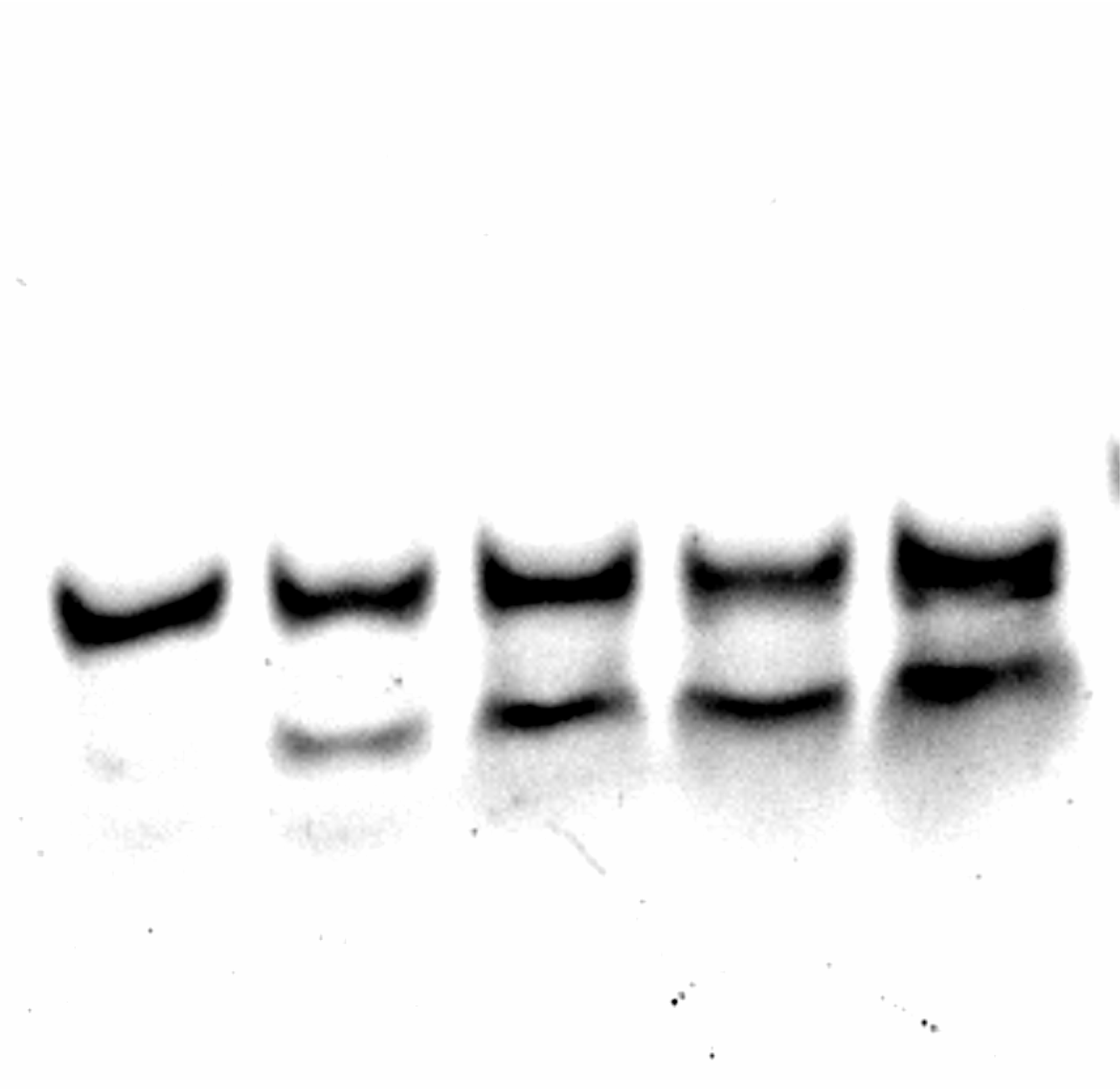

Supplement: Supplementary file 4 [file DataSheet2.ZIP › The original image/OhS12D28,second,The original image.tif]

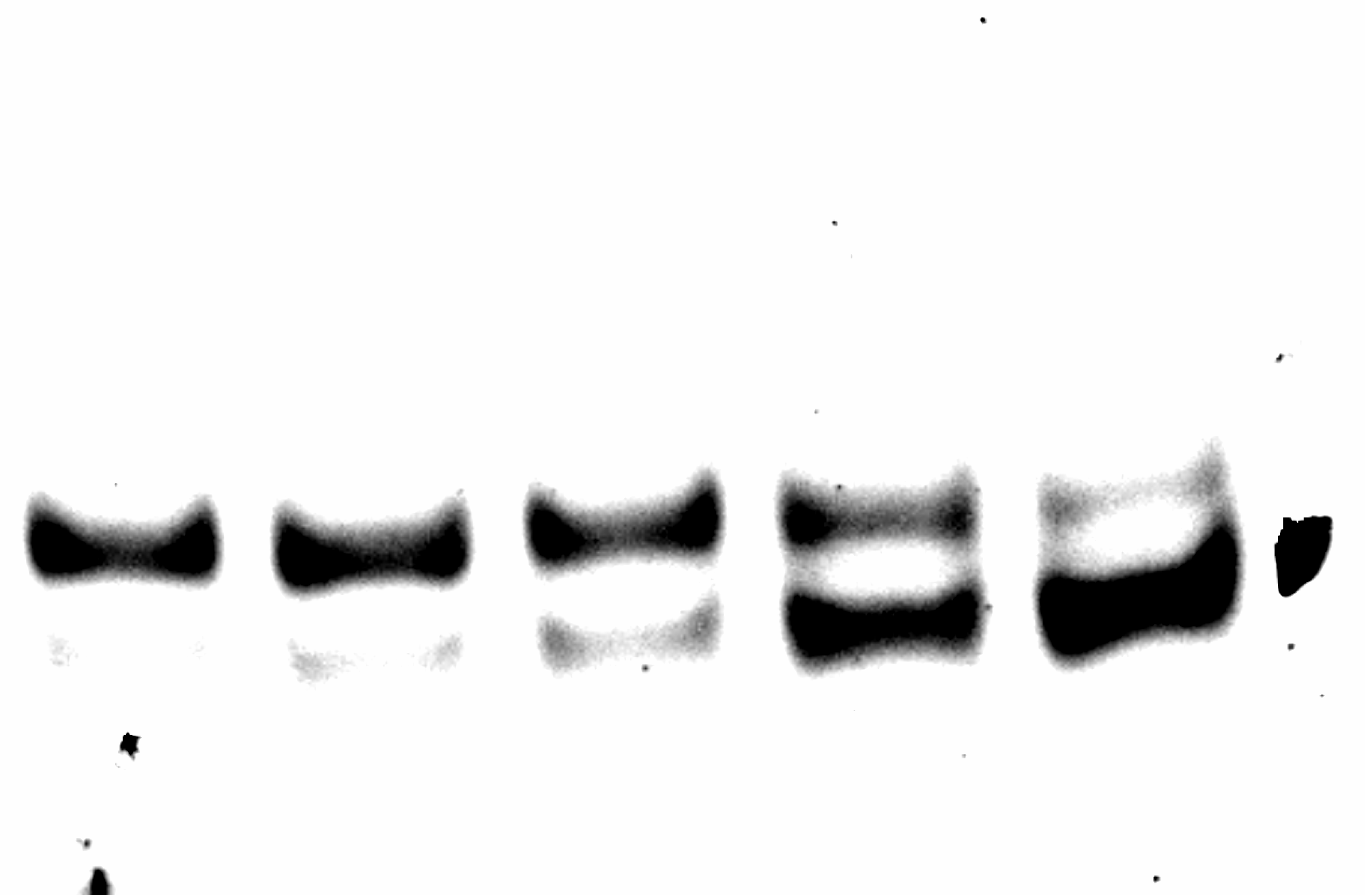

Supplement: Supplementary file 4 [file DataSheet2.ZIP › The original image/OhS12D28,third,The original image.tif]

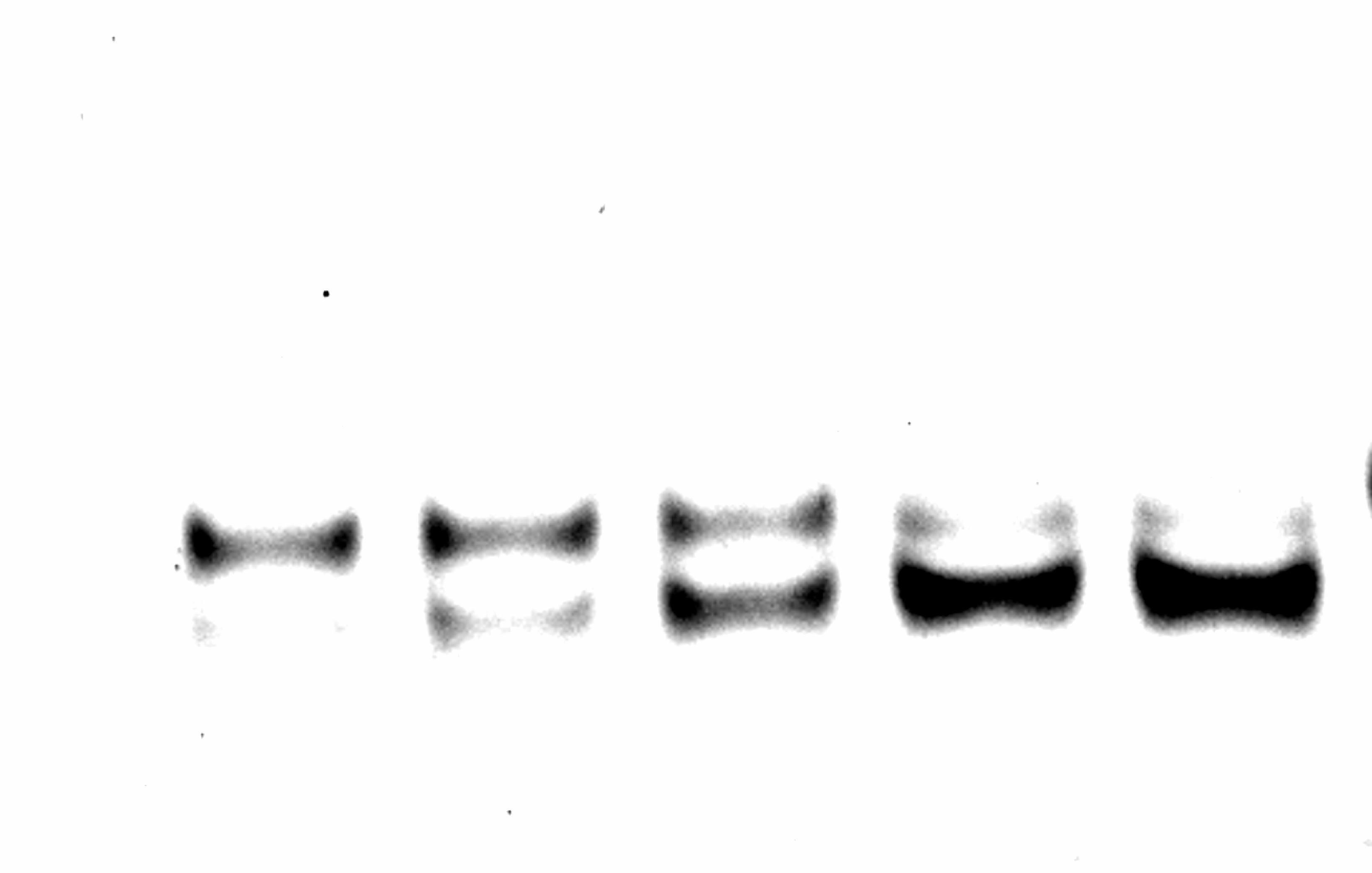

Supplement: Supplementary file 4 [file DataSheet2.ZIP › The original image/OhS20D20,first,The original image.tif]

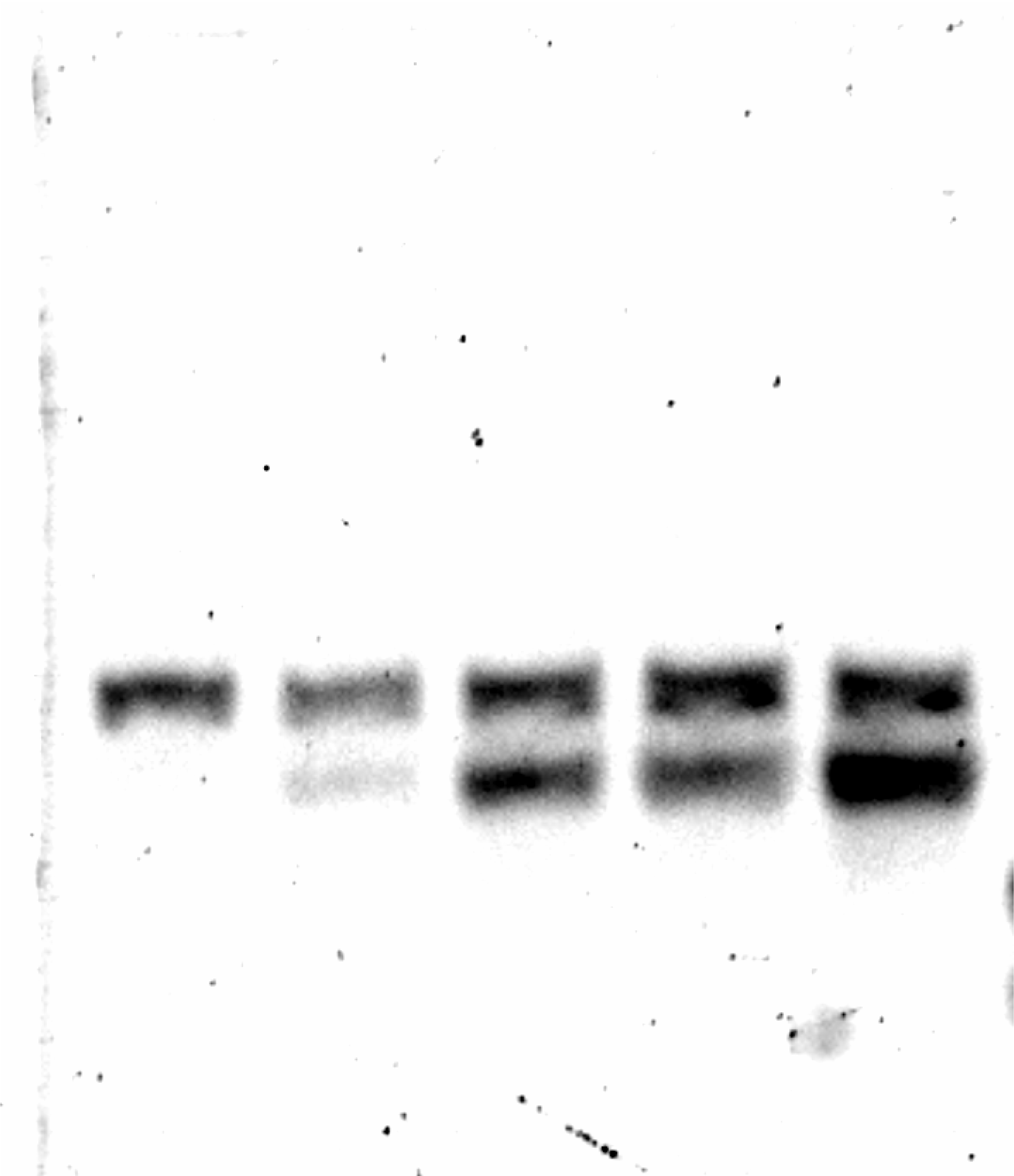

Supplement: Supplementary file 4 [file DataSheet2.ZIP › The original image/OhS20D20,second,The original image.tif]

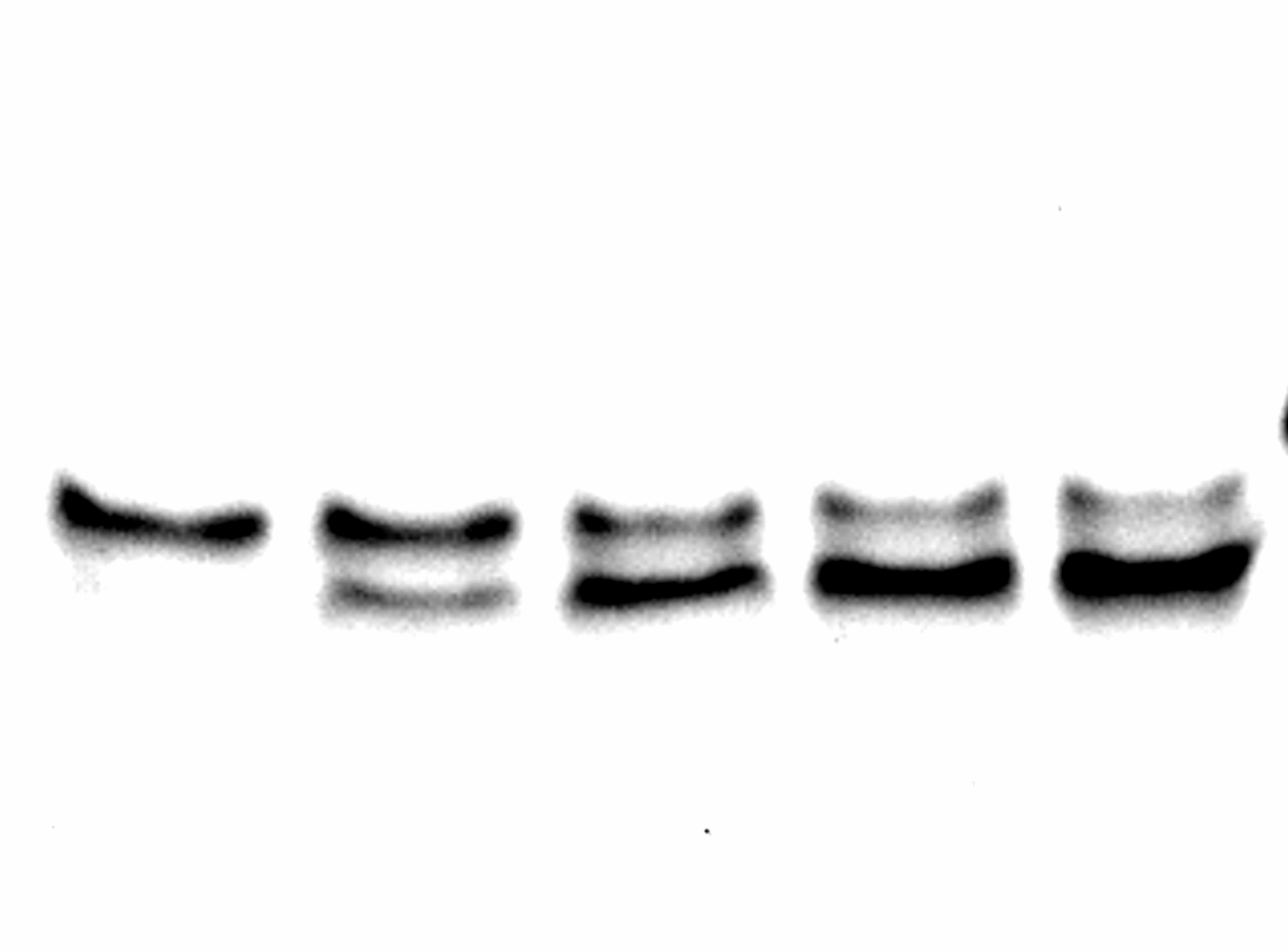

Supplement: Supplementary file 4 [file DataSheet2.ZIP › The original image/OhS20D20,third,The original image.tif]

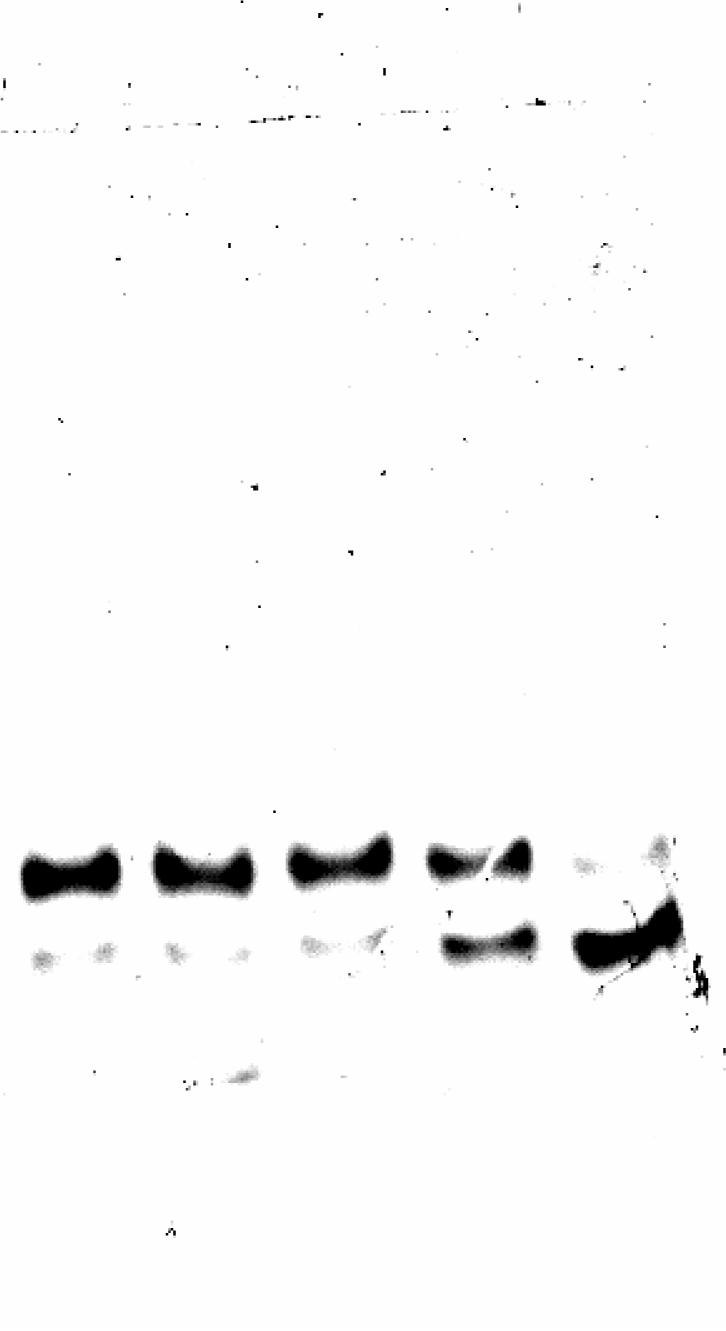

Supplement: Supplementary file 4 [file DataSheet2.ZIP › The original image/OhS4D20,first,The original image.tif]

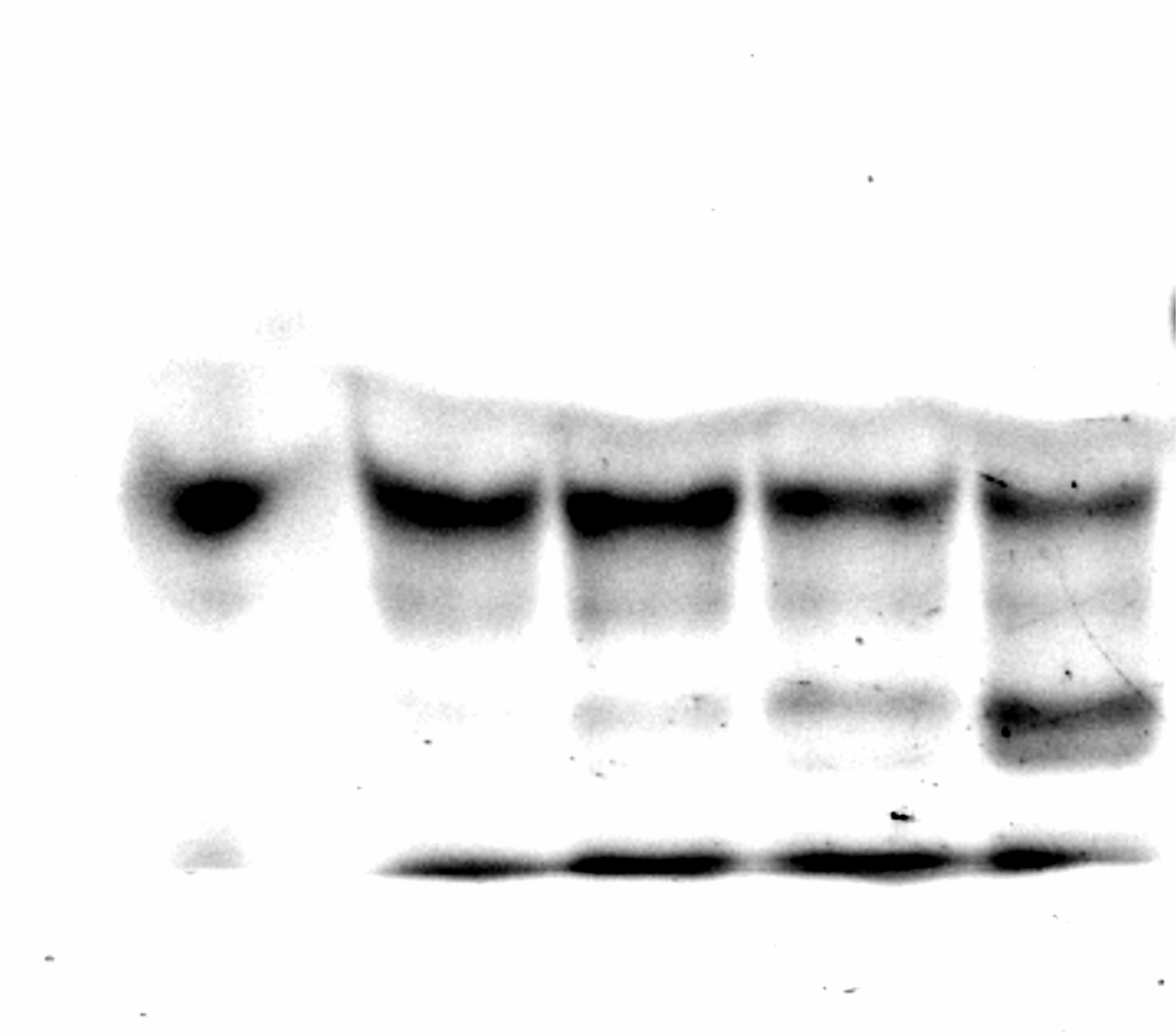

Supplement: Supplementary file 4 [file DataSheet2.ZIP › The original image/OhS4D20,second,The original image.tif]

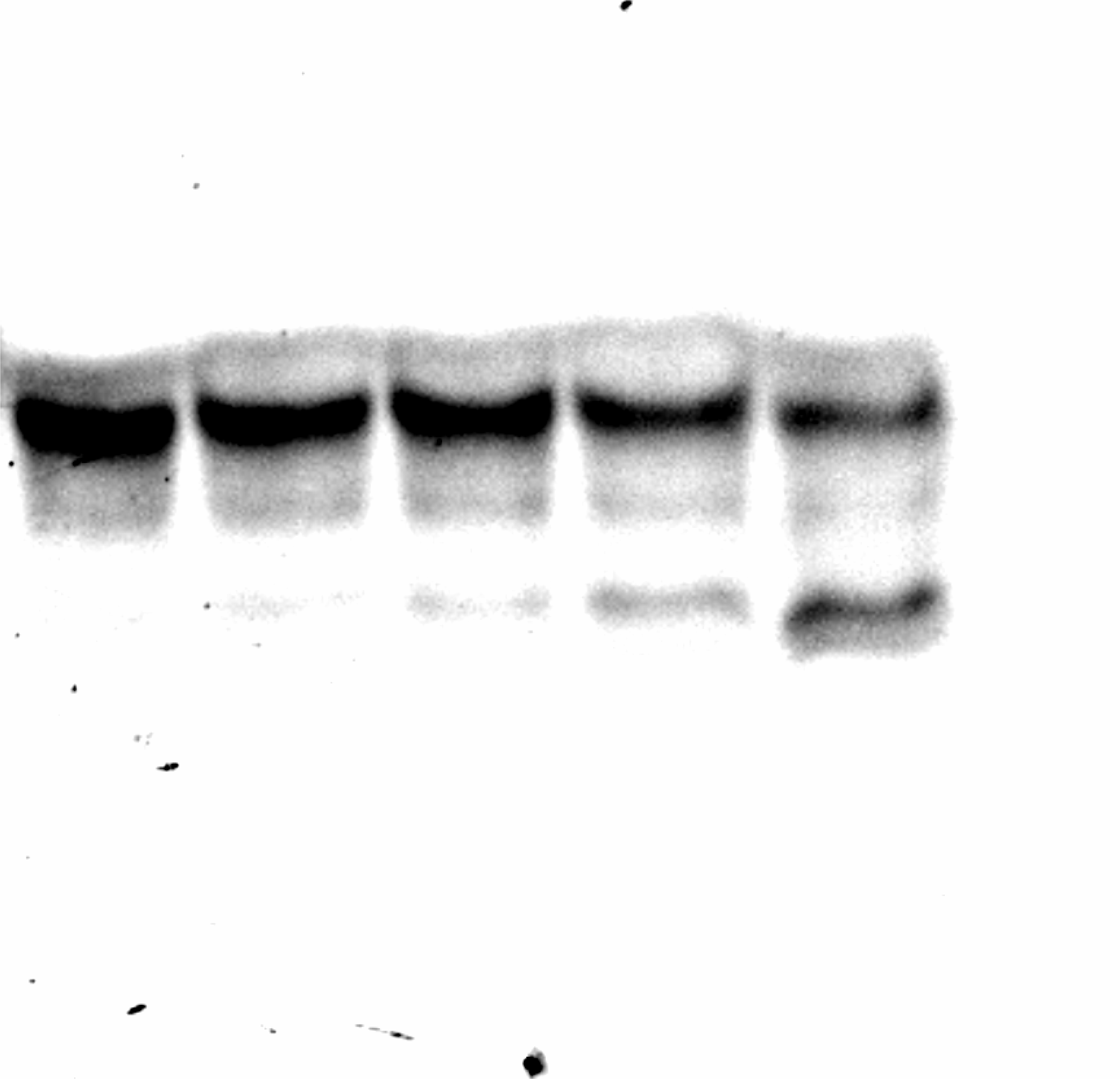

Supplement: Supplementary file 4 [file DataSheet2.ZIP › The original image/OhS4D20,third,The original image.tif]

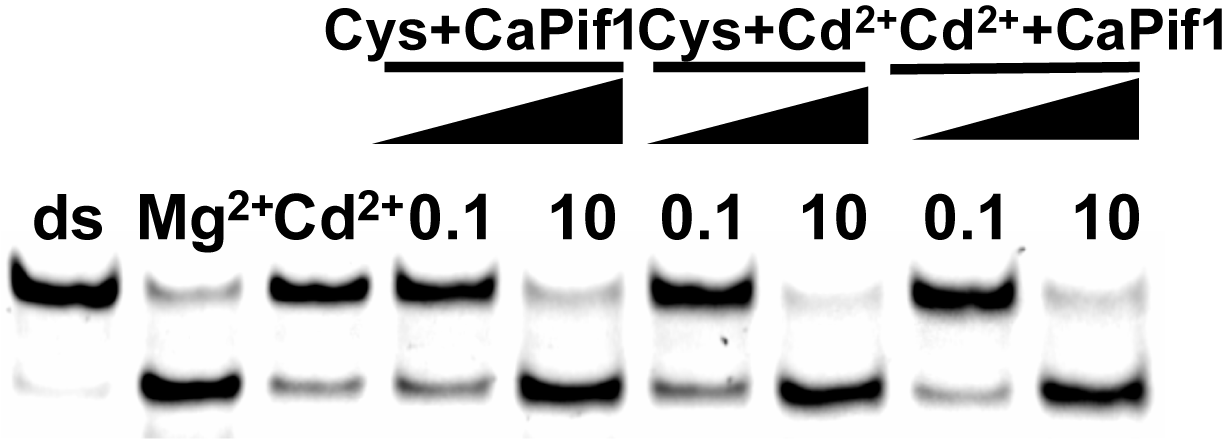

Supplement: Supplementary file 5 [file DataSheet5.ZIP › Supplement 5.tif]

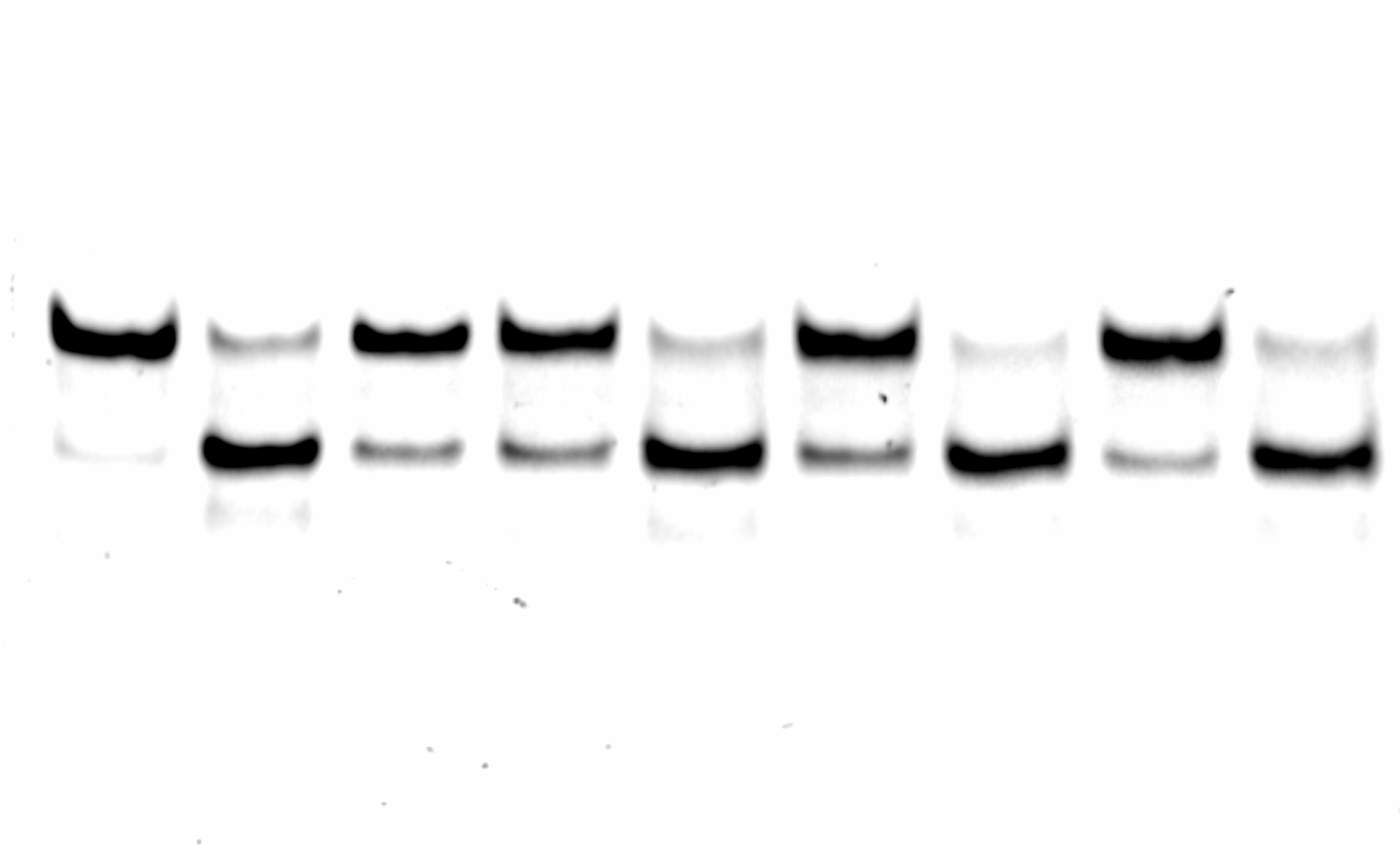

Supplement: Supplementary file 5 [file DataSheet5.ZIP › Supplement5,the original image.tif]
