# Supplementary material for: Mechanistic Insight Into Cadmium- and Zinc-Induced Inactivation of the Candida albicans Pif1 Helicase
Source: Front Mol Biosci. 2022 Jan 21;8:778647. doi: 10.3389/fmolb.2021.778647 (PMC8815974; doi:10.3389/fmolb.2021.778647)
Supplement: Supplementary file 2 [file DataSheet4.ZIP › Supplement 4.docx]

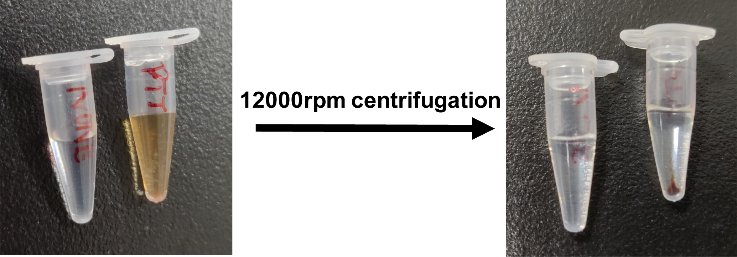


**Supplement 4-1.** The reaction comparison before and after adding Ni^2+^, after 12000rpm centrifugation, the sediment is clearly visible at the bottom of the centrifuge tube.

First, Ni^2+^ research showed the same result as Cd^2+^, and it also demonstrated a strong inhibition. That Ni^2+^ inhibited the unwinding activity of CaPif1 at 200 μM over 90% (Supplement 4-2, A). (Due to precipitation, the concentration is not accurate)

Second, In the experiment that Ni^2+^ was incubated with the four amino acids (Cys, His, Val, Leu) , respectively, CaPif1 was added and incubated for 5 minutes at 30℃, and finally ATP was added to start the reaction. The performance of Ni^2+^ is similar to that of Zn^2+^, and the inhibited unwinding activity effect was not significantly affected (Supplement 4-2, B).

Third, we tested whether EDTA could restore the unwinding activity inhibited by Ni^2+^ metal: firstly, Ni^2+^ was incubated with CaPif1, and then Ni^2+^ was chelated with EDTA, finally Mg^2+^ and ATP were added to the mixture to initiate the spin reaction. The results showed that the inhibition effect of Ni^2+^ was not counteracted by EDTA (Supplement 4-2, C, lane 6 and lane 7), we speculate that Ni^2+^ may damage the structure of CaPif1 irreversibly and render it biologically inactive as the same as the inhibition of Cd^2+^.

Fourth, the detailed experimental procedure has been added to the manuscript. Simultaneously, we also supplement the effect of different adding order of Zn^2+^, Ni^2+^ or Cd^2+^ on CaPif1 unwinding activity inhibited. Let’s take Zn^2+^ as an example, at the same method. Firstly, add only 1.5mM Mg^2+^ to the reaction solution containing CaPif1, incubate at 30°C for 5 min, then add 1.5mM Zn^2+^, continue to incubate at 30°C for 5 minutes, and finally add 5mM ATP to start the reaction. Secondly, incubate CaPif1 with 1.5 mM Zn^2+^, then add 1.5mM Mg^2+^. Third, the reaction solution contained both 1.5 mM Zn^2+^ and 1.5 mM Mg^2+^, add CaPif1. All three methods are incubated at 30℃ for 5 minutes, and finally add 5mM ATP to start the reaction. From the results that no matter how we change the order of adding samples, the three ions are inhibited to CaPif1 unwinding activity, which implies that it may be not due to binding competition between different metals (Supplement 4-2, D). It may be that these three ions are indeed to a certain extent damage to the structure of CaPif1, resulting in reduced or lost unwinding activity.


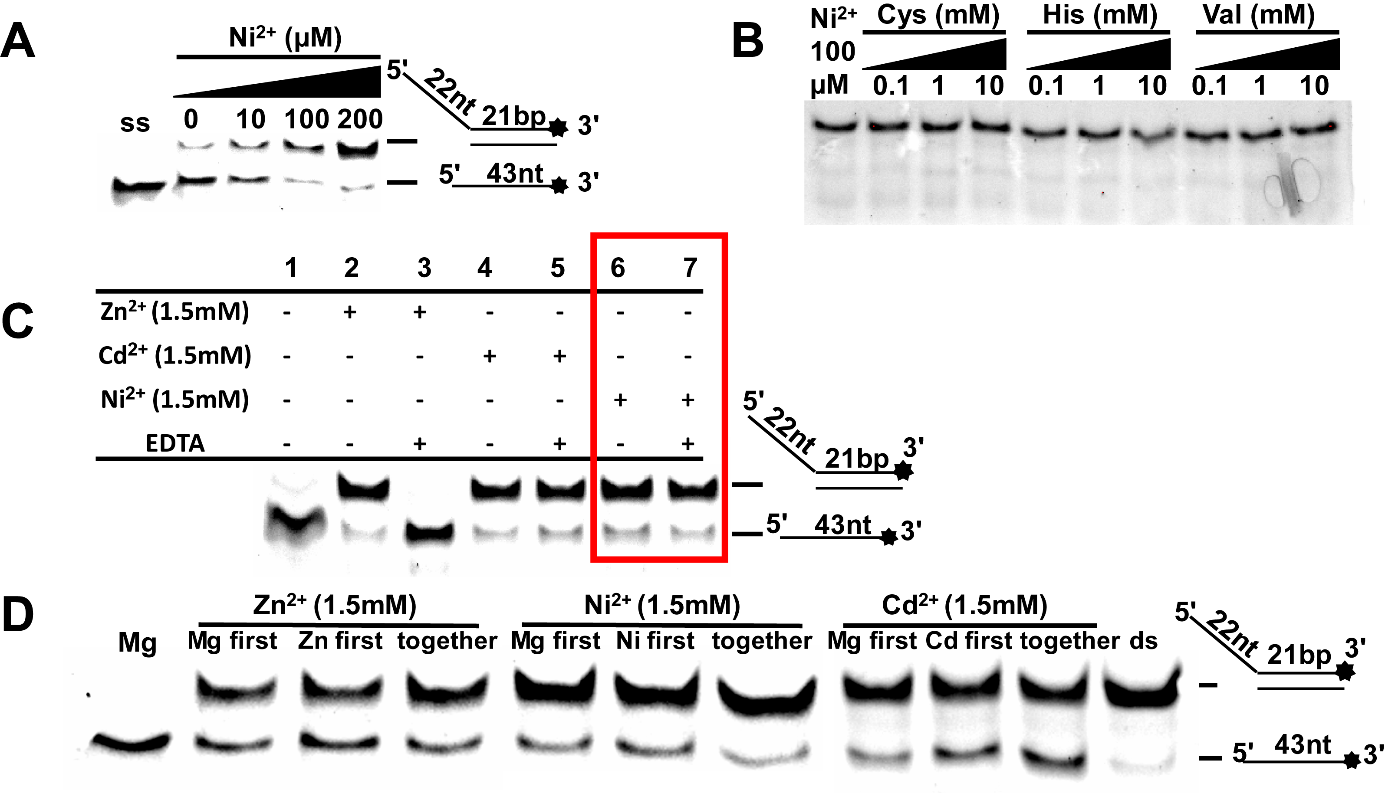


**Supplement 4-2.** The effects of metals on the unwinding activity of CaPif1. (A) The effect of low concentration of Ni^2+^ on DNA (OhS22D21) unwinding activity. (B) CaPif1 (25 nM) was reacted with the DNA (10 nM) in the presence of the indicated amino acids. Lane 1: The control group containing Ni^2+^ (100 μM) without amino acid; 0.1, 1 and 10 represent the amino acid concentration. (C) Lane 1: The control group containing 1.5 mM Mg^2+^ without Ni^2+^. Lane 6: 1.5 mM Ni^2+^ without Mg^2+^; Lane 7: After EDTA chelates all divalent ions, 1.5 mM MgCl_2_ is added. (D) Lane 1: The control group containing 1.5 mM Mg^2+^ without Zn^2+^, Ni^2+^ or Cd^2+^; Mg^2+^ first: the order of sample addition is that Mg^2+^ is incubated with CaPif1 first, and then Zn^2+^/Ni^2+^/Cd^2+^ is added; Zn^2+^ (Ni^2+^ or Cd^2+^) first: CaPif1 is first incubated with the identified metal ion, and then Mg^2+^ is added; Together: CaPif1 is incubated with Mg^2+^ and Zn^2+^ (Ni^2+^ or Cd^2+^) simultaneously
