# Supplementary material for: Mechanistic Insight Into Cadmium- and Zinc-Induced Inactivation of the Candida albicans Pif1 Helicase
Source: Front Mol Biosci. 2022 Jan 21;8:778647. doi: 10.3389/fmolb.2021.778647 (PMC8815974; doi:10.3389/fmolb.2021.778647)
Supplement: Supplementary file 3 [file DataSheet1.ZIP › Supplement 1.docx]

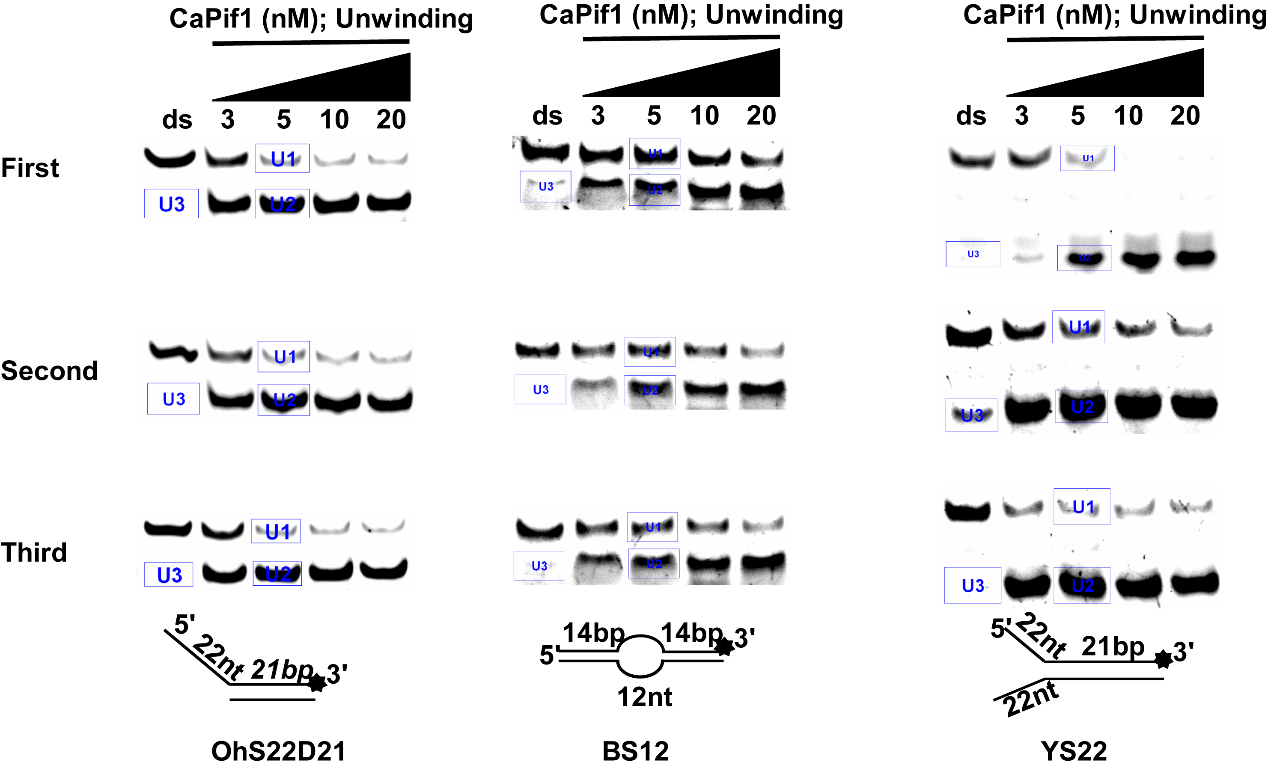


**Supplement 1.**DNA was quantitated as shown above by using the Image Lab software (Bio-Rad) to get the adjusted volume (See file“Supplement 1,Grey value.xlsx”for details), and using it to calculate the fraction using the following formula：$\%unwinding=100\times\frac{P}{S+P}$, where P is the product and S is the substrate. Take OhS22D21 as an example： $\% unwinding=100\times\frac{U2－U3}{U1＋U2－U3}$.where U2 is the product, U1 is the substrate, U3 is the spontaneously unwind product, U2-U3 is the CaPif1 unwinding product.

| **Unwinding (%)** | **Average** | **Stdev** |
| --- | --- | --- |
| **OhS22D21 (5nM)** | 74.01 | 0.84 |
| **BS12 (5nM)** | 39.68 | 2.92 |
| **YS22 (5nM)** | 72.20 | 8.52 |

**Supplement 1, table.** The original data of the unwinding ratio
