# Supplementary material for: Mechanistic Insight Into Cadmium- and Zinc-Induced Inactivation of the Candida albicans Pif1 Helicase
Source: Front Mol Biosci. 2022 Jan 21;8:778647. doi: 10.3389/fmolb.2021.778647 (PMC8815974; doi:10.3389/fmolb.2021.778647)
Supplement: Supplementary file 4 [file DataSheet2.ZIP › Supplement 2.docx]

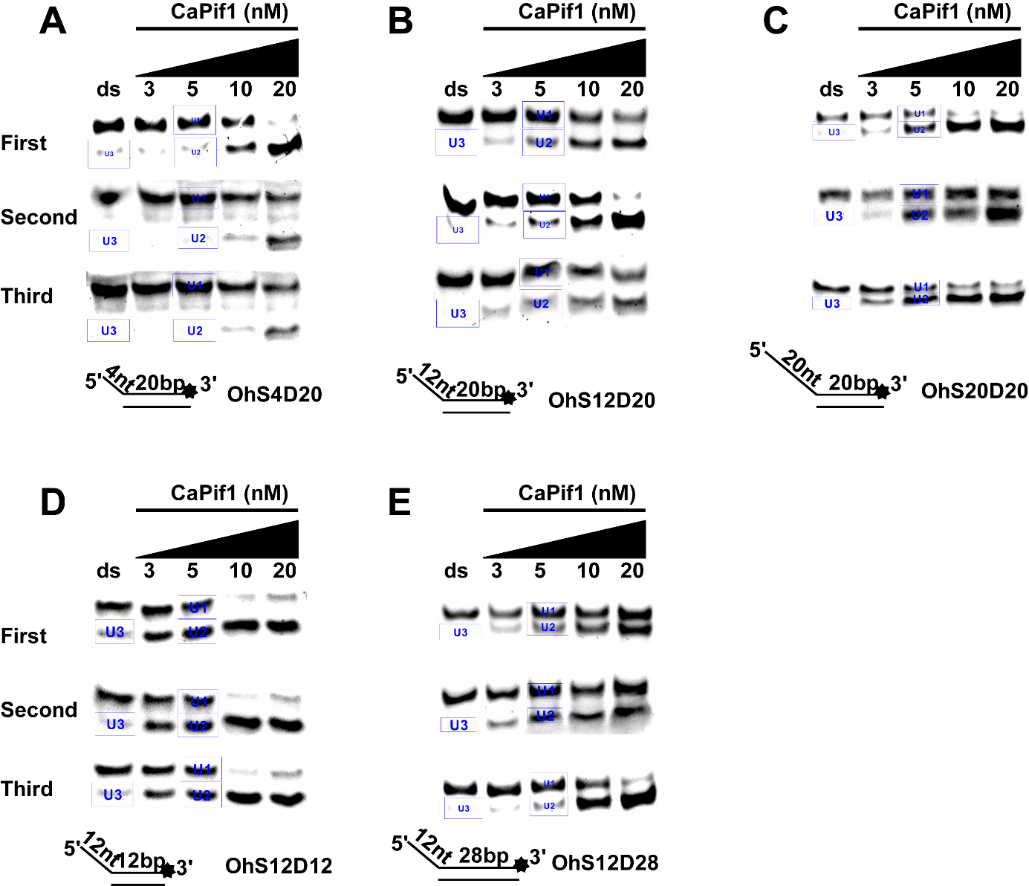


**Supplement 2.** DNA was quantitated as shown above by using the Image Lab software (Bio-Rad) to get the adjusted volume (See file “Supplement 2, Grey value.xlsx”for details), and using it to calculate the fraction using the following formula: $\%unwinding=100\times\frac{P}{S+P}$, where P is the product and S is the substrate. Take OhS4D20 as an example: $\% unwinding=100\times\frac{U2－U3}{U1＋U2－U3}$.where U2 is the product, U1 is the substrate, U3 is the spontaneously unwind product, U2-U3 is the CaPif1 unwind product.

**Supplement 2, table.** The original data of the unwinding ratio

| **Unwinding (%)** | **First** | **Second** | **Third** | **Average** | **Stdev** |
| --- | --- | --- | --- | --- | --- |
| **OhS4D20** | **4.02588** | **9.9305** | **9.8353** | **7.962293** | **3.409034** |
| **OhS12D20** | **29.92035** | **29.80055** | **27.13523** | **28.95204** | **1.574549** |
| **OhS20D20** | **49.51506** | **48.10526** | **53.53143** | **50.38392** | **2.815494** |
| **OhS12D12** | **39.02708** | **38.6924** | **34.9246** | **37.54803** | **2.278111** |
| **OhS12D28** | **23.77703** | **31.13082** | **21.11487** | **25.34091** | **5.18788** |
