# Supplementary material for: Mechanistic Insight Into Cadmium- and Zinc-Induced Inactivation of the Candida albicans Pif1 Helicase
Source: Front Mol Biosci. 2022 Jan 21;8:778647. doi: 10.3389/fmolb.2021.778647 (PMC8815974; doi:10.3389/fmolb.2021.778647)
Supplement: Supplementary file 5 [file DataSheet5.ZIP › Supplement 5.docx]

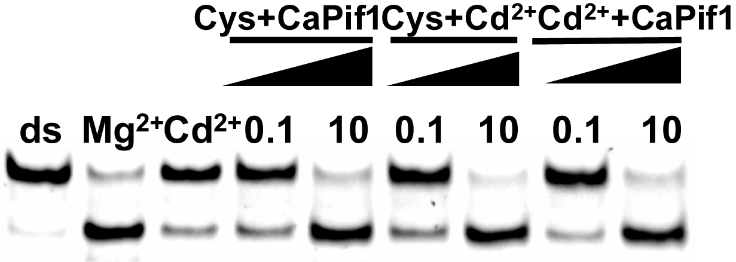


**Supplement 5.**The alleviating effect of cysteine on the inhibition of CaPif1 helicase activity by Cd^2+^. Lane 2: The control group containing 1.5 mM Mg^2+^ without Cd^2+^. Lane 3: 10 μM Cd^2+^ without Mg^2+^. Lane 4-5: Cys is incubated with CaPif1 first, and then Cd^2+^ is added; Lane 6-7: Cd^2+^ is first incubated with Cys, and then CaPif1 is added; Lane 8-9: Cd^2+^ is incubated with CaPif1 first, and then Cys is added. 0.1, 10 represent the amino acid concentration (mM).
